# Functional status and spatial architecture of tumor-infiltrating CD8+ T cells are associated with lymph node metastases in non-small cell lung cancer

**DOI:** 10.1186/s12967-023-04154-y

**Published:** 2023-05-12

**Authors:** Guanqun Yang, Siqi Cai, Mengyu Hu, Chaozhuo Li, Liying Yang, Wei Zhang, Jujie Sun, Fenghao Sun, Ligang Xing, Xiaorong Sun

**Affiliations:** 1 Shandong University Cancer Center, Shandong University, Jinan, Shandong China; 2 Shandong Cancer Hospital and Institute, Shandong First Medical University and Shandong Academy of Medical Sciences, Jinan, Shandong China; 3 School of Clinical Medicine, Weifang Medical University, Weifang, Shandong China; 4 Shandong Cancer Hospital and Institute and Shandong Academy of Medical Science, Jinan, Shandong China; 5 Department of Pathology, Shandong Cancer Hospital and Institute, Shandong First Medical University and Shandong Academy of Medical Science, Jinan, Shandong China; 6 Department of Radiation Oncology, Shandong Cancer Hospital and Institute, Shandong First Medical University and Shandong Academy of Medical Sciences, Jinan, Shandong China; 7 Department of Nuclear Medicine, Shandong First Medical University and Shandong Academy of Medical Sciences, Shandong Cancer Hospital and Institute, No.440, Jiyan Road, Huaiyin District, Jinan, 250117 Shandong China

**Keywords:** CD8 + T cell, Spatial architecture, Dysfunction, Tumor microenvironment, Multiplex immunofluorescence, Lung cancer

## Abstract

**Background:**

Anti-PD-(L)1 immunotherapy has been recommended for non-small cell lung cancer (NSCLC) patients with lymph node metastases (LNM). However, the exact functional feature and spatial architecture of tumor-infiltrating CD8 + T cells remain unclear in these patients.

**Methods:**

Tissue microarrays (TMAs) from 279 IA-IIIB NSCLC samples were stained by multiplex immunofluorescence (mIF) for 11 markers (CD8, CD103, PD-1, Tim3, GZMB, CD4, Foxp3, CD31, αSMA, Hif-1α, pan-CK). We evaluated the density of CD8 + T-cell functional subsets, the mean nearest neighbor distance (mNND) between CD8 + T cells and neighboring cells, and the cancer-cell proximity score (CCPS) in invasive margin (IM) as well as tumor center (TC) to investigate their relationships with LNM and prognosis.

**Results:**

The densities of CD8 + T-cell functional subsets, including predysfunctional CD8 + T cells (T_predys_) and dysfunctional CD8 + T cells (T_dys_), in IM predominated over those in TC (*P* < 0.001). Multivariate analysis identified that the densities of CD8 + T_predys_ cells in TC and CD8 + T_dys_ cells in IM were significantly associated with LNM [OR = 0.51, 95%CI (0.29–0.88), *P* = 0.015; OR = 5.80, 95%CI (3.19–10.54), *P* < 0.001; respectively] and recurrence-free survival (RFS) [HR = 0.55, 95%CI (0.34–0.89), *P* = 0.014; HR = 2.49, 95%CI (1.60–4.13), *P* = 0.012; respectively], independent of clinicopathological factors. Additionally, shorter mNND between CD8 + T cells and their neighboring immunoregulatory cells indicated a stronger interplay network in the microenvironment of NSCLC patients with LNM and was associated with worse prognosis. Furthermore, analysis of CCPS suggested that cancer microvessels (CMVs) and cancer-associated fibroblasts (CAFs) selectively hindered CD8 + T cells from contacting with cancer cells, and were associated with the dysfunction of CD8 + T cells.

**Conclusion:**

Tumor-infiltrating CD8 + T cells were in a more dysfunctional status and in a more immunosuppressive microenvironment in patients with LNM compared with those without LNM.

**Supplementary Information:**

The online version contains supplementary material available at 10.1186/s12967-023-04154-y.

## Introduction

Lymph node metastases (LNM) is a major prognostic factor and determines treatment for operable non-small cell lung cancer (NSCLC) patients [1, 2]. LNM positive NSCLC patients with PD-L1 > 1% have been recommended to use anti-PD-(L)1 immunotherapy, but its efficacy is only 20–40% [3]. Moreover, it is estimated that there are about 20% NSCLC patients missing the optimal window for immunotherapy due to ambiguous lymph node states [4]. The main function of anti-PD-(L)1 immunotherapy is to activate CD8+ T cells, the central role in immune-mediated control of cancer [5]. Consequently, it is imperative to enhance our understanding of CD8+ T cells in NSCLC patients, especially those with LNM, prompting precise immunotherapy.

The anti-tumor effect of CD8+ T cells largely depends on their spatial architecture and functional status. The spatial structure of tumor-infiltrating CD8+ T cells, such as the topologically distinct distribution and spatial interplay with other neighboring cells, determine the prognosis and treatment response of patients. Previous studies demonstrated that high density of CD8+ T cells in tumor center (TC) as well as invasive margin (IM) was associated with good prognosis of patients with NSCLC, while some studies believed that CD8+T cells in different regions embodied inconsistent prognostic significance, suggesting that there were some unexpected causes [6–9]. Several studies reported that a higher density of CD8+ T cells neighboring CD8- T cells or regulatory T cells was associated with better prognosis of patients with NSCLC, indicating the potential significance of the CD8+ T cells limited in certain spatial scale [10, 11]. Additionally, Peng et al. provided a novel insight to decode spatial information of immune cell, through variables based on the intercellular distance reflecting the level of interaction [12]. Therefore, a comprehensive decipherment of the spatial structure is necessary for intratumoral CD8+ T cells in NSCLC.

The functional status of CD8+ T cells is modified by the expression of inhibitory molecules, which can be used to distinguish CD8+ T cells with different states [13]. PD-1 has been used as an indicator of dysfunction of CD8+ T cells and PD-1+CD8+ T cells was thought as a biomarker to predict better response for immunotherapy and longer survival of patients, while some studies hold contradictory views [14–18]. Using single-cell sequencing, Guo et al. identified a variety of CD8+ T-cell functional subsets via multiple transcriptional markers, such as PD-1, CD103 and Tim3, in NSCLC, and demonstrated that a high ratio of predysfunctional to dysfunctional T cells correlated with better prognosis in 11 patients with adenocarcinoma [19]. However, these conclusions are limited by the lack of adequate sample size and histopathological studies reflecting the real infiltration.

The infiltration of CD8+ T cells are regulated by other stromal cells in the tumor microenvironment (TME). Some studies have demonstrated that endothelial cells of cancer microvessels (CMVs) can reduce the ability of CD8+ T cells to adhere to the vasculature and induce apoptosis of CD8+ T cells [20, 21]. However, their effect on the functional status of intratumoral CD8+ T cells in tumor remains unclear. Grout et al. found that cancer-associated fibroblasts (CAFs) obstruct T cells from lung cancer cells [22], but it is unclear whether these barriers are unselective for immune cells and are able to affect their functional states.

Despite the importance of understanding the functional status and spatial architecture of CD8+ T cells, traditional single-color immunofluorescence or immunohistochemistry methods are unable to distinguish complex cell subsets, whereas prevalent flow cytometry and single-cell transcriptome sequencing methods abandon important spatial information [23, 24]. Multiplex immunofluorescence (mIF), enabling to stain cells by multi-protein labels in situ, provides an opportunity to analyze the spatial characteristics of CD8+ T cell functional subsets.

To elucidate the characteristics of intratumoral CD8+ T cells in NSCLC patients with LNM, using mIF and machine learning-assisted image analysis, we acquired the amount and single-cell localization of CD8+ T-cell functional subsets and other cells in the tumor microenvironment (TME) in 1116 tissue sites from 279 NSCLC patients. We noted that low density of predysfunctional CD8+ T cells (T_pre__dys_) in TC, high density of dysfunctional CD8+ T cells (T_dys_) in IM, and shorter distance from CD8+ T cells to neighboring cells were significantly associated with LNM and worse prognosis. CMVs and CAFs might act as “selective barriers” and were correlated with the dysfunction of CD8+T cells.

## Methods

### Patient cohorts

We retrospectively observed patients with NSCLC who underwent radical surgery between January 2014 and October 2018 at Shandong Cancer Hospital. Patients who received neoadjuvant therapy were excluded in this study. All tissue specimens from each patient were reviewed before and after the experiment, and only those with at least one available tissue left (n=279) were included in the final analysis (Additional file 1: Figure S1). Clinical information was obtained from the electronic medical records. This study was approved by the Ethics Review Board of Shandong Cancer Hospital.

### Tumor specimens and tissue microarrays

**Fig. 1:**
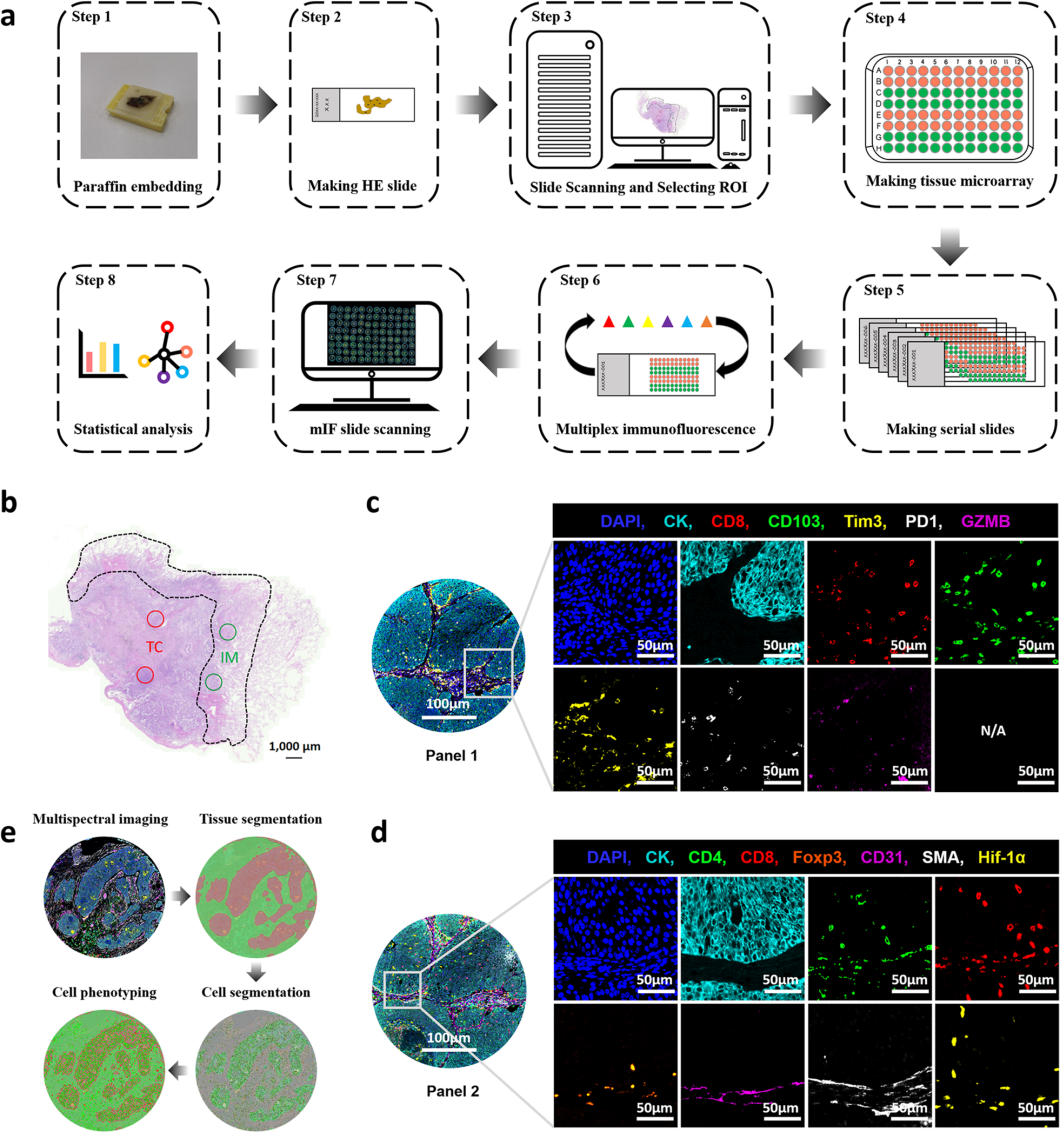
Identification and characterization of tumor microenvironment in non-small cell lung cancer. **a** Schematic representation of the experimental design and analytical methods used in this study. **b** Selection of the region of interest (ROI). IM, invasive margin; TC, tumor center. Representative composite images displaying multiplex staining of Panel 1 (**c**) and Panel 2 (**d**) after multispectral imaging. Each of the single-marker component image in the composite image after spectral unmixing was displayed in the enlarged subsection

Tissue microarrays (TMA) were constructed from formalin-fixed, paraffin-embedded (FFPE) tissues of the patient cohort (Fig. 1a), using 1 mm diameter tissue cores with the TMA Grand Master System (3DHistech, Hungary). For each patient, two cores from representative areas of the tumor center (TC) and two cores from representative areas of the invasive margins (IM) were collected for TMA construction (Fig. 1b). IM was defined as the region centered on the border separating malignant tissue from uninvolved tissue, extending 1 mm in all directions [25–27]. A tissue core was excluded only if no analyzable tissue was present or if staining failed.

### Multiplex immunofluorescence staining

mIF staining was performed using the Akoya-Opal-Kit (Akoya Biosciences, USA; cat. NEL871001KT) according to the manufacturer’s instructions. In brief, 3 μm slides obtained from the TMA blocks were deparaffinized with xylene, rehydrated through a graded ethanol series, and were performed antigen retrieval with citric acid buffer (pH 6.0)/ethylene diamine tetra acid buffer (PH 8.0) using microwave incubation. Each slide was subjected to six (Panel 1) or seven (Panel 2) sequential rounds of staining, each including antigen blocking with 1% BSA followed by a specific primary antibody and a corresponding horseradish peroxidase-conjugated secondary antibody. A horseradish peroxidase-conjugated secondary antibody mediated the covalent binding of a predetermined fluorophore using tyramide signal amplification (TSA). This covalent reaction was followed by additional antigen retrieval to remove bound antibodies before the next antigen block. After all serial reactions, the multiplex stained slides were counterstained with DAPI and scanned with the Vectra Polaris Scanner System (PerkinElmer, USA) (Fig. 1a). Two panels of multiplex staining were prepared on serial TMA slides (Fig. 1c, d). Detailed information about all primary antibodies used and mixed multispectral images for each TMA after scanning are provided in Additional file 1: Table S1 and Additional file 2.

### Machine learning-assisted multispectral image processing

Multispectral images were processed using the Inform software 2.4.8 (PerkinElmer, USA) (Fig. 1e). Firstly, the mixed multispectral images were unmixed into single-spectral images using a fluorescence spectral library previously extracted from singly stained samples for each fluorophore. Unspecific autofluorescence was ascertained using the unstained part within the images and was subtracted from the images before tissue segmentation. Secondly, the tumor compartment and stroma compartment were visually recognized based on the signal of the epithelial cell marker (pan-CK) in at least 10 images in order to train a tissue classifier, and then all images were subjected to segmentation in the trained tissue classifier, the accuracy of which was required to exceed 95% (actually 98.85% in our customized project). Thirdly, given that images contain mixtures of cell types with very different nuclear morphologies and cell morphologies, we chose to combine nuclear signals (DAPI), membrane signals (CD8, CD4, CD31), and cytoplasm signal (CK, αSMA) for adaptive cell segmentation. Lastly, like tissue segmentation, a single-marker phenotype classifier was trained by visually identifying at least 30 positive cells and 30 negative cells for each marker, and then all images were subjected to process in the trained phenotype classifier, whose accuracy was required to exceed 95% (actually over 99.50% in our customized project for each marker). After completing the above steps, single-marker phenotypes and single-cell two-dimensional coordinates can be generated. All hematoxylin-eosin and mIF images were independently reviewed by at least two investigators, including an experienced pathologist (J.S.).

### Establishment of parameters

Quantitative spatial features, including the quantitative and spatial parameters of cells, were applied to unscramble the features of intratumoral CD8+ T cells. A bioinformatics tool (phenoptr 0.3.2; https://github.com/akoyabio/phenoptr) was used to calculate the number of any type of cells defined by the available multiple-marker phenotypes and the nucleus-to-nucleus Euclidean distances between any two types of cells. All cell types we detected and corresponding scheme of phenotypes involved in the study are presented in Additional file 1: Table S2, and all other cell types not defined in our phenotyping scheme (that is, macrophages, mast cells, neutrophils and dendritic cells, et al.) were grouped into one category, labelled as ‘other cells’ [28, 29]. The cell density was calculated via normalizing the corresponding cell counts by the total cell counts (cell/1000 cells):

$$density \,of \,A=n\left(A\right)/n\left(total \,cells\right)\times 1000$$

in which A denoted one type of cell, and n equaled the number of cells. In order to conduct a downstream analysis of CD8+ T cells in the TME, we established two spatial parameters, including the mean nearest neighbor distance (mNND) between CD8+ T cells and neighboring cells and the cancer-cell proximity score (CCPS) of cells. mNND was defined as the mean distance between all A cells and their nearest neighboring B cells across the tissue site, whose formula was:

$$mNND\left(A-B\right)=\sum_{i=n}{d}_{min}\left({A}_{i}-B\right)/n\left(A\right)$$

in which A denoted one type of cell, B denoted another, and d_min_(A_i_-B) equaled the minimum distance from the ith A cell to other B cells. A shorter mNND(A-B) indicated a closer interplay between them. CCPS was defined as the mean number of A cells within a certain radius of all cancer cells across the tissue site, whose formula was:

$$CCPS\left(A\right)=\sum_{i=N}n\left({C}_{i}\stackrel{r}{\to }A\right)/n\left(C\right)$$

in which A denoted one type of cell while C denoted cancer cell, r denoted specified radius and n(Ci $\stackrel{r}{\to }$ A) equaled the number of A cells within radius r-mm of the i-th cancer cell. Therefore, a higher CCPS of A cells indicates a higher density of A cells around the cancer cells within a certain distance, suggesting better intratumoral infiltration to some extent.

### Statistical analysis

The Kolmogorov-Smirnov test determined that the parameters of all cell phenotypes were not normally distributed; therefore, the Mann-Whitney U test for two independent samples and Wilcoxon signed-rank test for paired samples were used to analyze these parameters. Univariate and multivariate logistic regression models (backward elimination) and Cox proportional hazards regression models (backward elimination) were used to estimate the prognostic value of the parameters involved in this study. Survival analysis was performed by Kaplan-Meier curve and Log-rank test in patients grouped by individual parameter. Spearman rank correlation coefficient was used to evaluate the correlation between cancer-cell adjacent CMVs, CAFs and T-cell subsets. The results were considered statistically significant if two-sided *P* < 0.05. All statistical results were verified by at least two independent investigators. Statistical analyses were performed with SPSS 26.0 (IBM, USA) and R software.

## Results

### Clinicopathological characteristics of the NSCLC cohort

A total of 279 patients with stage IA-IIIB NSCLC meeting the inclusion criteria were eventually enrolled, of which 86 cases (30.8%) were confirmed with LNM during the postoperative pathology (Table 1). N1 and N2 accounted for 57% (n=49) and 43% (n=37) of the lymph node positive patients, respectively. Male (65.2%), stage I (55.6%) and lung adenocarcinoma (64.2%) patients comprised the majority of the cases. The median follow-up time was 1064 days. Patients with older age (*P* = 0.002) and those with larger tumor diameters (*P* < 0.001) had a greater propensity for LNM.

**Table 1:** Clinicopathological characteristics of patients with samples subjected to multiplex staining

Characteristic	All patients (N = 279)	Without LNM (n = 193)	With LNM (n = 86)	*P* value
*Age, years*				
≤ 60 > 60 Median (IQR)	124 (44.4%) 155 (55.6%) 62 (57.67)	77 (39.9%) 116 (60.1%) 63 (58.68)	47 (54.7%) 39 (45.3%) 59 (53.65)	**0.002**
*Gender*				
Male Female	182 (65.2%) 97 (34.8%)	125 (64.8%) 68 (35.2%)	57 (66.3%) 29 (33.7%)	0.807
*Smoking index*^a^				
< 400 ≥ 400	156 (55.9%) 123 (44.1%)	109 (56.5%) 84 (43.5%)	47 (54.7%) 39 (45.3%)	0.777
*ECOG PS*				
0–1 > 1	202 (72.4%) 77 (27.5%)	144 (74.6%) 49 (25.3%)	58 (67.4%) 28 (32.5%)	0.147
*Tumor diameter (cm)*				
Median (IQR)	3.0 (2.5, 4.0)	3.0 (2.5, 4.0)	3.5 (2.8, 4.7)	**<0.001**
*Histological type*				
LUSC LUAD Other	95 (34.1%) 179 (64.2%) 5 (1.8%)	61 (31.6%) 128 (66.3%) 4 (2.1%)	34 (39.5%) 51 (59.3%) 1 (1.2%)	0.180
*T stage*				
T1 T2 T3 T4	88 (31.5%) 161 (57.7%) 16 (5.7%) 14 (5.0%)	73 (37.8%) 100 (51.8%) 11 (5.7%) 9 (4.7%)	15 (17.4%) 61 (70.9%) 5 (5.8%) 5 (5.8%)	**0.004**
*N stage*				
N0 N1 N2 N3	193 (69.2%) 49 (17.6%) 37 (13.3%) 0 (0%)	193 (100.0%) 0 (0%) 0 (0%) 0 (0%)	0 (0%) 49 (57.0%) 37 (43.0%) 0 (0%)	**<0.001**
*AJCC Stage*				
IA IB IIA IIB IIIA IIIB	72 (25.8%) 83 (29.7%) 18 (6.5%) 52 (18.6%) 50 (17.9%) 4 (1.4%)	72 (37.3%) 83 (43.0%) 18 (9.3%) 10 (5.2%) 10 (5.2%) 0 (0%)	0 (0%) 0 (0%) 0 (0%) 42 (48.8%) 40 (46.5%) 4 (4.7%)	**<0.001**

LUSC, lung squamous cell carcinoma; LUAD, lung adenocarcinoma; LNM, lymph node metastases; IQR, interquartile range

a, Smoking index = number of cigarettes smoked per day × year(s)

Bold values indicate the significantly different clinicopathological characteristics between patients with and without LNM, determined by Mann-Whitney U test

### The density of CD8 + T-cell subsets in invasive margin predominated over those in tumor center

**Fig. 2:**
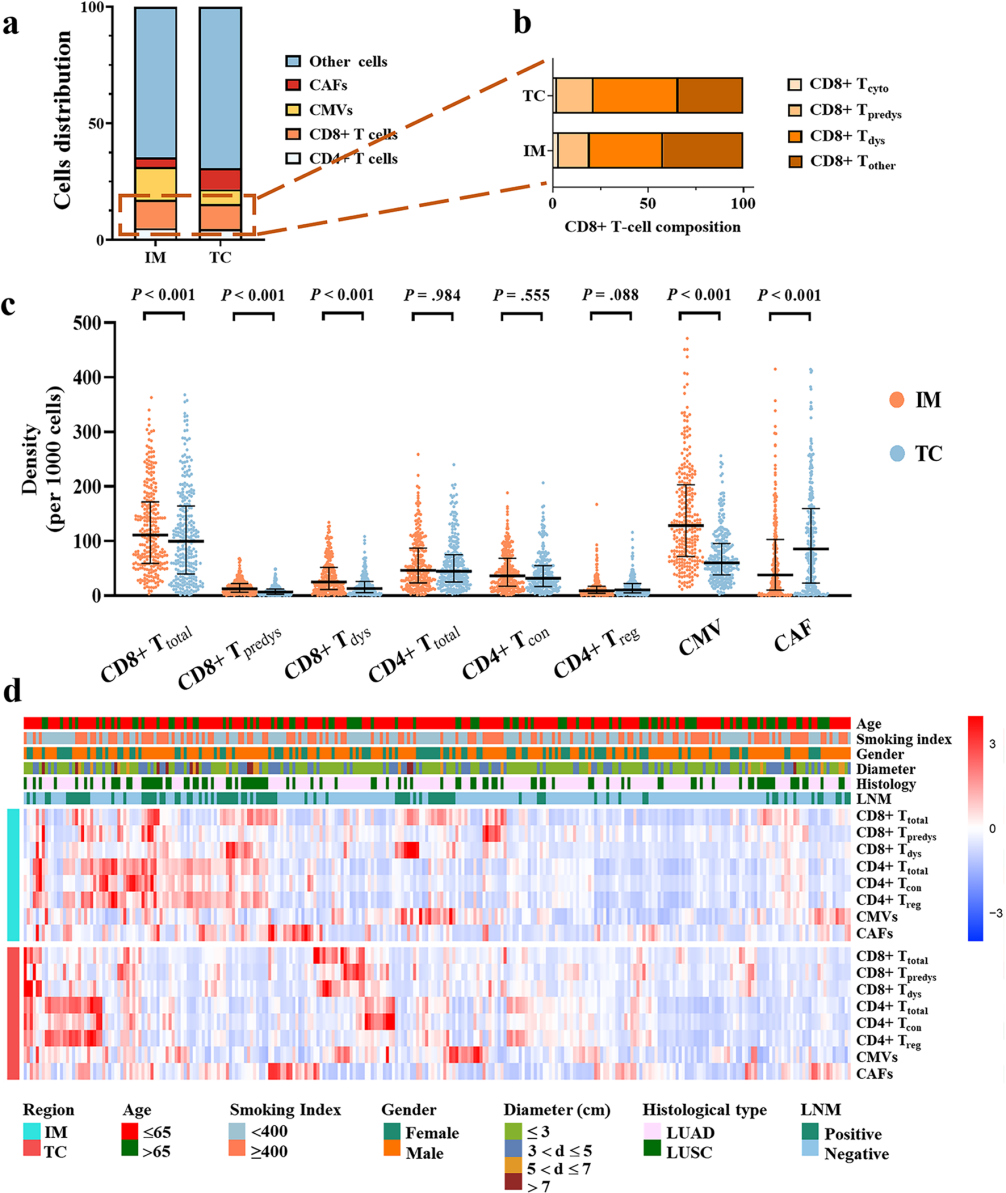
Automated image analysis revealed the discrepancy across regions and patient subgroups. **a** Composition of the stromal cell subsets in invasive margin and tumor center. **b** Composition of CD8 + T-cell subsets. **c** Different densities of cell subsets between invasive margin and tumor center. Significance (*P* value) was determined by paired Wilcoxon signed-rank test. **d** Clinicopathologic characteristics and cell composition of per patient on multiplex immunofluorescence images

In the TME, CD8+ T cells are induced to become dysfunctional via expressing inhibitory molecules, such as PD-1 and CD103 on predysfunctional CD8+ T cells, and Tim3 on terminally dysfunctional CD8+ T cells [13, 16, 19]. Besides, neighboring cells may determine the role of intratumoral CD8+ T cells. Hence, using mIF, we identified CD8+ T-cell functional subsets and other cell populations in the TME, including cancer cells, CD4+ T cells, endothelial cell of cancer microvessels and cancer-associated fibroblasts (Additional file 1: Table S2). To identify the discrepancy in the regional distribution of cell populations detected in the TME, we compared their densities in IM and TC. Any cell population was included in the subsequent analysis only when its density was greater than or equal to 5‰. The relative composition of the stromal cell subsets demonstrated great heterogeneity between the IM and TC (Fig. 2a). Notably, a considerable proportion of CD8+ T cells exhibited a dysfunctional status (Fig. 2b). Specifically, compared with TC, there was higher densities of total CD8+ T cells (CD8+ T_total_) (*P* < 0.001), CD8+ T_predys_ cells (*P* < 0.001), CD8+ T_dys_ cells (*P* < 0.001), and CMVs (*P* < 0.001), and a lower density of CAFs in IM. IM and TC contained nearly equivalent densities of total CD4+ T cells (CD4+ T_total_) cells and conventional CD4+ T cells (CD4+ T_con_). Notably, TC possessed slightly higher density of regulatory CD4+ T cells (CD4+ T_reg_) despite no statistical significance (*P* = 0.088) (Fig. 2c, d).

### Intratumoral CD8 + T cells in patients with LNM were characterized by decreased CD8 + T_predys_ in TC and increased CD8 + T_dys_ in IM

**Fig. 3:**
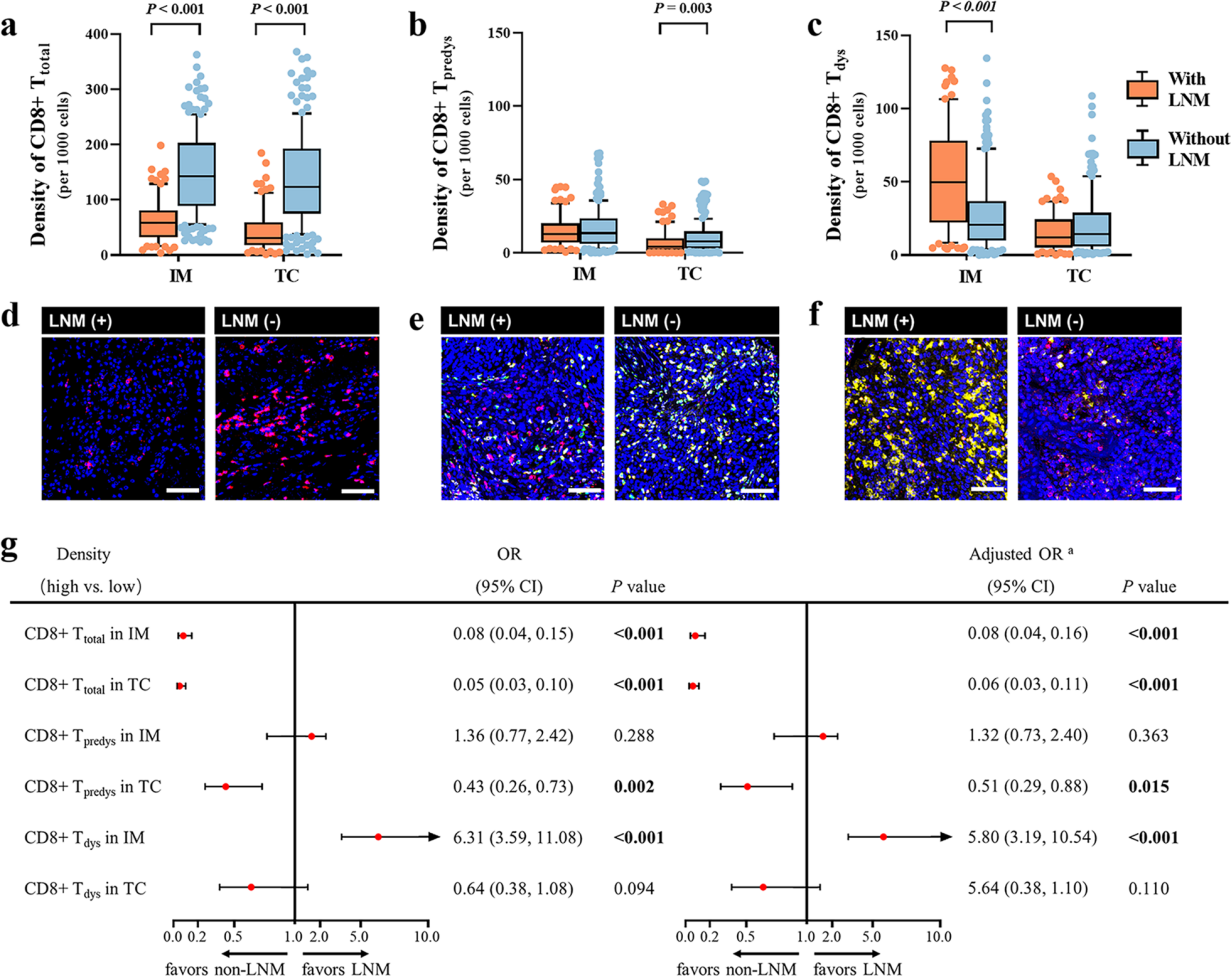
Comparison of the density of intratumoral CD8 + T cells between NSCLC patients with and without LNM. Discrepancies of the densities of CD8 + T_total_ cells (**a**), CD8 + T_predys_ cells (**b**), CD8 + T_dys_ cells (**c**) between NSCLC patients with and without LNM. Significance (*P* value) was determined by Mann–Whitney U test. **d**–**f** Representative multiplex immunofluorescence images (left) and corresponding phenotype map (right) exhibited the discrepancies correspondingly. **g** Forest plots of univariate and multivariate logistic models demonstrated the relationships between the density of CD8 + T-cell functional subsets and LNM. ^a^The multivariate logistic regression model adjusted age (≤ 60 years vs. > 60 years), gender (male vs. female), histology subtype (squamous cell carcinoma vs. adenocarcinoma) and tumor diameter (≤ 3 cm vs. > 3 cm)

As mentioned previously, lymph node status is so crucial for NSCLC that it is the focus of this study. We compared the density of intratumoral CD8+ T-cell subsets in patients with and without LNM. The density of CD8+ T_total_ cells was remarkably decreased in both IM (*P* < 0.001) and TC (*P* < 0.001) in patients with LNM (Fig. 3a). More importantly, a lower density of CD8+ T_predys_ cells in TC (*P* = 0.003) (Fig. 3b) and a higher density of CD8+ T_dys_ cells in IM (*P* < 0.001) (Fig. 3b) were observed in patients with LNM. Representative mIF images intuitively displayed heterogeneous density of intratumoral CD8+ T-cell subsets in NSCLC patients with LNM versus those without LNM (Fig. 3d–f).

Beyond lymph node status, we also compared the density of CD8+ T-cell functional subsets in the context of other distinct clinicopathological factors. Generally, there were few significant differences in patients grouped by age and performance status with respect to densities of CD8+ T-cell functional subsets, regardless of the compartment, while the density of CD8+ T_total_ cells was higher in patients with older age (*P* = 0.012 in TM; *P* = 0.030 in TC) and worse performance status (*P* = 0.002 in IM; *P* = 0.025 in TC) (Additional file 1: Tables S3, S4). Compared with LUAD, LUSC showed significantly higher densities of CD8+ T_predys_ cells (*P* = 0.007) and T_dys_ cells in IM (*P <* 0.001), and a lower density of CD8+ T_predys_ cells in TC (*P* = 0.001). Similar discrepancies were in accord with male patients and moderate-to-severe smoking patients (smoking index ≥ 400), consistent with the patient preference for LUSC. Moreover, patients with a more advanced T stage showed lower a density of CD8+ T_total_ cells in IM (*P <* 0.001) and TC (*P <* 0.001) and lower density of CD8+ T_predys_ cells in TC (*P* = 0.010), but a higher density of T_dys_ cells in IM (*P <* 0.001) (Additional file 1: Table S3). Collectively, these findings suggested that intratumoral CD8+ T-cell status may depend more on the characteristics of the tumor than on the general status of patient.

### Intratumoral CD8 + T_predys_^low^ and CD8 + T_dys_^high^ were significantly associated with LNM and worse prognosis

Next, we sought to understand whether the density of intratumoral CD8+ T-cell functional subsets correlated with LNM. The multivariate logistic regression model was adjusted for age (≤ 60 years vs. > 60 years), gender (male vs. female), histological subtype (squamous cell carcinoma vs. adenocarcinoma) and tumor diameter (≤ 3 cm vs. > 3 cm). Density of CD8+ T_total_ in IM [OR = 0.08; 95%CI (0.04–0.16); *P <* 0.001] and TC [OR = 0.06; 95%CI (0.03–0.11); *P <* 0.001], density of CD8+ T_predys_ cells [OR = 0.51; 95%CI (0.29–0.88); *P* = 0.015], and density of CD8+ T_dys_ [OR = 5.80; 95%CI (3.19–10.54); *P <* 0.001] were significantly associated with LNM, independent of the above clinicopathological factors (Fig. 3d). Multicollinearity among the variables was not notable in the multivariate models (the variance inflation factor of each independent variable was less than 10 and the corresponding tolerance was more than 0.1). Collectively, T cells with specific functional states in specific spatial location are critical determinants of LNM.

Subsequently, we assessed the relationship between the density of intratumoral CD8+ T-cell functional subsets and the prognosis of NSCLC. Patients were stratified into low or high groups according to the density of CD8+ T-cell subsets in X-tile 3.6.1 software (Yale university, USA). It was demonstrated that low densities of CD8+ T_total_ in IM and TC, CD8+ T_predys_ in TC and a high density of CD8+ T_dys_ in IM were significantly associated with worse RFS (Additional file 1: Figures S2, S3). After adjusting for clinicopathological factors (including age, gender, histological subtype, tumor diameter and lymph node status), densities of CD8+ T_total_ in IM [HR = 0.57; 95%CI (0.35–0.92); *P* = 0.021], CD8+ T_predys_ in TC [HR = 0.55; 95%CI (0.34–0.89); *P* = 0.014] and CD8+ T_dys_ in IM [HR = 2.49; 95%CI (1.60–4.13); *P* = 0.012] remained significantly associated with RFS (Additional file 1: Figure S3). Given that lymph node status is the primary determinant of recurrence in operable NSCLC, the results of RFS-risk analysis and LNM-risk analysis are broadly consistent.

### Analysis of mean nearest neighbor distance revealed a potentially stronger immunomodulatory network in NSCLC patients with LNM

**Fig. 4:**
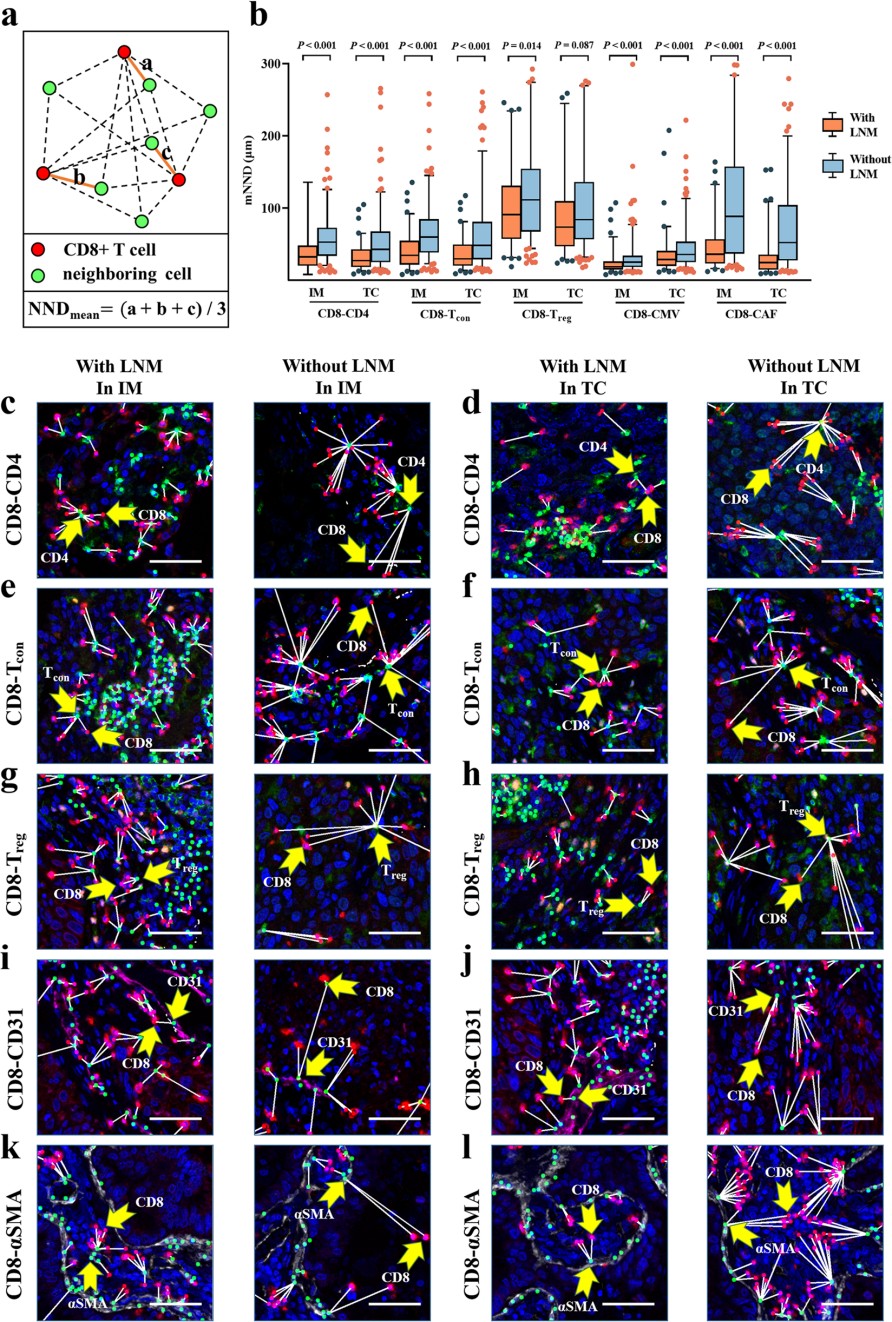
Analysis of the mean nearest neighbor distance from intratumoral CD8 + T cell to neighboring cells. **a** Illustration of the calculation of the mNND. **b** Discrepancies of the mNND from CD8 + T cells to functional subsets between patients with LNM and without LNM. Significance (*P* value) was determined by Mann–Whitney U test. **c**–**l** Representative multiplex immunofluorescence images exhibited the discrepancies between NSCLC patients with and without LNM. Scale bar, 50 mm

Cell–cell interactions are an intrinsic part of cancer immunity, driving the role of immune cells in a specific TME, exemplified by the intricate interplay between CD8+ T cells and neighboring cells. Given our ability to precisely define the coordinates of each cell, we next sought to evaluate the interaction between CD8+ T cells and stromal cells, including CD4+ T cells, CAFs and endothelial cells of CMVs, using the concept of mean nearest neighbor distance (mNND) (Fig. 4a; see Methods).

Firstly, we found that intratumoral CD8+ T cells of patients with LNM presented a shorter mNND to CD4+ T_con_, CD4+ T_reg_, CMVs and CAFs compared with those of patients without LNM (Fig. 4b), implying a potentially stronger immunomodulatory network in patients with LNM, which was illustrated more visually in mIF images (Fig. 4c–l). More importantly, after adjusting for clinicopathological factors (including age, gender, histological subtype, and tumor diameter), these discrepancies remained significantly associated with LNM in multivariate models (Additional file 1: Figure S4). Furthermore, it was demonstrated that shorter mNND (CD8-CD4) in IM, shorter mNND (CD8-T_con_) in IM, shorter mNND (CD8-T_reg_) in IM, shorter mNND (CD8-CAF) in IM and in TC were associated with poor RFS. In multivariate Cox models, shorter mNND (CD8-T_reg_) in IM [HR = 1.72; 95%CI (1.26–2.92); *P* = 0.024] and shorter mNND (CD8-CAF) in IM [HR = 1.57; 95%CI (1.11–2.43); *P* = 0.024] were still significant associated with worse RFS after adjusting for clinicopathological factors (including age, gender, histological subtype, tumor diameter and lymph node status) (Additional file 1: Figures S5, S6).

In addition, mNND was compared among patients grouped by other clinicopathological factors. Generally, with respect to mNND between CD8+ T cells and neighboring cells, there were few significant differences in patients grouped by age and performance status. Interestingly, LUSC, male and moderate-to-severe smoking patients showed shorter mNND between CD8+ T cells and CD4+ T-cell subsets, including CD4+ T_con_ cells (*P <* 0.001) and T_reg_ cells (*P <* 0.001) in IM, which was in contrast to that in TC (*P* = 0.003 for T_con_; *P* = 0.399 for T_reg_). In addition, mNND between CD8 and CD4+ T cell subsets decreased as T stage advanced in IM (*P <* 0.001 for CD4+ T_total_; *P <* 0.001 for CD4+ T_con_; *P* = 0.011 for CD4+ T_reg_) (Additional file 1: Table S5). Collectively, these findings suggested that the degree of interplay between intratumoral CD8+ T cells and neighboring cells may depend more on the characteristics of the tumor than on the general status of patient.

### Analysis of cancer-cell proximity score revealed cancer microvessels and cancer-associated fibroblasts selectively promote CD8 + T-cell exclusion and dysfunction

**Fig. 5:**
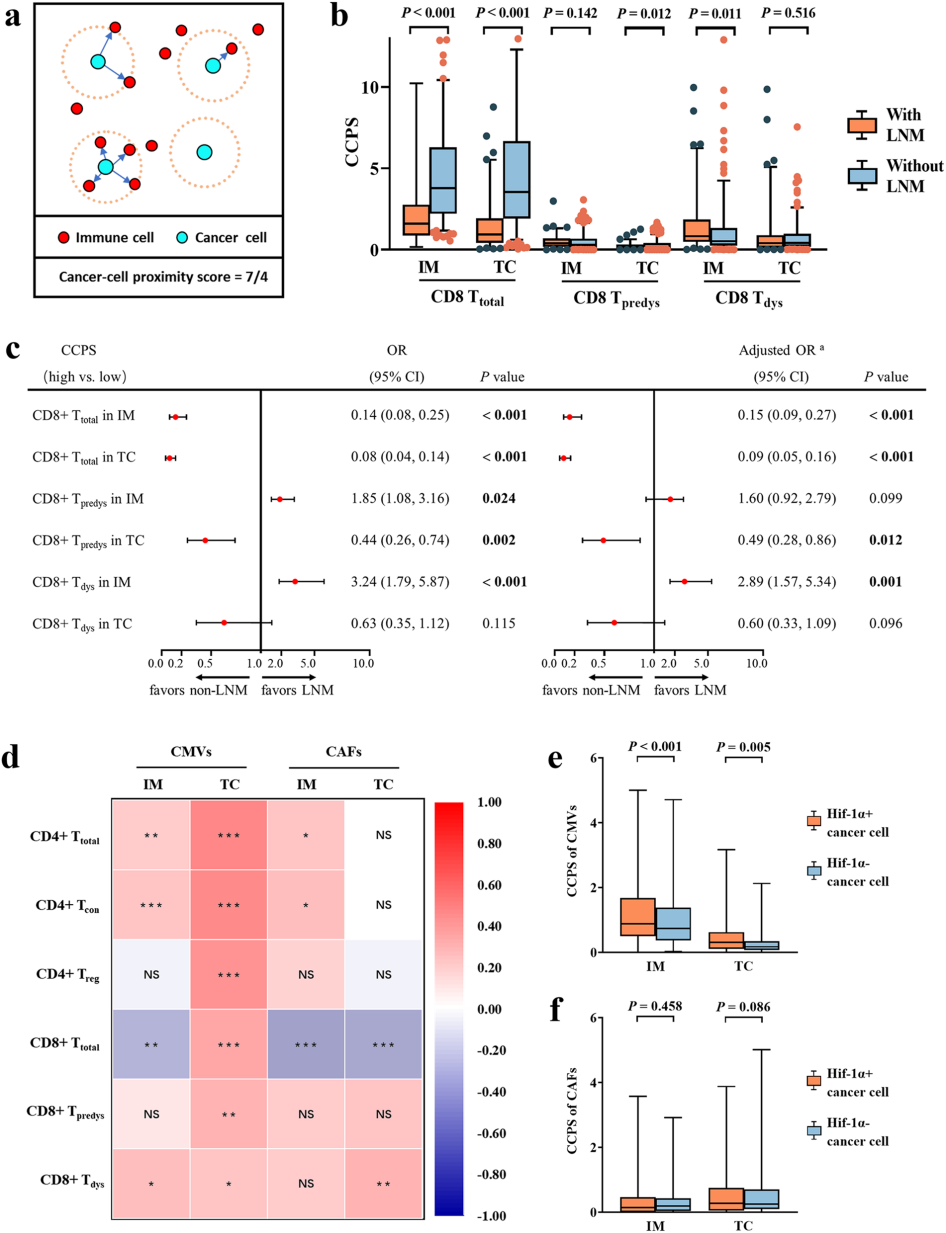
Microvessels and fibroblasts were correlated with exclusion and dysfunction of CD8 + T cells. **a** Illustration of the calculation of the cancer-cell proximity score (CCPS). **b** Discrepancies of the CCPS of CD8 + T-cell functional subsets between patients with LNM and without LNM. **c** Forest plots of univariate and multivariate logistic models demonstrated the relationships between the CCPS of CD8 + T-cell functional subsets and LNM. ^a^ The multivariate logistic model adjusted age, gender, histology subtype and tumor diameter. **d** Correlation heatmap exhibited the relationships between the CCPS of stromal cells and T cells. Significance (*P* value) was determined by Spearman rank correlation analysis. * 0.01 ≤ *P* < 0.05, ** 0.001 ≤ *P* < 0.01, *** *P* < 0.001, NS, no significance. Discrepancies of the proximity of CMVs (**e**), CAFs (**f**) between hypoxic and normoxic cancer cells. Significance (*P* value) was determined by Mann–Whitney U test

To further study the regulatory mechanism of the infiltration of intratumoral CD8+ T cells, we established a comprehensive parameter, cancer-cell proximity score, to recognize immune cells with the ability to interact with cancer cells, incorporating quantitative and spatial information (Fig. 5a).

Similar to cell density, CCPS was firstly compared between patients with and without LNM. Lower CCPS of CD8+ T_total_ cells in both IM (*P* < 0.001) and TC (*P* < 0.001), lower CCPS of CD8+ T_predys_ cells in TC (*P* = 0.012) and higher CCPS of CD8+ T_dys_ cells in IM (*P* = 0.011) (Fig. 5b) were observed in patients with LNM. As for the subgroups of patients with other clinicopathological factors, a lower CCPS of CD8+ T_total_ cells in IM was observed in patients with younger age (*P* = 0.006) and poorer performance status (*P <* 0.001). Compared with LUAD, LUSC showed lower CCPS of CD8+ T_total_ cells (*P* = 0.018) and CD8+ T_predys_ cells (*P <* 0.011) in TC but higher CCPS of CD8+ T_predys_ cells (*P* = 0.021) and CD8+ T_dys_ cells (*P* = 0.002) in IM. Furthermore, with advanced T stage, a lower CCPS of CD8+ T_total_ cells in IM (*P* = 0.002) and TC (*P <* 0.001), lower CCPS of CD8+ T_predys_ cells in TC (*P* = 0.016) and higher CCPS of CD8+ T_dys_ cells in IM (*P* = 0.018) were observed (Additional file 1: Table S6).

Subsequently, univariate and multivariate models were established to examine the relationships between the CCPS of intratumoral CD8+ T-cell subsets and LNM as well as prognosis. The multivariate logistic model, which was adjusted for clinicopathological factors (including age, gender, histological subtype, and tumor diameter) demonstrated that low CCPS of CD8+ T_total_ in IM [OR = 0.15; 95%CI (0.09-0.27); *P* < 0.001] and TC [OR = 0.09; 95%CI (0.05-0.16); *P* < 0.001], low CCPS of CD8+ T_predys_ [OR = 0.49; 95%CI (0.28-0.86); *P* = 0.012], and high CCPS of CD8+ T_dys_ [OR = 2.89; 95%CI (1.57-5.34); *P* = 0.001] were significantly associated with LNM (Fig. 5c). After adjusting for clinicopathological factors (including age, gender, histological subtype, tumor diameter and lymph node status), multivariate Cox models demonstrated that low CCPS of CD8+ T_total_ in IM [HR = 0.54; 95%CI (0.34-0.86); *P* = 0.009] and high CCPS of CD8+ T_dys_ in IM [HR = 1.65; 95%CI (1.14-2.38); *P* = 0.017] maintained significant correlations with poor RFS that also existed in univariate models and survival analysis (Additional file 1: Figures S7, S8).

More importantly, as a spatially limited cell density, the analysis of CCPS provides an opportunity to observe the infiltration barrier obstructing CD8+ T cells from contacting cancer cells. CMVs are transport pipelines for the lymphocytes. However, abnormal CMVs in NSCLC have been proposed to impede T-cell trafficking. Besides, CAFs have been hypothesized to act as barriers to retard lymphocyte infiltration. Therefore, the CCPS of T cell subsets, CMVs and CAFs within the same 30μm radius was determined, and the correlation among them was explored. For CMVs, in IM, we observed a negative correlation between the CCPS of CMVs and CD8+ T_total_, whereas positive correlations were observed between CMVs and CD4+ T_total_. Notably, in TC, the CCPS of CMVs was positively correlated with all T cell subsets detected (Fig. 5d), which indicated that the CMVs in IM but not in TC may act as selective pipelines for T-cell infiltration, impeding the trafficking of CD8+ T cells rather than CD4+ T cells. Interestingly, the CCPS of CMVs was positively correlated with CD8+ T_dys_, suggesting a potential effect of CMVs on promoting CD8+ T-cell dysfunction. Similarly, we investigated the relationship between T cells and CAFs adjacent to the cancer cells. In IM, we observed that the CCPS of CAFs was negatively correlated with CD8+ T_total_ but positively correlated with CD4+ T_total_ and CD4+ T_con_ cells. In TC, the CCPS of CAFs was also negatively correlated with CD8+ T_total_ but more importantly, positively correlated with CD8+ T_dys_ (Fig. 5d). These results indicate that CAFs in NSCLC act not only as a mechanical barrier but can also affect the dysfunction of T cells.

Finally, we sought to understand whether cancer-cell hypoxia could affect CMVs and CAFs barriers. We observed more CMVs around hypoxic cancer cells (pan-CK+/Hif-1α+) than normoxic cancer cells (pan-CK+/Hif-1α−) in both IM and TC, which supported the hypothesis that hypoxia in cancer cells could induce angiogenesis (Fig. 5e), whereas no significant difference was observed hin the analysis of CAFs (Fig. 5f).

## Discussion

In this study, utilizing mIF combined with machine learning-assisted image analysis, we deciphered the specific TME affecting the density and distribution of CD8+T cell functional subpopulations associated with LNM. Our results demonstrated that tumor-infiltrating CD8+ T cells were featured by decreased T_predys_ cells and increased T_dys_ cells, driven by a special immunosuppressive microenvironment, which prompted us to consider whether it is appropriate to give anti-PD-(L)1 perioperative immunotherapy in all PD-L1>1% patients with LNM.

In current clinical practice for patients with operable NSCLC, anti-PD-(L)1 immunotherapy has been floundering due to limited efficacy to activate the anti-tumor effects [30, 31]. In our observation, there are two plausible explanations for the limited efficacy of immunotherapy: First, we found a high proportion of CD8+ T_dys_ in NSCLC patients, which was even worse in patients with LNM; Second, some immunoregulatory barriers, such as CMVs and CAFs, were associated with poor infiltration and dysfunction of CD8+ T cells.

Dysfunctional CD8+ T cells used to be characterized using PD-1 alone in previous studies [17]. Actually, the dysfunction of T cells is a successive process. Some immune inhibitory molecules, such as PD-1 and CD103, are mainly expressed on transitory dysfunctional CD8+ T cells, whereas Tim3 and LAG3 are mainly expressed on terminally dysfunctional CD8+ T cells [13, 16, 19]. Therefore, we present a more exact landscape of T cells in NSCLC. Firstly, we found more CD8+ T cells instead of CD4+ T cells in IM than in TC. More importantly, there were more CD8+ T_dys_ in the IM, which may account for the inconsistent prognostic results of CD8+ T cells in previous investigations [32–35]. Moreover, in the multivariate models, our results demonstrated that a low density of CD8+ Tpredys in TC and a high density of CD8+ Tdys in IM were significantly associated with LNM and poor RFS. A previous study found that NSCLC patients with LNM harboring more PD-1+ CD8+ cells, and more PD-1+ CD8+ cells existed in the IM [17]. Using single-cell sequencing, Guo et al. demonstrated that a high ratio of pre-exhausted to exhausted T cells correlated with better prognosis in treatment-naive lung adenocarcinoma [19]. More importantly, the function of dysfunctional CD8+ T cells were considered not able to be recovered by PD-(L)1 inhibitors [36]. Notably, adaptive resistance to PD-1 inhibitors is associated with the upregulation of Tim3 [37]. Collectively, the dysfunction of T cells represents a distinct status of T-cell differentiation with considerable clinical relevance. However, we still understand little about where, when and how to initiate or execute a dysfunction program.

Intercellular interactions play an important role in the regulation of immune cell function. We revealed a stronger CD8+ T-cell immunomodulatory network in NSCLC patients with LNM than in those without LNM. Specifically, shorter mNNDs between intratumoral CD8+ T cells and CD4+ T_con_, CD4+ T_reg_, CMVs and CAFs were observed in lymph node positive patients and were associated with worse RPS. A previous study showed that stronger CD8-T_reg_ proximity was associated with poor OS in NSCLC [11], while another study found that a higher density of CD3+CD8+ cells neighboring CD3+CD8- cells was associated with better prognosis, despite the small sample size (n=20) [10]. Furthermore, using single-cell transcriptome sequencing, Li et al. found that, in melanoma, both T_con_ cells and T_reg_ cells displayed levels of proliferation comparable to those observed in dysfunctional CD8+ T cells [38]. Overall, although we provided novel insights, the interaction between CD4+ and CD8+ T-cell subsets remains ambiguous and requires more efforts.

The vasculature is pivotal for the transportation of immune cells to the target tissues. Our results showed that CMVs were correlated with increased proximity of all T cell subsets in the TC but were correlated with decreased proximity of CD8+ T_total_ in the IM, indicating that CMVs may be selective for T cells in the IM, reducing the infiltration of CD8+ T cells rather than CD4+ T cells. More importantly, we found that CMVs were associated with high proximity of CD8+ T_dys_ in the IM. Previous studies have demonstrated that cancer vasculature could impede T-cell trafficking through endothelial cell anergy via the downregulation of adhesion molecules [20] and through the establishment of a death barrier via the upregulation of apoptotic ligands [21]. Notably, CD4+ T cells in human primary lung tumors are Th2 skewed [39], and Th2 cells express a higher level of anti-apoptotic signals [40, 41]. Our findings strengthen the hypotheses of tumor vasculature as a “selective barrier” and indicate that there might be additional mechanisms for tumor vasculature to restrict T cells. However, further explorations are required to confirm our results. CAFs are another important stromal component of TME. A recent study identified two types of CAF subsets, MYH11+ αSMA+ CAF and FAP+ αSMA+ CAF, which contribute to T-cell exclusion and could restrict T cells contact with cancer cells [22]. Likewise, our results demonstrated that αSMA+ CAFs were negatively correlated with cancer-proximal CD8+ T_total_ cells in both IM and TC, strengthening the evidence of the “CAF barrier” in lung cancer. Of note, we also observed that CAFs were positively correlated with CD4+ T_total_ cells and CD4+ T_con_ in IM, and CD8+ T_dys_ in TC, which indicated that CAFs might be more than a unselective mechanical barrier. Lakins et al. found that the upregulation of PD-1/PD-L2 and FAS/FASL in T cells/CAFs, respectively, drove the deletion and dysfunction of tumor-specific T cells [42], which provided rationality to our findings.

Collectively, our study revealed that NSCLC patients with LNM were characterized by more dysfunctional intratumoral-infiltrating CD8+ T cells and a more immunosuppressive TME impeding CD8+ T cells. These findings indicated that anti-PD-(L)1 immunotherapy may not work as well in patients with immunosuppressive microenvironment until these adverse factors are eliminated. A combination strategy is a prospective orientation in facilitating immunotherapy. Radiotherapy, which can suppress CAFs and increase the infiltration of T cells, is therefore considered a wonderful partner for immunotherapy [43]. Antiangiogenic agents at mild doses can induce the normalization of tumor vessels and some combination schemes are under clinical trials [44]. More importantly, next-generation immunotherapies, such as CAR-T, in combination with other therapies could improve the infiltration of the modified immune cells or reverse their dysfunction, which may be the key to improving their efficacy [45]. Given that the primary tumor may be an antigen source for activating and expanding tumor-specific T cells and systemic surveillance of micrometastases, neoadjuvant immunotherapy is promising [46]. Unfortunately, some patients may miss the opportunity for neoadjuvant immunotherapy due to occult LNM (LNM is negative in preoperative evaluation but confirmed as positive by postoperative pathology) [4]. Given a high risk of LNM and recurrence, future studies and clinical practice should give more concern over the patients with the above characteristics, requiring adequate clinical assessments and treatments to improve prognosis, such as combination therapy or close follow-up.

Some limitations of this study have to be acknowledged. First, excluding other pathological types, such as large cell lung cancer, from our cohort may restrain the utilization of our findings in a prevalent NSCLC cohort. Second, although rarely, it needs to be known that CD4 and CD8 are also slightly expressed on non-T cells, such as a few macrophages and dendritic cells [47, 48]. It is uncertain whether the setting of a positive threshold can eliminate this effect during the image analysis. Lastly, the lack of functional tests on T cells and comprehensive analysis of other relevant immune cells may reduce the embodiment of the complete immune microenvironment. Therefore, further independent studies are warranted to confirm our findings.

Despite these limitations, our study has several explicit advantages. Firstly, our customized mIF methods allowed for the synchronous detection of various protein markers in single tissue slide, providing a thorough perspective of the TME. Secondly, our tissue-based analysis revealed an unreported spatial interaction network of CD8+ T cells that could not be uncovered using dissociative techniques such as flow cytometry or single cell RNA sequencing. Meanwhile, quantitative digital analysis emphasizes the superiority of computer-assisted quantitation over the visual counting of positive cells by pathologists alone. Lastly, our exploration of the role of stromal cells in T cells, reported as the correlation between the CCPS of stromal cells and T cells, in contrast to most prior studies that provide only cell quantity, can better corroborate the hypothesis of stromal barriers based on a rational parameter combining quantity and spatial structure.

In summary, we performed a comprehensive decipherment of the spatial structure of intratumoral CD8+ T cells in NSCLC patients with LNM based on mIF images, including special spatial distribution of CD8+ T-cell functional subsets and spatial interplay between CD8+ T cells and their neighboring cells, which revealed the complexity of TME with significant implications for facilitating precise immunotherapy.

## Supplementary Information

**Additional file 1: Figure S1.** Flowchart of the patient enrollment in this study**. Figure S2.** Survival analysis of the cell densities of intratumoral-infiltrating CD8 + T-cell subsets in patients with NSCLC. **Figure S3.** The risk-correlation analysis of recurrence-free survival based on the density of CD8 + T-cell subsets in NSCLC. **Figure S4.** The risk-correlation analysis of lymph node metastases based on the mean nearest neighbor distance between CD8 + T cells and neighboring cells concerned in NSCLC. **Figure S5.** Survival analysis of the mean nearest neighbor distances between intratumoral CD8 + T cells and neighboring cells in patients with NSCLC. **Figure S6.** The risk-correlation analysis of recurrence-free survival based on the mean nearest neighbor distance between CD8 + T cells and neighboring cells concerned in NSCLC. **Figure S7.** Survival analysis of the cancer-cell proximity scores between intratumoral CD8 + T cells and neighboring cells in patients with NSCLC. **Figure S8.** The risk-correlation analysis of recurrence-free survival based on the cancer-cell proximity score of CD8 + T-cell functional subsets in NSCLC. **Table S1.** Information of primary antibodies used in the multiplex immunofluorescence. **Table S2.** Scheme of cell phenotypes in multiplex immunofluorescence. **Table S3.** The discrepancy of the density of CD8 + T-cell functional subsets among the NSCLC patients grouped by clinicopathological factors. **Table S4.** The discrepancy of the density of compartment-special CD8 + T-cell functional subsets among the NSCLC patients grouped by clinicopathological factors. **Table S5.** The discrepancy of the mean nearest distance between CD8 + T cells and neighboring cells among NSCLC patients grouped by clinicopathological factors. **Table S5.** The discrepancy of the cancer-cell proximity score of CD8 + T-cell functional subsets among the NSCLC patients grouped by clinicopathological factors.

**Additional file 2.** Overview of stained sections from all tissue microarray before and after fluorescence imaging.

## Data Availability

The datasets used and/or analyzed during the current study are available from the corresponding author on reasonable request.

## Abbreviations

NSCLC: Non-small cell lung cancer
LNM: Lymph node metastases
IM: Invasive margin
TC: Tumor center
mNND: Mean nearest neighbor distance
CCPS: Cancer-cell proximity score
CMV: Cancer microvessel
CAF: Cancer-associated fibroblast
mIF: Multiplex immunofluorescence
TMA: Tissue microarray
FFPE: Formalin-fixed, paraffin-embedded
TSA: Tyramide signal amplification
T_total_: Total T cell
T_con_: Conventional T cell
T_reg_: Regulatory T cell
T_cyto_: Cytotoxic T cell
T_predys_: Predysfunctional T cell
T_dys_: Dysfunctional T cell
RFS: Recurrence-free survival
ROI: Region of interest
IQR: Interquartile range
LUSC: Lung squamous cell carcinoma
LUAD: Lung adenocarcinoma
OR: Odds ratio
CI: Confidence interval
HR: Hazard ratio
