# Supplementary material for: Functional status and spatial architecture of tumor-infiltrating CD8+ T cells are associated with lymph node metastases in non-small cell lung cancer
Source: J Transl Med. 2023 May 12;21:320. doi: 10.1186/s12967-023-04154-y (PMC10182600; doi:10.1186/s12967-023-04154-y)
Supplement: Supplementary file 1 — Additional file 1: Figure S1. Flowchart of the patient enrollment in this study. Figure S2. Survival analysis of the cell densities of intratumoral-infiltrating CD8 + T-cell subsets in patients with NSCLC. Figure S3. The risk-correlation analysis of recurrence-free survival based on the density of CD8 + T-cell subsets in NSCLC. Figure S4. The risk-correlation analysis of lymph node metastases based on the mean nearest neighbor distance between CD8 + T cells and neighboring cells concerned in NSCLC. Figure S5. Survival analysis of the mean nearest neighbor distances between intratumoral CD8 + T cells and neighboring cells in patients with NSCLC. Figure S6. The risk-correlation analysis of recurrence-free survival based on the mean nearest neighbor distance between CD8 + T cells and neighboring cells concerned in NSCLC. Figure S7. Survival analysis of the cancer-cell proximity scores between intratumoral CD8 + T cells and neighboring cells in patients with NSCLC. Figure S8. The risk-correlation analysis of recurrence-free survival based on the cancer-cell proximity score of CD8 + T-cell functional subsets in NSCLC. Table S1. Information of primary antibodies used in the multiplex immunofluorescence. Table S2. Scheme of cell phenotypes in multiplex immunofluorescence. Table S3. The discrepancy of the density of CD8 + T-cell functional subsets among the NSCLC patients grouped by clinicopathological factors. Table S4. The discrepancy of the density of compartment-special CD8 + T-cell functional subsets among the NSCLC patients grouped by clinicopathological factors. Table S5. The discrepancy of the mean nearest distance between CD8 + T cells and neighboring cells among NSCLC patients grouped by clinicopathological factors. Table S5. The discrepancy of the cancer-cell proximity score of CD8 + T-cell functional subsets among the NSCLC patients grouped by clinicopathological factors. [file 12967_2023_4154_MOESM1_ESM.docx]

Additional file-1

Functional status and spatial architecture of tumor-infiltrating CD8+ T cells are associated with lymph node metastases in non-small cell lung cancer

Guanqun Yang^#^, Siqi Cai^#^, Mengyu Hu, Chaozhuo Li, Liying Yang, Wei Zhang, Jujie Sun, Fenghao Sun, Ligang Xing, Xiaorong Sun^*^


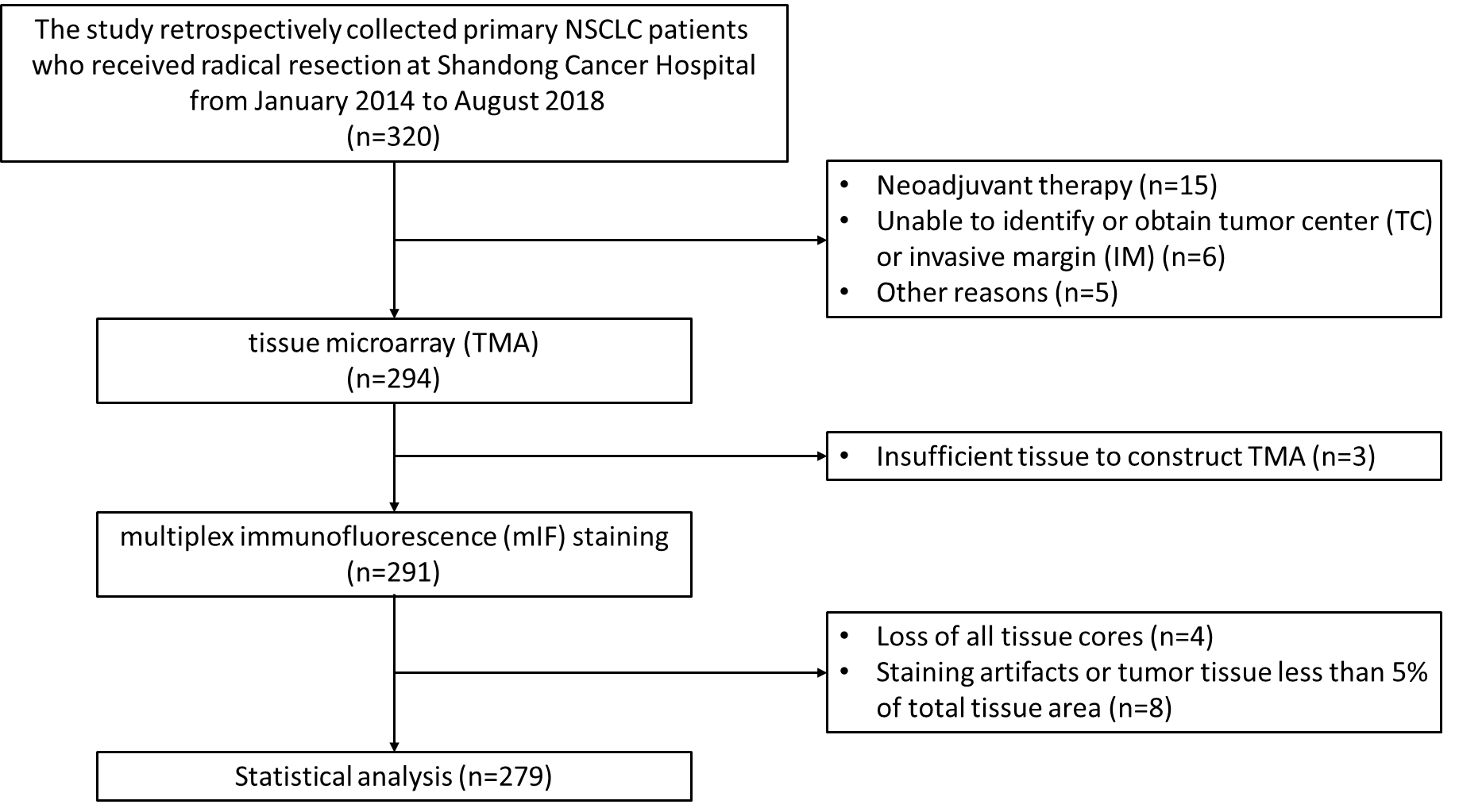


**Additional Figure S1. Flowchart of the patient population in this study.**


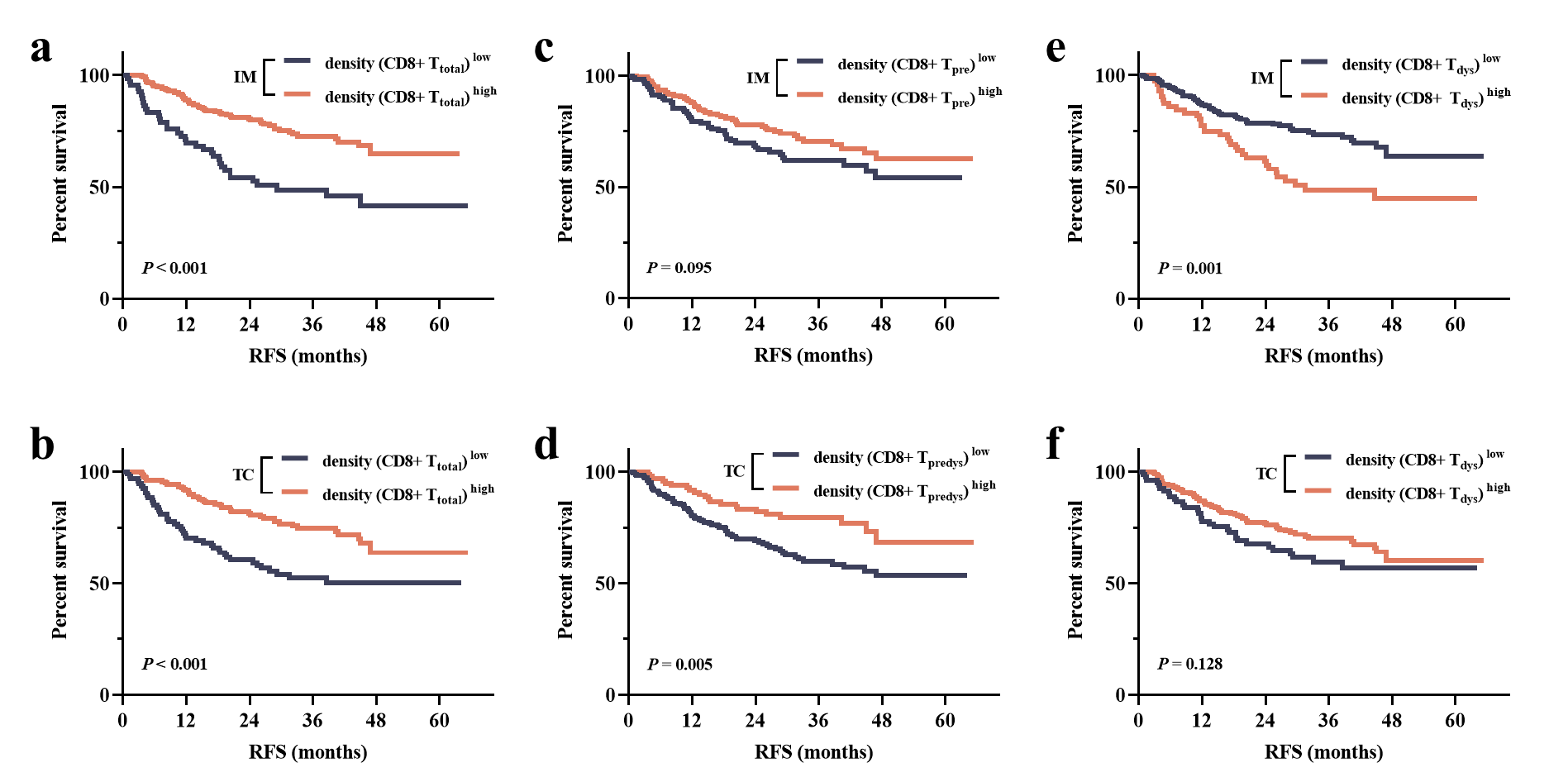


**Additional Figure S2. Survival analysis of the cell densities of intratumoral-infiltrating CD8+ T-cell subsets in patients with NSCLC. (a-f)** Kaplan-Meier survival analysis of intratumoral CD8+ T-cell subsets’ density-related parameters in patients with NSCLC, including CD8+T_total_ in IM **(a)** and in TC **(b)**, CD8+T_predys_ in IM **(c)** and in TC **(d)**, CD8+T_dys_ in IM **(e)** and in TC **(f)**. Significance (*P* value) was determined using Log-rank test. IM, invasive margin; TC, tumor center; RFS, recurrence-free survival.


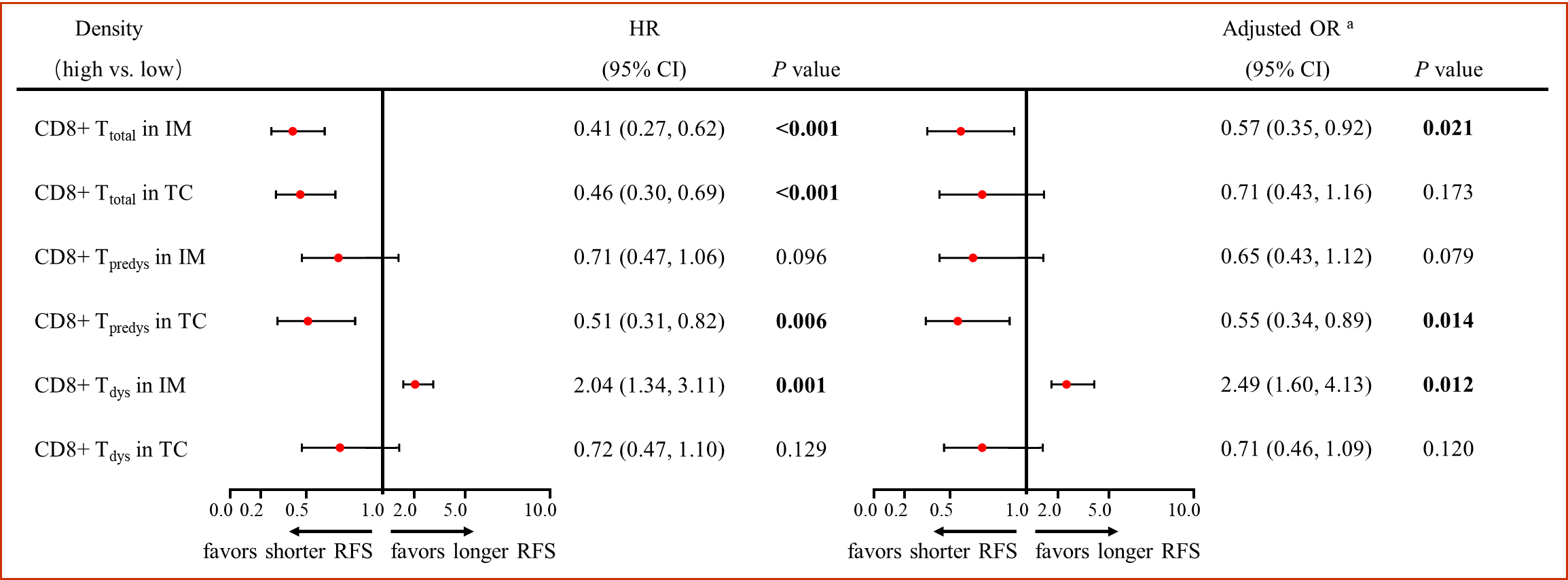


**Additional Figure S3. The risk-correlation analysis of recurrence-free survival based on the density of CD8+ T-cell subsets in NSCLC.** ^a^ The multivariate Cox regression model adjusted age (≤ 60 years vs. ˃60 years), gender (male vs. female), histological subtype (squamous cell carcinoma vs. adenocarcinoma), tumor diameter (≤ 3 cm vs. ˃3 cm) and status of lymph node (positive vs. negative). A backward elimination with a threshold of *P* = 0.05 was used to select variables in the final models. Abbreviations: CI, confidence interval; HR, hazard ratio; IM, invasive margin; TC, tumor center; T_predys_, pre-dysfunctional CD8+ T cell; T_dys_, dysfunctional CD8+ T cell.


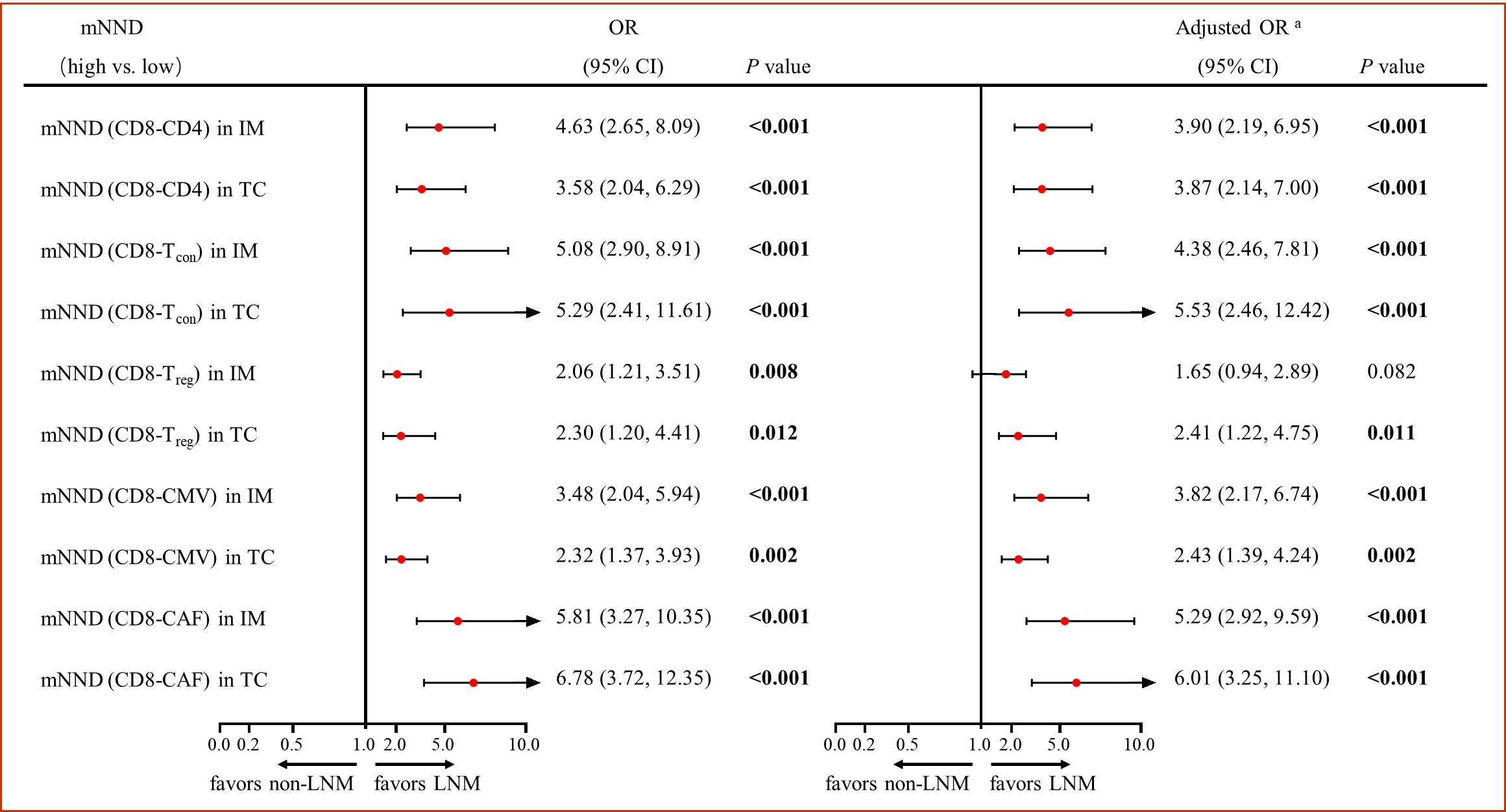


**Additional Figure S4. The risk-correlation analysis of lymph node metastasis based on the mean nearest neighbor distance between CD8+ T cells and neighboring cells concerned in NSCLC.** ^a^ The multivariate Cox regression model adjusted age (≤ 60 years vs. ˃60 years), gender (male vs. female), histological subtype (squamous cell carcinoma vs. adenocarcinoma) and tumor diameter (≤ 3 cm vs. ˃3 cm). A backward elimination with a threshold of *P* = 0.05 was used to select variables in the final models. Abbreviations: CI, confidence interval; OR, odds ratio; IM, invasive margin; TC, tumor center; T_con_, conventional CD4+ T cell; T_reg_, regulatory CD4+ T cell; CMV, cancer microvessel; CAF, cancer-associated fibroblast.


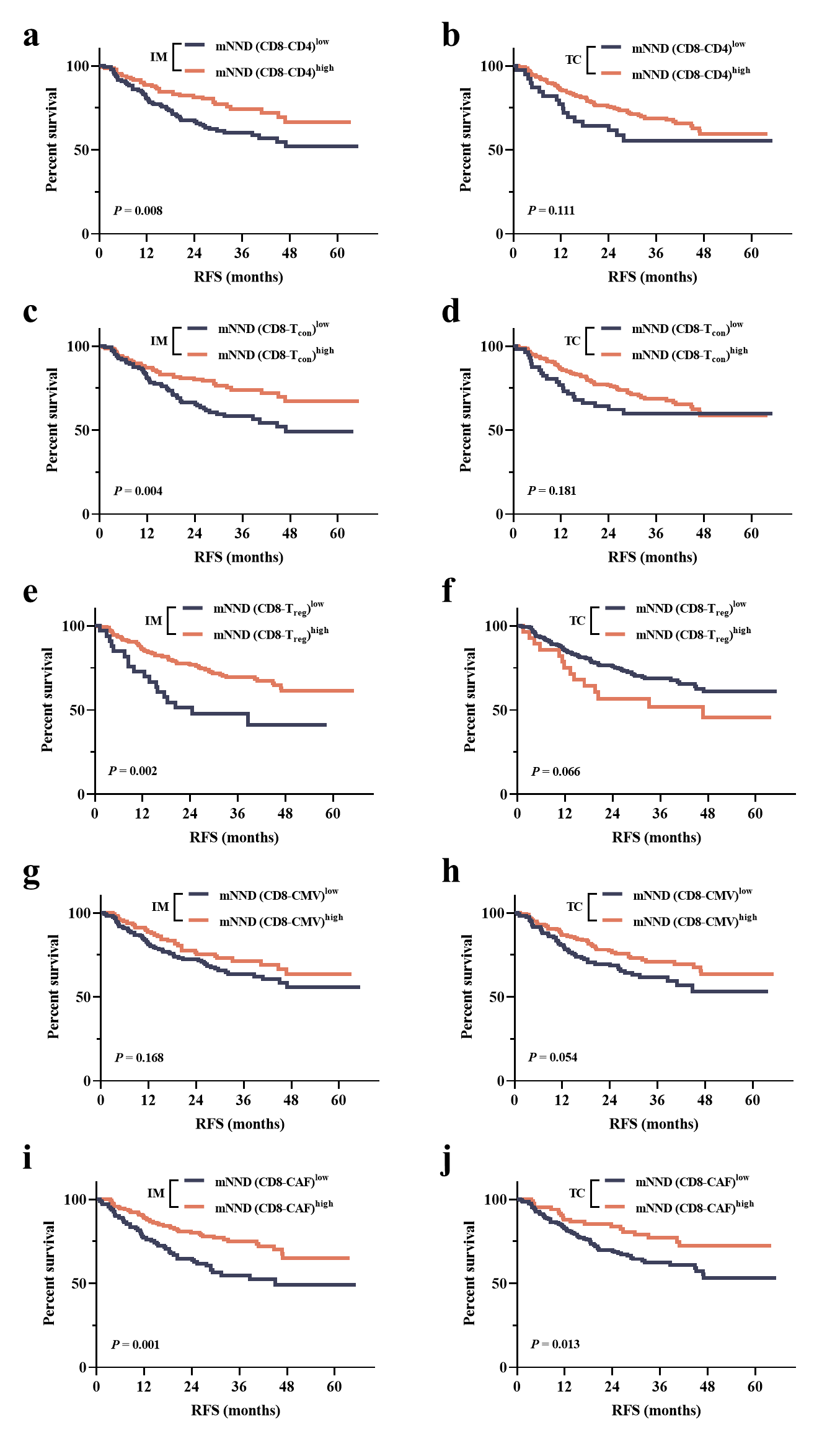


**Additional Figure S5. Survival analysis of the mean nearest neighbor distances between intratumoral CD8+ T cells and neighboring cells in patients with NSCLC. (a-j)** Kaplan-Meier survival analysis of intratumoral CD8+ T cells’ mNND-related parameters in patients with NSCLC, including mNND (CD8-CD4) in IM **(a)** and in TC **(b)**, mNND (CD8-T_con_) in IM **(c)** and in TC **(d)**, mNND (CD8-T_reg_) in IM **(e)** and in TC **(f)**, mNND (CD8-CMV) in IM **(g)** and in TC **(h)**, mNND (CD8-CAF) in IM **(i)** and in TC **(j)**. Significance (*P* value) was determined using Log-rank test. mNND, mean nearest neighbor distance; IM, invasive margin; TC, tumor center; T_con_, conventional CD4+ T cell; T_reg_, regulatory CD4+ T cell; CMV, cancer microvessel; CAF, cancer-associated fibroblast; RFS, recurrence-free survival.


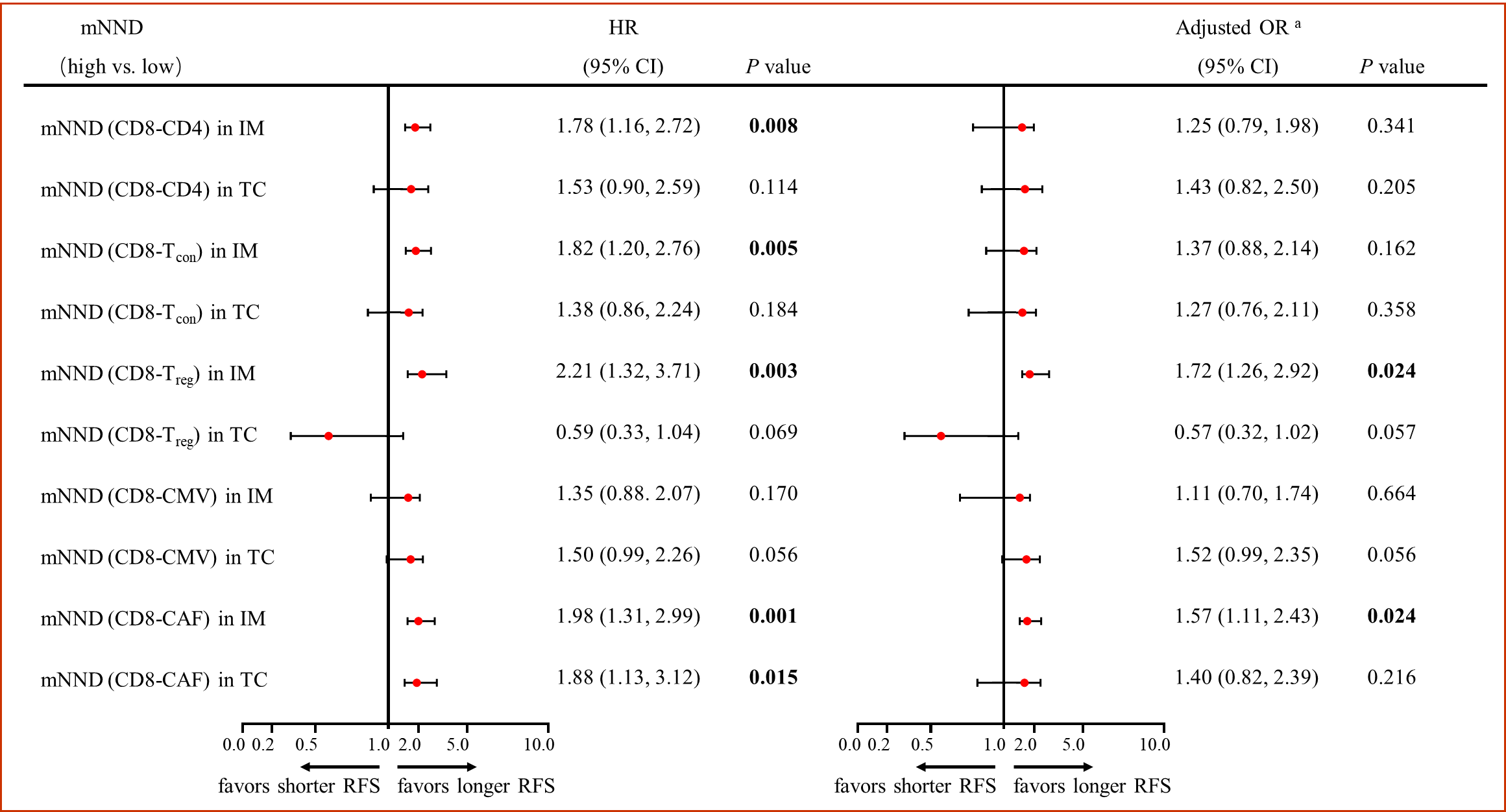


**Additional Figure S6. The risk-correlation analysis of recurrence-free survival based on the mean nearest neighbor distance between CD8+ T cells and neighboring cells concerned in NSCLC.** ^a^ The multivariate Cox regression model adjusted age (≤ 60 years vs. ˃60 years), gender (male vs. female), histological subtype (squamous cell carcinoma vs. adenocarcinoma), tumor diameter (≤ 3 cm vs. ˃3 cm) and status of lymph node (positive vs. negative). A backward elimination with a threshold of *P* = 0.05 was used to select variables in the final models. Abbreviations: CI, confidence interval; HR, hazard ratio; IM, invasive margin; TC, tumor center; T_con_, conventional CD4+ T cell; T_reg_, regulatory CD4+ T cell; CMV, cancer microvessel; CAF, cancer-associated fibroblast.


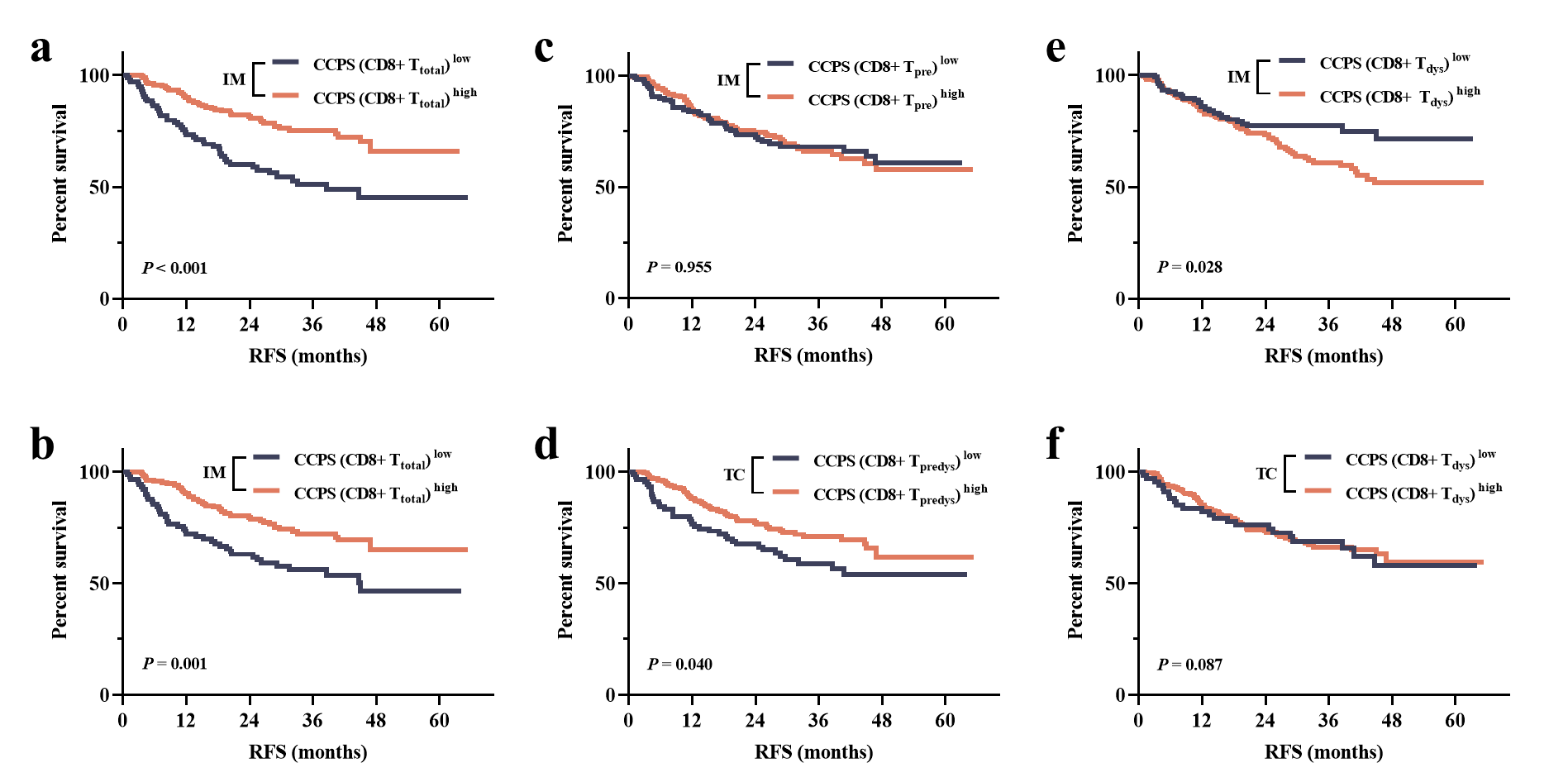


**Additional Figure S7. Survival analysis of the cancer-cell proximity scores between intratumoral CD8+ T cells and neighboring cells in patients with NSCLC. (a-f)** Kaplan-Meier survival analysis of intratumoral CD8+ T cells’ CCPS-related parameters in patients with NSCLC, including CD8+T_total_ in IM **(a)** and in TC **(b)**, CD8+T_predys_ in IM **(c)** and in TC **(d)**, CD8+T_dys_ in IM **(e)** and in TC **(f)**. Significance (*P* value) was determined using Log-rank test. IM, invasive margin; TC, tumor center; RFS, recurrence-free survival.


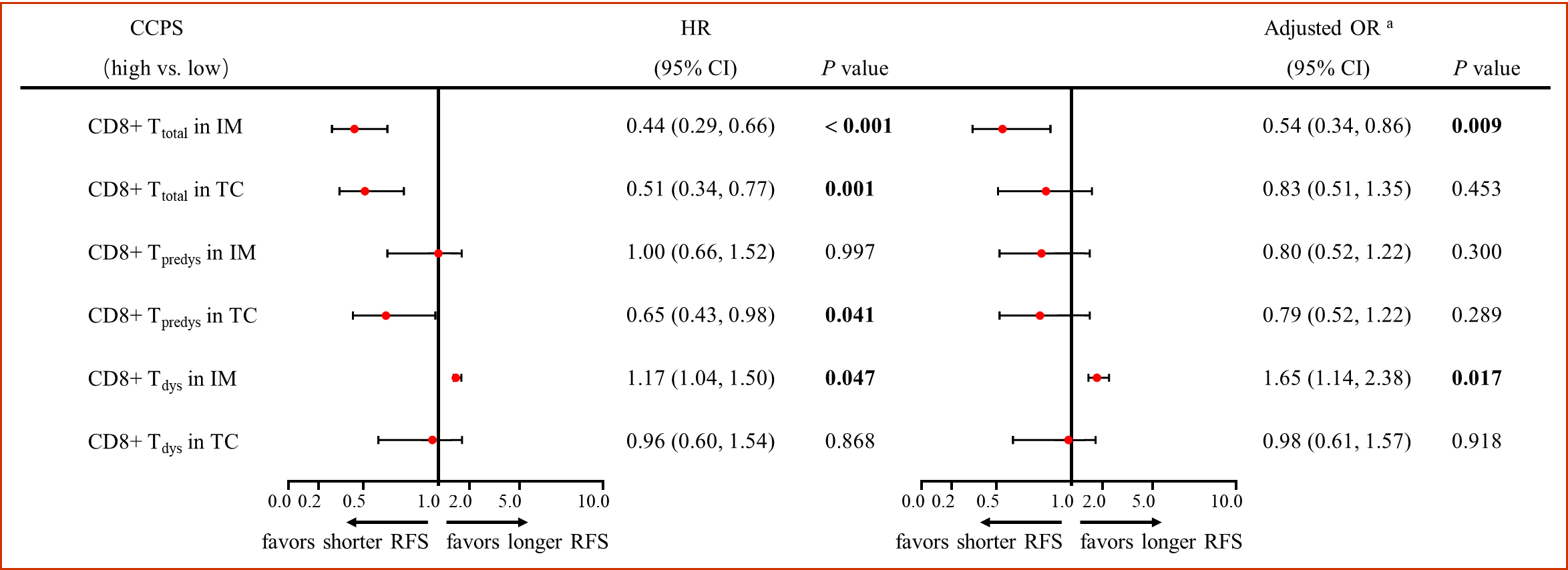


**Additional Figure S8. The risk-correlation analysis of recurrence-free survival based on the cancer-cell proximity score of CD8+ T-cell functional subsets in NSCLC.** ^a^ The multivariate Cox regression model adjusted age (≤ 60 years vs. ˃60 years), gender (male vs. female), histological subtype (squamous cell carcinoma vs. adenocarcinoma), tumor diameter (≤ 3 cm vs. ˃3 cm) and status of lymph node (positive vs. negative). A backward elimination with a threshold of *P* = 0.05 was used to select variables in the final models. Abbreviations: CI, confidence interval; HR, hazard ratio; IM, invasive margin; TC, tumor center; T_predys_, pre-dysfunctional CD8+ T cell; T_dys_, dysfunctional CD8+ T cell.

| **Additional *Table S1. Information of primary antibodies used in the multiplex immunofluorescence test*** | | | |
| --- | --- | --- | --- |
| **Reagent** | **Provider** | **Identifier** | **Concentration** |
| pan-CK | ZSGB-BIO | ZM-0069 | 1:200 |
| CD4 | ZSGB-BIO | ZM-0418 | 1:1 |
| CD8 | Abcam | ab199016 | 1:500 |
| Foxp3 | Abcam | ab20034 | 1:100 |
| CD31 | Abcam | ab76533 | 1:100 |
| ɑSMA | Abcam | ab7817 | 1:200 |
| Hif-1ɑ | Abcam | ab51608 | 1:100 |
| CD103 | Abcam | ab224202 | 1:200 |
| GZMB | Abcam | ab255598 | 1:2000 |
| Tim3 | Cell Signaling Technology | 45208S | 1:100 |
| PD-1 | ZSGB-BIO | ZM-0381 | 1:1 |

| **Additional *Table S2. Scheme of cell phenotypes in multiplex immunofluorescence detection.*** | | |
| --- | --- | --- |
| **Cell set** | **Cell subset** | ***Marker*s** |
| Cancer cell | N/A | pan-CK+ |
| CD8+ T cell | Total CD8+ T cell (CD8+ T_total_) | CD8+ |
|  | Cytotoxic CD8+ T cell (CD8+ T_cyto_) | CD8+/PD1-/CD103-/Tim3-/GZMB+ |
|  | Predysfunctional CD8+ T cell (CD8+ T_predys_) | CD8+/PD1+/CD103+/Tim3-/GZMB- |
|  |  | CD8+/PD1+/CD103-/Tim3-/GZMB- |
|  |  | CD8+/PD1-/CD103+/Tim3-/GZMB- |
|  | Dysfunctional CD8+ T cell (CD8+ T_dys_) | CD8+/Tim3+ |
| CD4+ T cell | Total CD4+ T cell (CD4+ T_total_) | CD4+ |
|  | Conventional CD4+ T cell (CD4+ T_con_) | CD4+/Foxp3- |
|  | Regulatory CD4+ T cell (CD4+ T_reg_) | CD4+/Foxp3+ |
| Stromal component | Cancer microvessel (CMV) | CD31+ |
|  | Cancer-associated fibroblast (CAF) | αSMA+ |
| Hypoxia indicator | Hypoxic cancer cell | pan-CK+/Hif-1α+ |
| N/A, not available | | |

| ***Additional Table S3. The discrepancy of the density of CD8+ T-cell functional subsets among NSCLC patients grouped by clinicopathological factors*** | | | | | | |
| --- | --- | --- | --- | --- | --- | --- |
| **Cell density** | **Age**  **(≤ 60y vs. >60y)** | **Gender**  **(female vs. male)** | **Smoking index**  **(<400 vs. ≥400)** | **ECOG PS**  **(>1 vs. ≤1)** | **Histological subtype**  **(LUAD vs. LUSC)** | **T Stage**  **(T1 vs T2 vs. T3 + T4)** |
| CD8+ T_total_ in IM | ***P* = 0.012**  (103.04 vs. 121.21) | *P* = 0.099  (98.63 vs. 116.53) | *P* = 0.165  (107.52 vs. 117.04) | ***P* = 0.002**  (88.65 vs. 120.45) | *P* = 0.668  (116.85 vs. 104.78) | ***P* ˂ 0.001**  (135.67 vs. 107.08 vs. 74.65) |
| CD8+ T_total_ in TC | ***P* = 0.030**  (77.36 vs. 108.47) | *P* = 0.556  (103.52 vs. 97.12) | *P* = 0.781  (106.13 vs. 88.45) | ***P* = 0.025**  (72.89 vs. 106.48) | *P* = 0.053  (108.81 vs. 73.35) | ***P* ˂ 0.001**  (122.14 vs. 85.50 vs. 34.86) |
| CD8+ T_predys_ in IM | *P* = 0.680  (15.70 vs. 19.87) | *P* = 0.143  (17.73 vs. 19.02) | ***P* = 0.002**  (15.01 vs.23.34) | *P* = 0.159  (15.97 vs. 19.84) | ***P* = 0.007**  (15.93 vs. 22.90) | *P* = 0.465  (17.59 vs. 18.59 vs. 24.35) |
| CD8+ T_predys_ in TC | *P* = 0.212  (8.13 vs. 10.46) | ***P* = 0.003**  (11.01 vs. 7.78) | ***P* = 0.030**  (10.32 vs. 7.74) | *P* = 0.306  (8.42 vs. 9.62) | ***P* = 0.001**  (11.17 vs. 6.05) | ***P* = 0.010**  (10.55 vs. 9.53 vs. 3.79) |
| CD8+ T_dys_ in IM | *P* = 0.886  (44.62 vs. 43.55) | *P* = 0.149  (34.78 vs. 50.69) | *P* = 0.249  (40.09 vs.51.03) | *P* = 0.079  (54.06 vs. 40.94) | ***P* ˂ 0.001**  (34.69 vs. 74.44) | ***P* ˂ 0.001**  (34.69 vs. 41.36 vs. 102.74) |
| CD8+ T_dys_ in TC | *P* = 0.959  (21.91 vs. 21.43) | *P* = 0.415  (25.85 vs. 20.37) | *P* = 0.474  (23.40 vs. 20.69) | *P* = 0.856  (20.54 vs. 21.88) | *P* = 0.130  (25.53 vs. 19.29) | *P* = 0.226  (25.30 vs.20.38 vs. 17.31) |

| ***Additional Table S4. The discrepancy of the density of compartment-special CD8+ T-cell functional subsets among NSCLC patients grouped by clinicopathological factors*** | | | | | | | |
| --- | --- | --- | --- | --- | --- | --- | --- |
| **Compartment** | **Cell density** | **Age**  **(≤ 60y vs. >60y)** | **Gender**  **(female vs. male)** | **Smoking index**  **(<400 vs. ≥400)** | **ECOG PS**  **(>1 vs. ≤1)** | **Histological subtype**  **(LUAD vs. LUSC)** | **T Stage**  **(T1 vs T2 vs. T3 + T4)** |
| Epithelial | CD8+ T_total_ | ***P* = 0.047**  (75.96 vs. 105.76) | *P* = 0.483  (104.38 vs. 77.46) | *P* = 0.784  (96.94 vs. 75.57) | ***P* = 0.027**  (60.52 vs. 97.93) | *P* = 0.194  (94.14 vs. 75.37) | ***P* ˂ 0.001**  (123.61 vs. 80.73 vs. 33.25) |
|  | CD8+ T_predys_ | *P* = 0.877  (1.54 vs. 1.80) | *P* = 0.237  (2.07 vs. 1.56) | *P* = 0.160  (1.94 vs. 1.53) | *P* = 0.760  (1.64 vs. 1.74) | *P* = 0.096  (1.92 vs. 1.50) | *P* = 0.100  (2.21 vs. 1.64 vs. 1.11) |
|  | CD8+ T_dys_ | *P* = 0.875  (6.65 vs. 7.08) | *P* = 0.367  (6.40 vs. 7.01) | *P* = 0.707  (6.70 vs. 7.08) | *P* = 0.876  (6.81 vs. 6.81) | *P* = 0.933  (6.88 vs. 6.79) | *P* = 0.268  (8.71 vs. 5.67 vs. 7.22) |
| Stromal | CD8+ T_total_ | ***P* = 0.034**  (77.02 vs 101.09) | *P* = 0.990  (90.67 vs. 92.45) | *P* = 0.521  (101.09 vs. 85.05) | ***P* = 0.027**  (69.51 vs. 96.79) | *P* = 0.099  (99.88 vs. 77.73) | ***P* ˂ 0.001**  (114.97 vs. 83.85 vs. 43.96) |
|  | CD8+ T_predys_ | *P* = 0.614  (18.45 vs. 18.49) | ***P* = 0.023**  (19.57 vs. 17.11) | *P* = 0.067  (19.86 vs. 16.29) | *P* = 0.270  (15.21 vs. 20.46) | ***P* = 0.011**  (21.40 vs. 14.32) | ***P* = 0.008**  (24.12 vs. 17.80 vs. 6.90) |
|  | CD8+ T_dys_ | *P* = 0.593  (42.14 vs. 41.39) | *P* = 0.913  (49.59 vs. 38.72) | *P* = 0.899  (45.07 vs. 39.52) | *P* = 0.501  (40.70 vs. 43.72) | *P* = 0.984  (45.07 vs. 37.33) | *P* = 0.233  (53.28 vs. 37.89 vs. 51.49) |

| ***Additional Table S5. The discrepancy of the mean nearest distance between CD8+ T cells and neighboring cells among NSCLC patients grouped by clinicopathological factors*** | | | | | | |
| --- | --- | --- | --- | --- | --- | --- |
| **mNND** | **Age**  **(≤ 60y vs. >60y)** | **Gender**  **(female vs. male)** | **Smoking index**  **(<400 vs. ≥400)** | **ECOG PS**  **(>1 vs. ≤1)** | **Histological subtype**  **(LUAD vs. LUSC)** | **T Stage**  **(T1 vs T2 vs. T3 + T4)** |
| CD8-CD4 in IM | *P* = 0.694  (42.99 vs. 47.49) | ***P* = 0.034**  (53.82 vs. 43.79) | ***P* = 0.006**  (47.67 vs. 40.78) | *P* = 0.554  (43.79 vs. 45.94) | ***P* ˂ 0.001**  (52.72 vs. 38.59) | ***P* ˂ 0.001**  (55.01 vs. 43.85 vs. 29.87) |
| CD8-CD4 in TC | *P* = 0.251  (32.02 vs. 39.75) | ***P* ˂ 0.001**  (26.96 vs. 41.94) | ***P* = 0.007**  (31.05 vs. 43.38) | *P* = 0.461  (34.68 vs. 34.40) | ***P* = 0.008**  (30.77 vs. 43.89) | *P* = 0.479  (32.86 vs. 35.65 vs. 29.25) |
| CD8-T_con_ in IM | *P* = 0.588  (46.78 vs. 52.57) | *P* = 0.069  (57.13 vs. 47.49) | ***P* = 0.010**  (56.26 vs. 45.54) | *P* = 0.603  (46.97 vs. 51.39) | ***P* ˂ 0.001**  (57.18 vs. 41.42) | ***P* ˂ 0.001**  (61.10 vs. 47.62 vs. 32.32) |
| CD8-T_con_ in TC | *P* = 0.311  (38.34 vs. 45.20) | ***P* ˂ 0.001**  (31.00 vs. 48.15) | ***P* = 0.006**  (35.59 vs. 48.84) | *P* = 0.555  (43.26 vs. 41.83) | ***P* = 0.003**  (35.57 vs. 52.40) | *P* = 0.502  (39.74 vs. 44.48 vs. 33.38) |
| CD8-T_reg_ in IM | *P* = 0.853  (111.11 vs. 109.51) | ***P* = 0.004**  (122.41 vs. 101.68) | ***P* = 0.001**  (119.24 vs. 95.56) | *P* = 0.533  (111.84 vs. 110.20) | ***P* ˂ 0.001**  (119.91 vs. 85.40) | ***P* = 0.011**  (121.92 vs. 107.76 vs. 77.65) |
| CD8-T_reg_ in TC | *P* = 0.089  (73.71 vs. 82.08) | *P* = 0.116  (72.54 vs. 82.56) | *P* = 0.437  (77.14 vs. 83.17) | *P* = 0.472  (74.98 vs. 80.99) | *P* = 0.399  (74.61 vs. 85.12) | *P* = 0.246  (71.89 vs. 82.74 vs. 82.39) |
| CD8-CD31 in IM | *P* = 0.854  (22.02 vs. 22.94) | *P* = 0.115  (20.83 vs. 23.51) | *P* = 0.174  (21.91 vs. 23.47) | *P* = 0.105  (21.17 vs. 23.47) | *P* = 0.915  (22.60 vs. 22.63) | *P* = 0.227  (24.96 vs. 22.39 vs. 21.11) |
| CD8-CD31 in TC | *P* = 0.277  (32.37 vs. 35.27) | ***P* ˂ 0.001**  (25.69 vs. 36.65) | ***P* ˂ 0.001**  (30.51 vs. 38.81) | *P* = 0.385  (33.86 vs. 34.46) | ***P* ˂ 0.001**  (30.75 vs. 40.95) | *P* = 0.925  (35.15 vs. 32.90 vs. 34.54) |
| CD8-αSMA in IM | *P* = 0.060  (55.22 vs. 88.89) | *P* = 0.885  (85.13 vs. 72.63) | *P* = 0.295  (85.02 vs. 62.22) | ***P* = 0.003**  (50.37 vs. 88.00) | *P* = 0.424  (71.96 vs. 75.81) | *P* = 0.130  (96.12 vs. 76.67 vs. 49.43) |
| CD8-αSMA in TC | *P* = 0.099  (31.12 vs. 43.52) | *P* = 0.559  (39.53 vs. 40.35) | *P* = 0.619  (41.13 vs. 40.28) | *P* = 0.158  (32.67 vs. 46.81) | *P* = 0.498  (39.70 vs. 43.02) | *P* = 0.151  (49.75 vs. 40.28 vs. 29.65) |

| ***Additional Table S6. The discrepancy of the cancer-cell proximity score of CD8+ T-cell functional subsets among NSCLC patients grouped by clinicopathological factors*** | | | | | | |
| --- | --- | --- | --- | --- | --- | --- |
| **CCPS** | **Age**  **(≤ 60y vs. >60y)** | **Gender**  **(female vs. male)** | **Smoking index**  **(<400 vs. ≥400)** | **ECOG PS**  **(>1 vs. ≤1)** | **Histological subtype**  **(LUAD vs. LUSC)** | **Stage**  **(I vs II vs III)** |
| CD8+T_total_ in IM | ***P* = 0.006**  (2.53 vs. 3.45) | *P* = 0.205  (2.84 vs. 3.02) | *P* = 0.250  (2.84 vs. 3.09) | ***P* ˂ 0.001**  (2.10 vs. 3.17) | *P* = 0.650  (2.86 vs. 3.03) | ***P* = 0.002**  (3.59 vs. 2.69 vs.1.63) |
| CD8+T_total_ in TC | *P* = 0.209  (2.33 vs. 2.82) | ***P* = 0.044**  (3.26 vs. 2.29) | *P* = 0.392  (2.79 vs. 2.15) | *P* = 0.081  (2.19 vs. 2.68) | ***P* = 0.018**  (2.82 vs. 1.79) | ***P* ˂ 0.001**  (3.60 vs. 2.33 vs. 0.85) |
| CD8+ T_predys_ in IM | *P* = 0.630  (0.36 vs. 0.35) | *P* = 0.425  (0.34 vs. 0.35) | ***P* = 0.030**  (0.30 vs. 0.39) | *P* = 0.202  (0.30 vs. 0.36) | ***P* = 0.021**  (0.29 vs. 0.41) | *P* = 0.440  (0.29 vs. 0.35 vs. 0.36) |
| CD8+ T_predys_ in TC | *P* = 0.248  (0.13 vs. 0.14) | ***P* = 0.023**  (0.17 vs. 0.14) | *P* = 0.114  (0.15 vs. 0.13) | *P* = 0.231  (0.11 vs. 0.15) | ***P* = 0.011**  (0.15 vs. 0.12) | ***P* = 0.016**  (0.15 vs. 0.14 vs. 0.07) |
| CD8+ T_dys_ in IM | *P* = 0.647  (0.62 vs. 0.63) | *P* = 0.359  (0.58 vs. 0.67) | *P* = 0.241  (0.58 vs. 0.67) | *P* = 0.093  (0.82 vs. 0.61) | ***P* = 0.002**  (0.53 vs. 0.94) | ***P* = 0.018**  (0.57 vs. 0.61 vs. 1.19) |
| CD8+ T_dys_ in TC | *P* = 0.739  (0.42 vs. 0.40) | *P* = 0.807  (0.42 vs. 0.41) | *P* = 0.863  (0.41 vs. 0.41) | *P* = 0.755  (0.41 vs. 0.41) | *P* = 0.744  (0.42 vs. 0.39) | *P* = 0.281  (0.50 vs. 0.39 vs. 0.28) |
