# Supplementary material for: Functional status and spatial architecture of tumor-infiltrating CD8+ T cells are associated with lymph node metastases in non-small cell lung cancer
Source: J Transl Med. 2023 May 12;21:320. doi: 10.1186/s12967-023-04154-y (PMC10182600; doi:10.1186/s12967-023-04154-y)

Supplementary file-2

Functional status and spatial architecture of tumor-infiltrating CD8+ T cells are associated with lymph node metastases in non-small cell lung cancer

Guanqun Yang[#], Siqi Cai[#], Mengyu Hu, Chaozhuo Li, Liying Yang, Wei Zhang, Jujie Sun, Fenghao Sun, Ligang Xing, Xiaorong Sun[*]

Overview of multiplex stained sections from all tissue microarray before and after fluorescence imaging

Panel 1

TMA 01

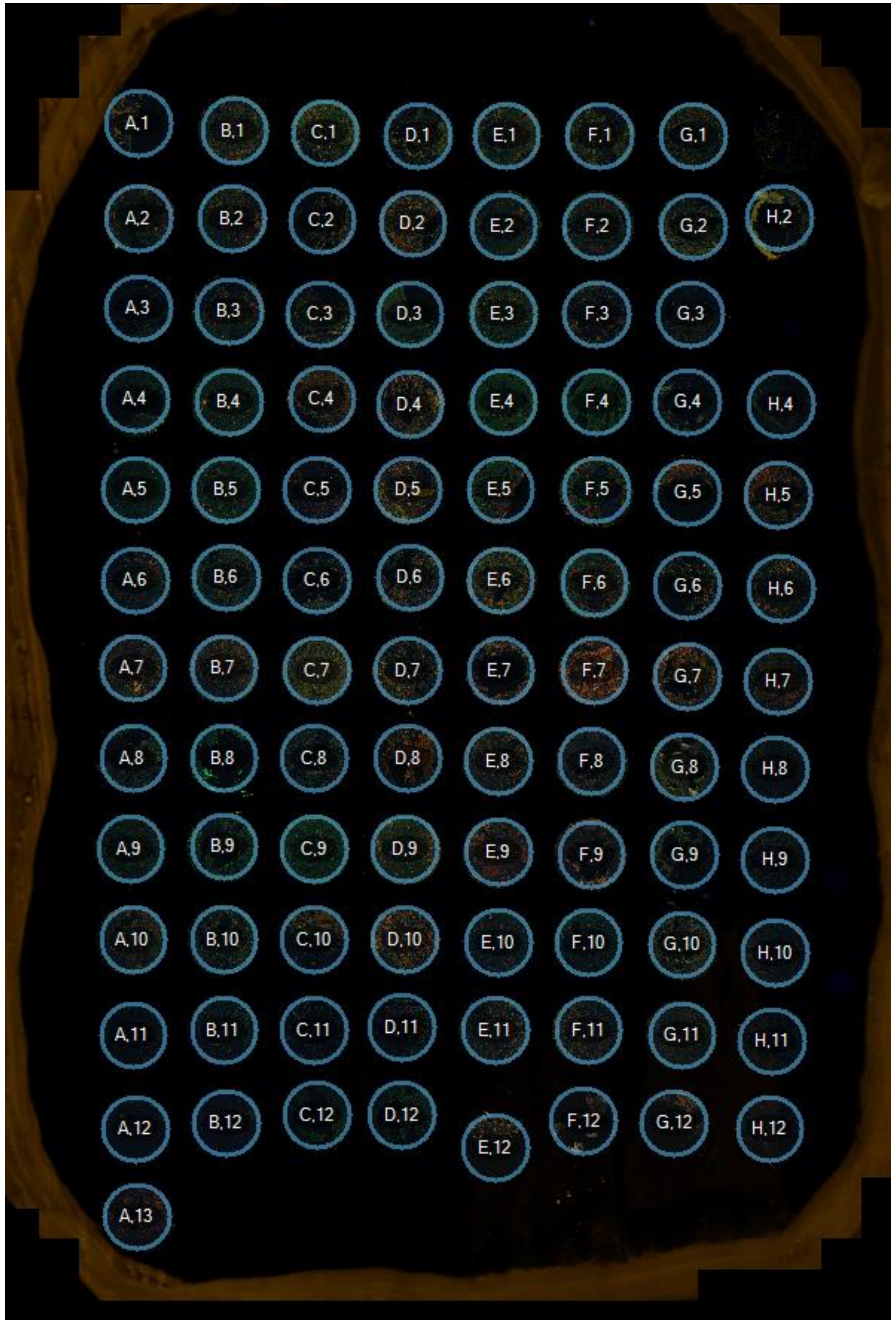

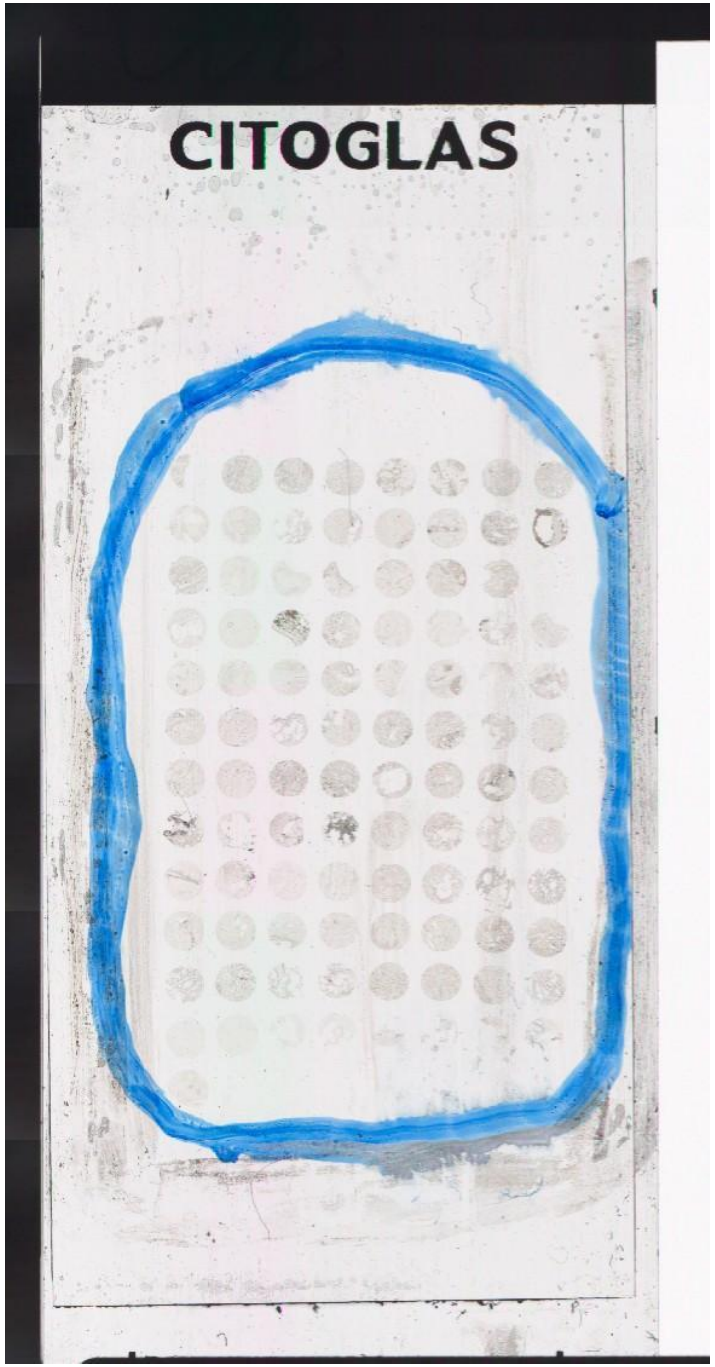

Panel 1

TMA 02

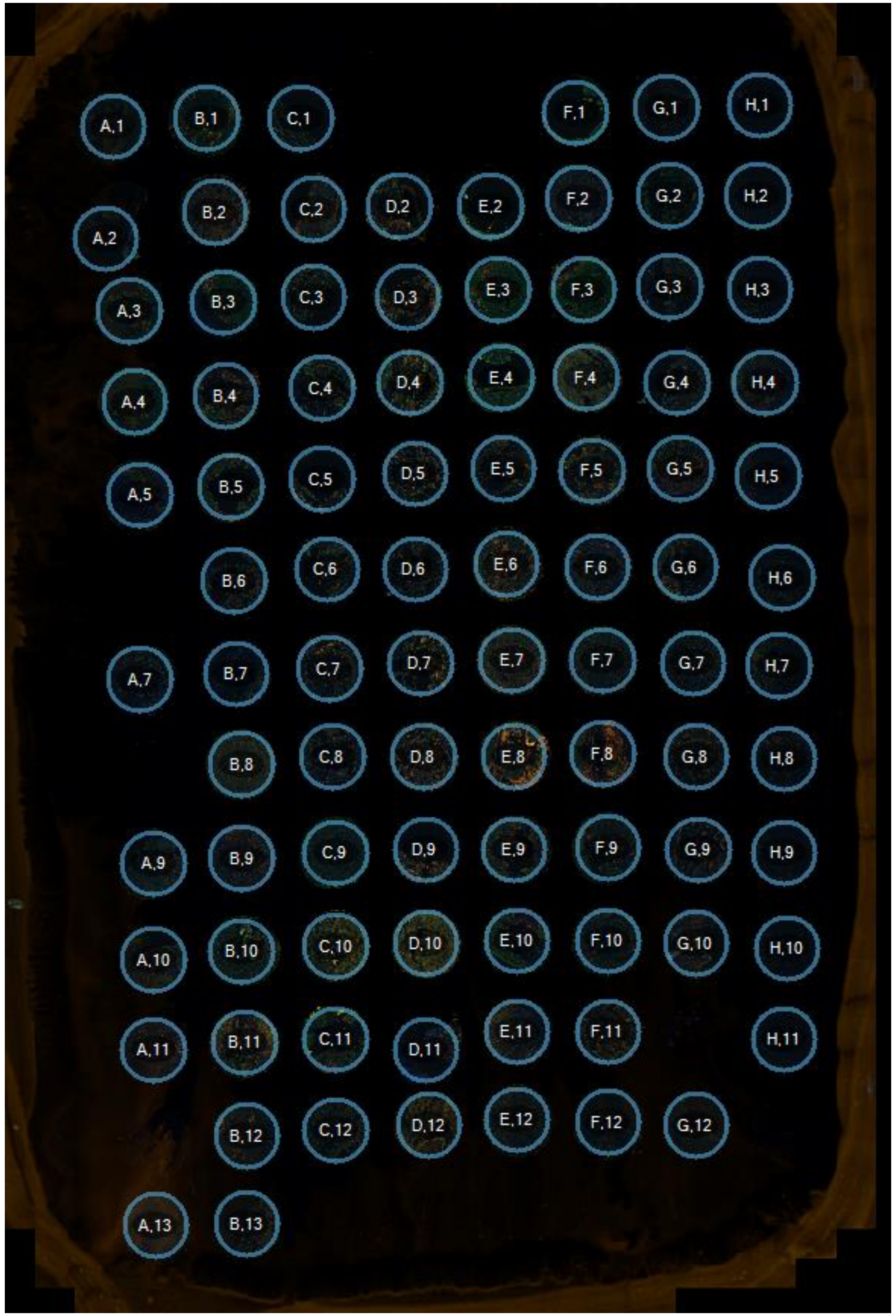

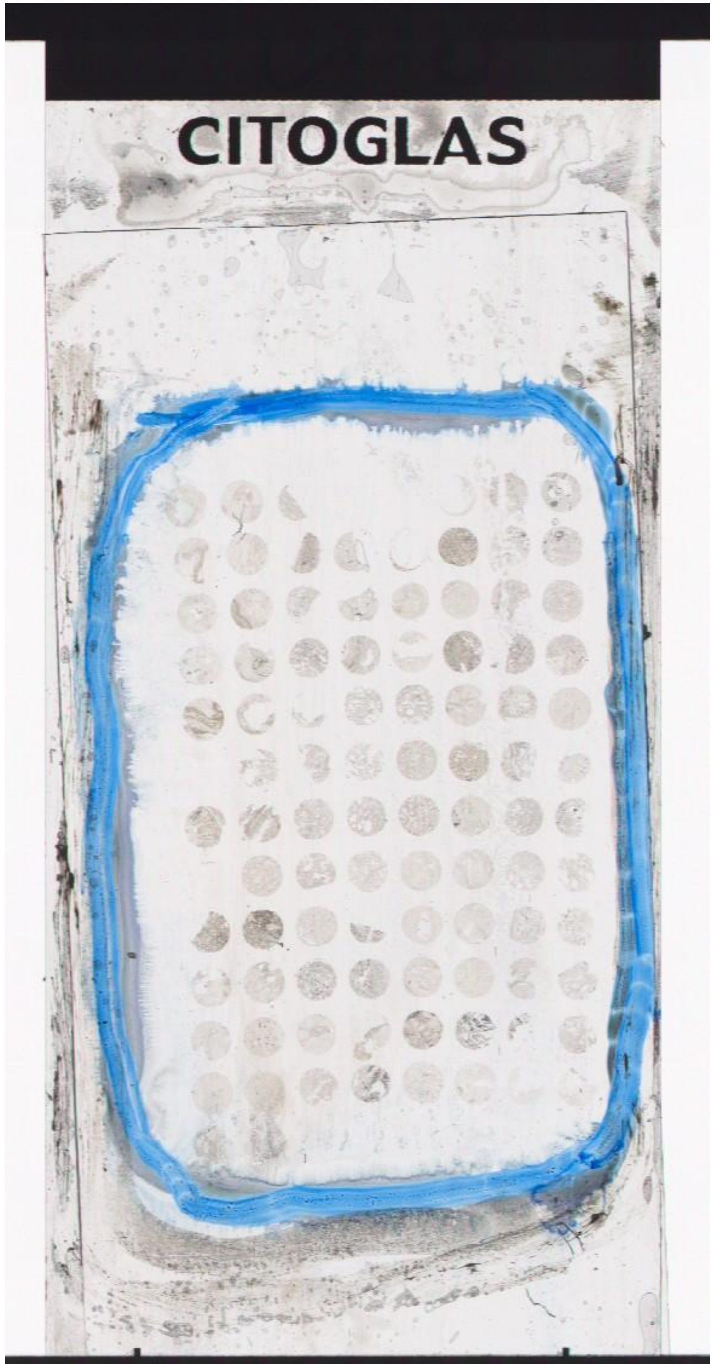

Panel 1

TMA 03

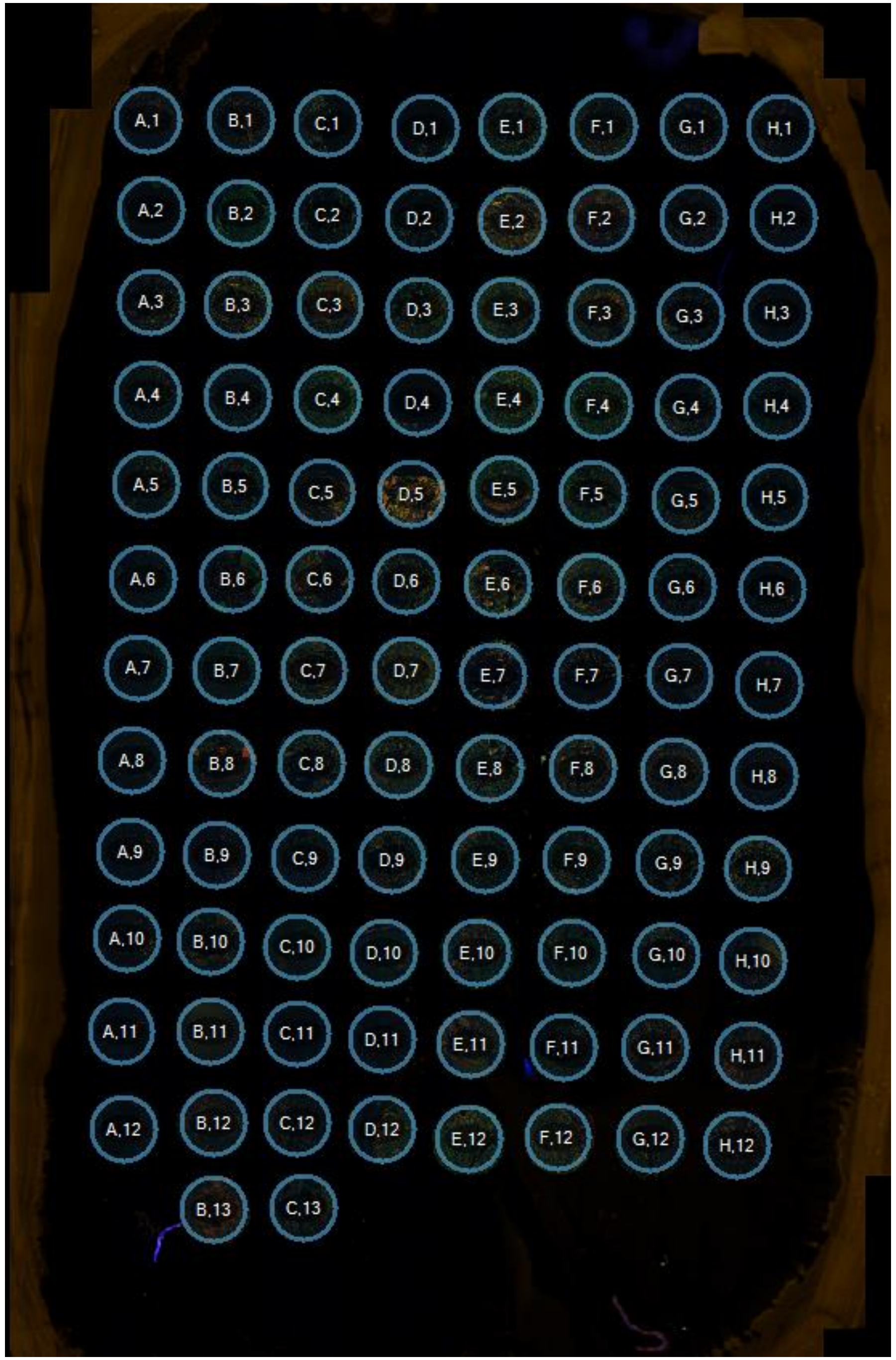

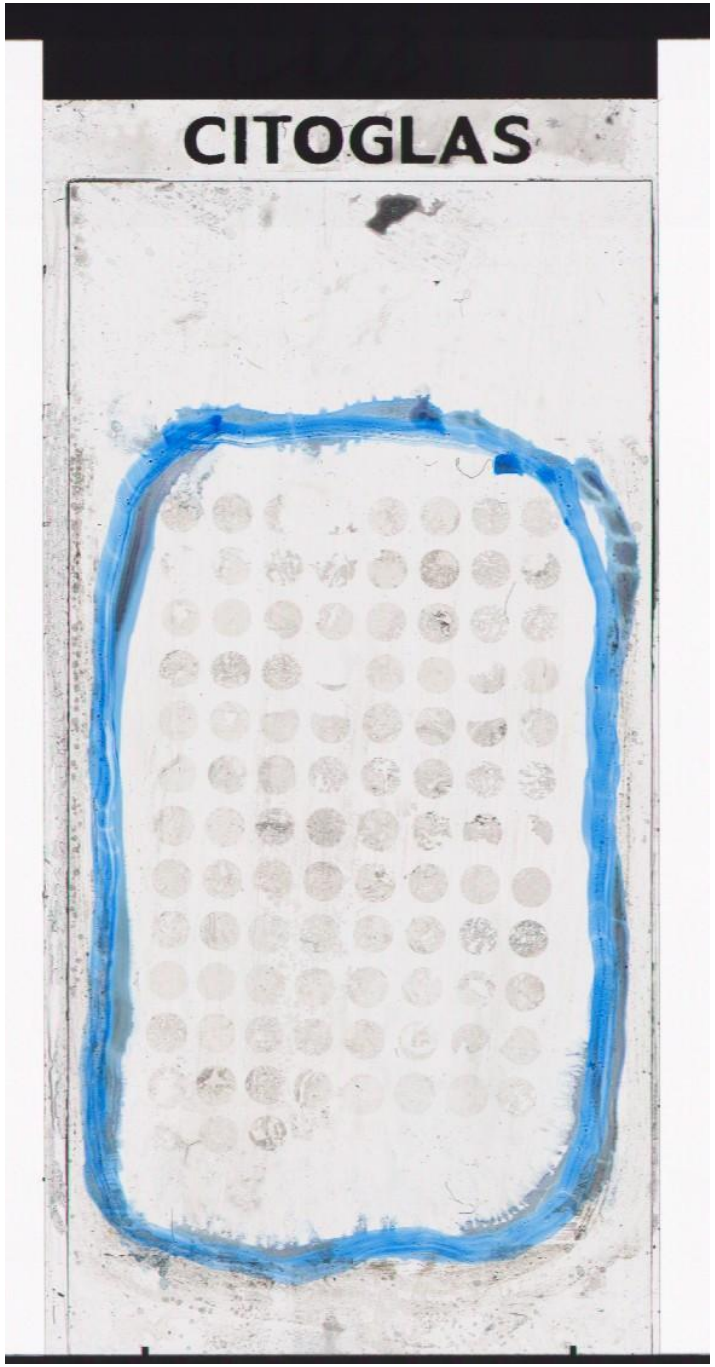

Panel 1

TMA 04

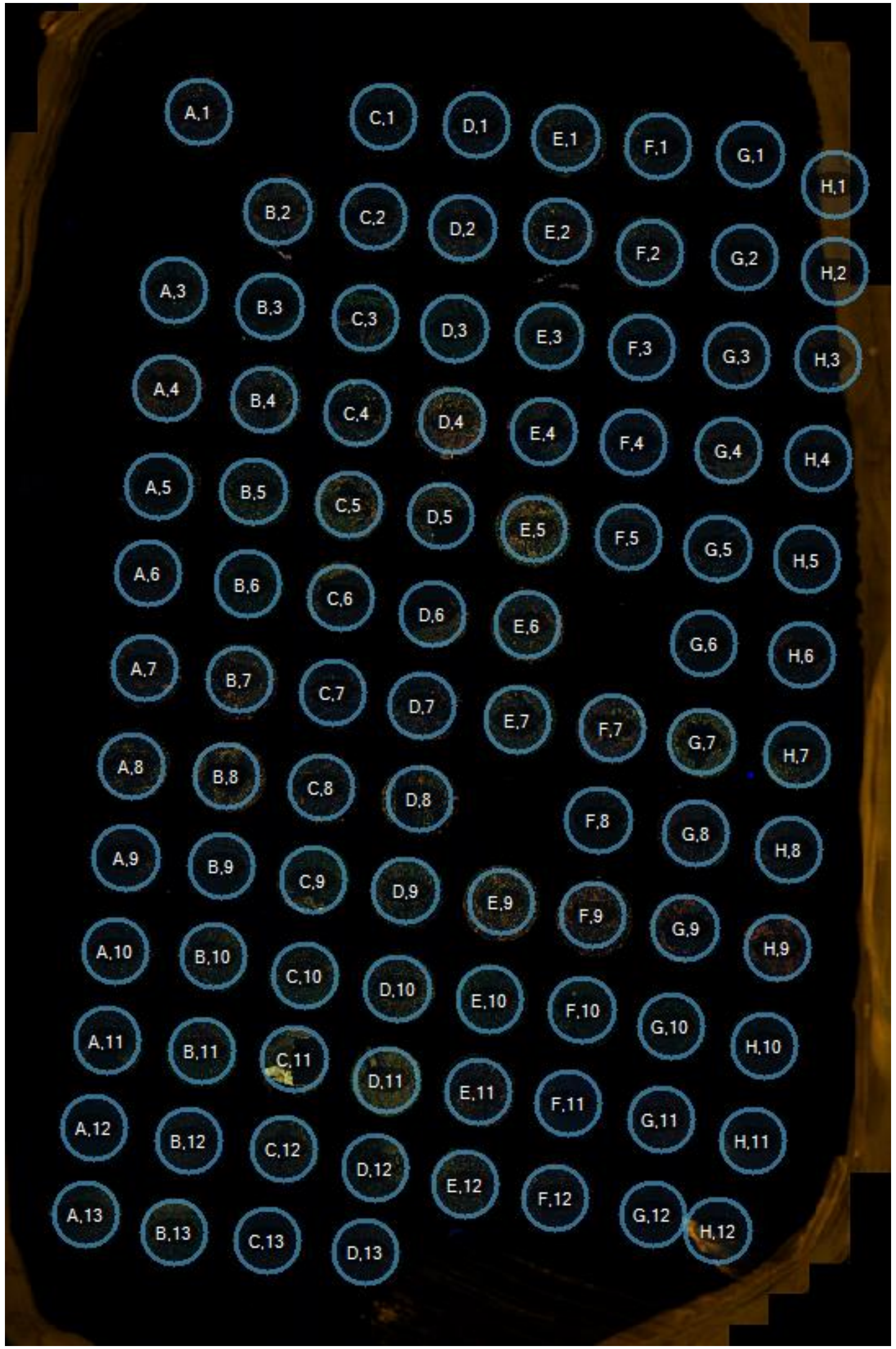

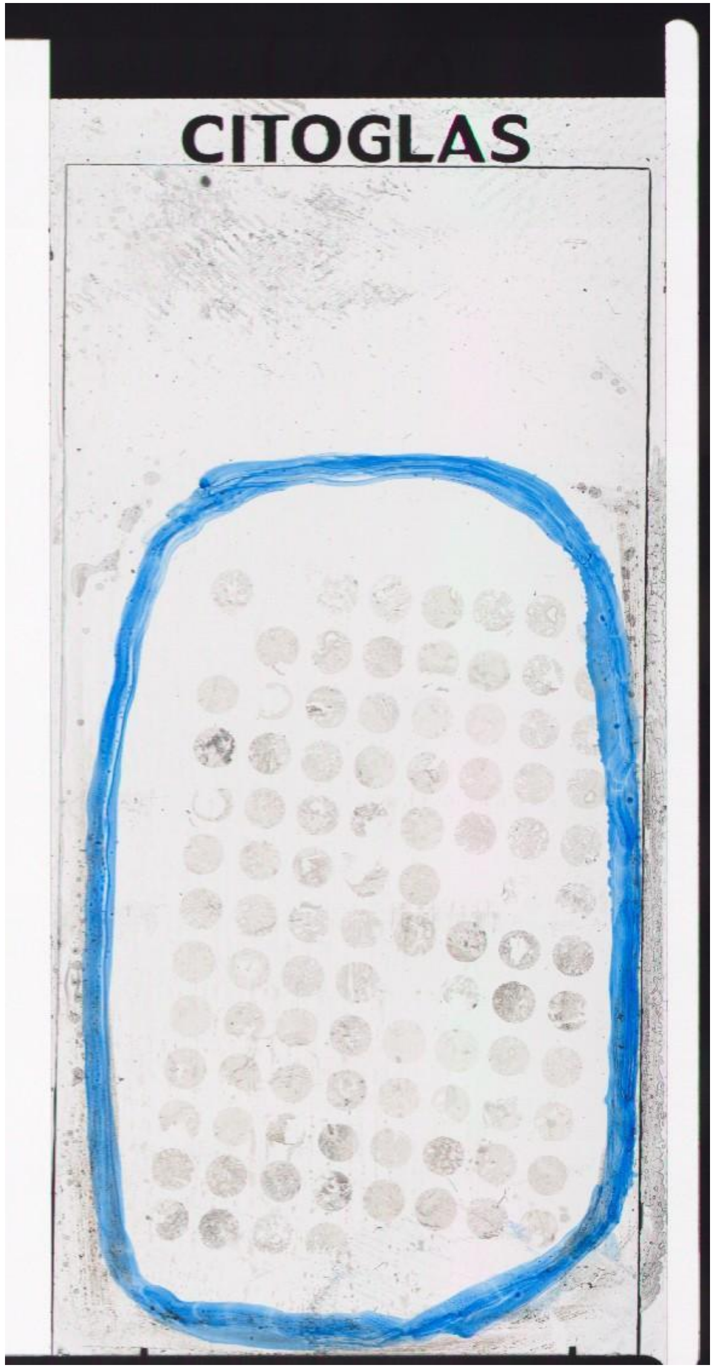

Panel 1

TMA 05

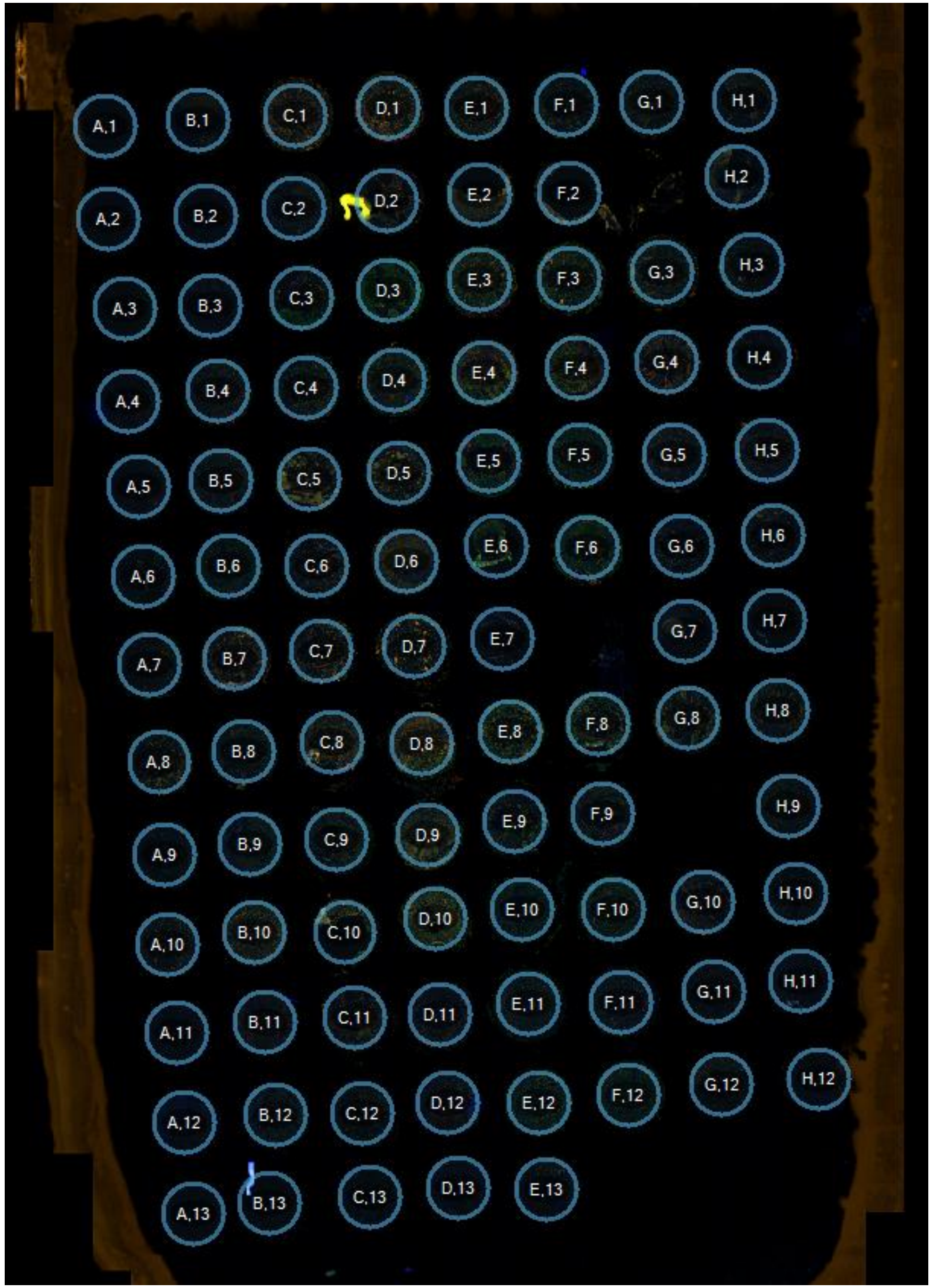

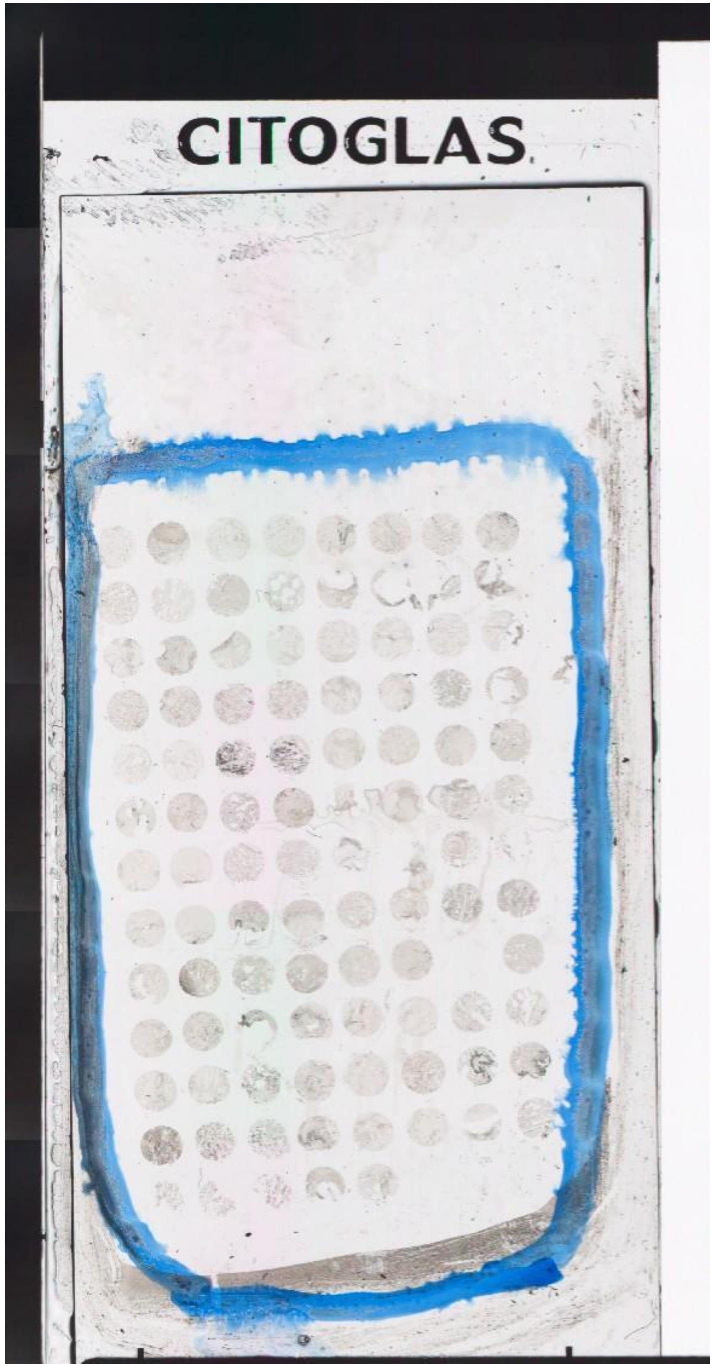

Panel 1

TMA 06

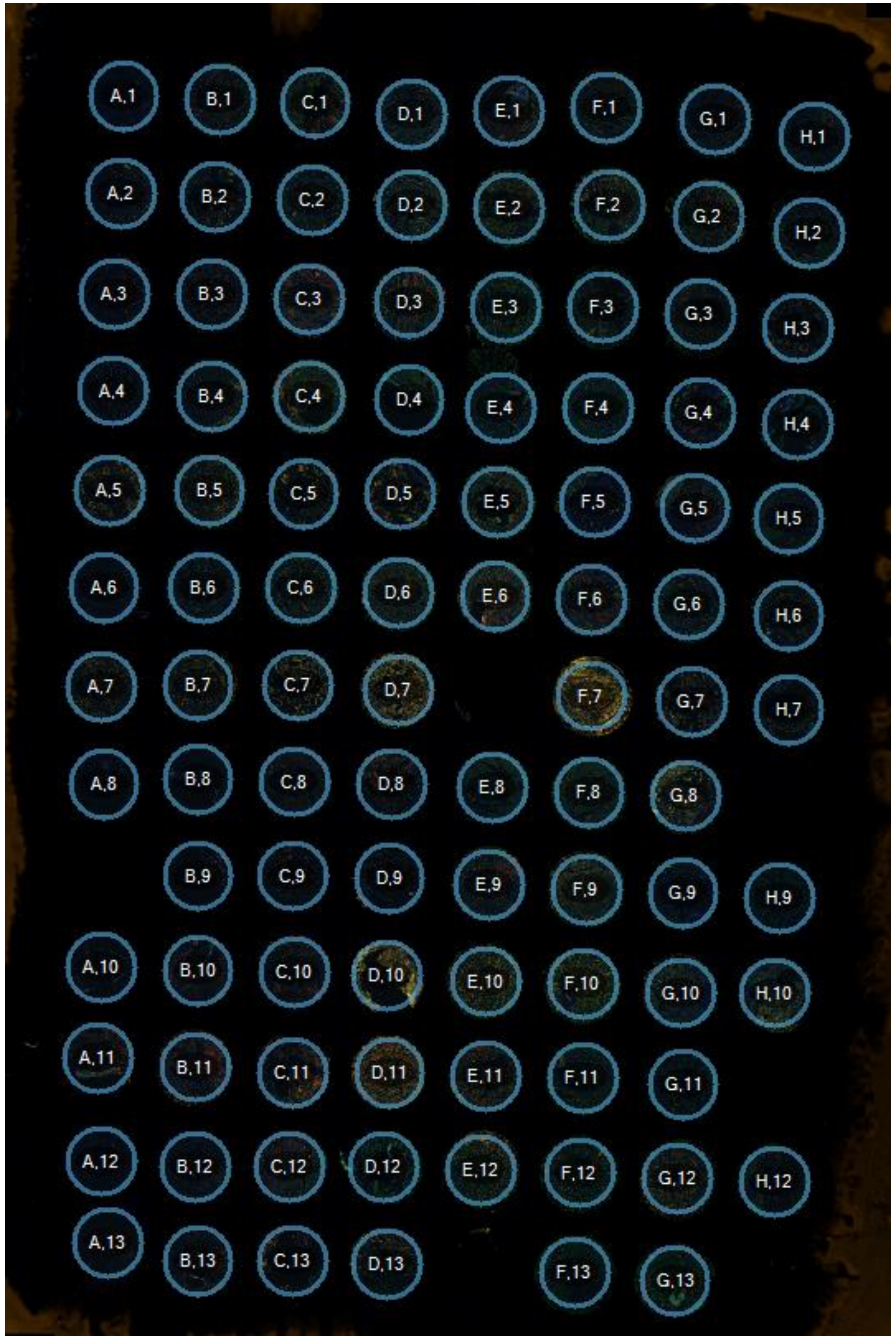

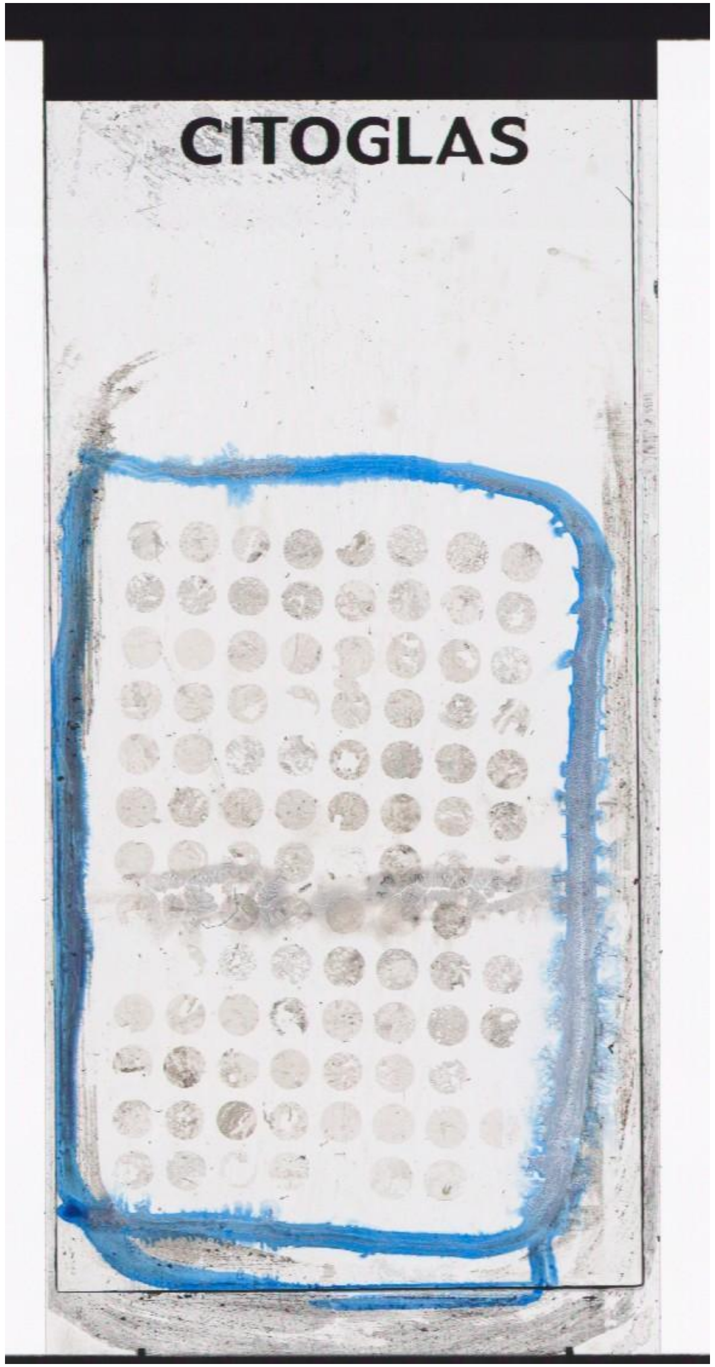

Panel 1

TMA 07

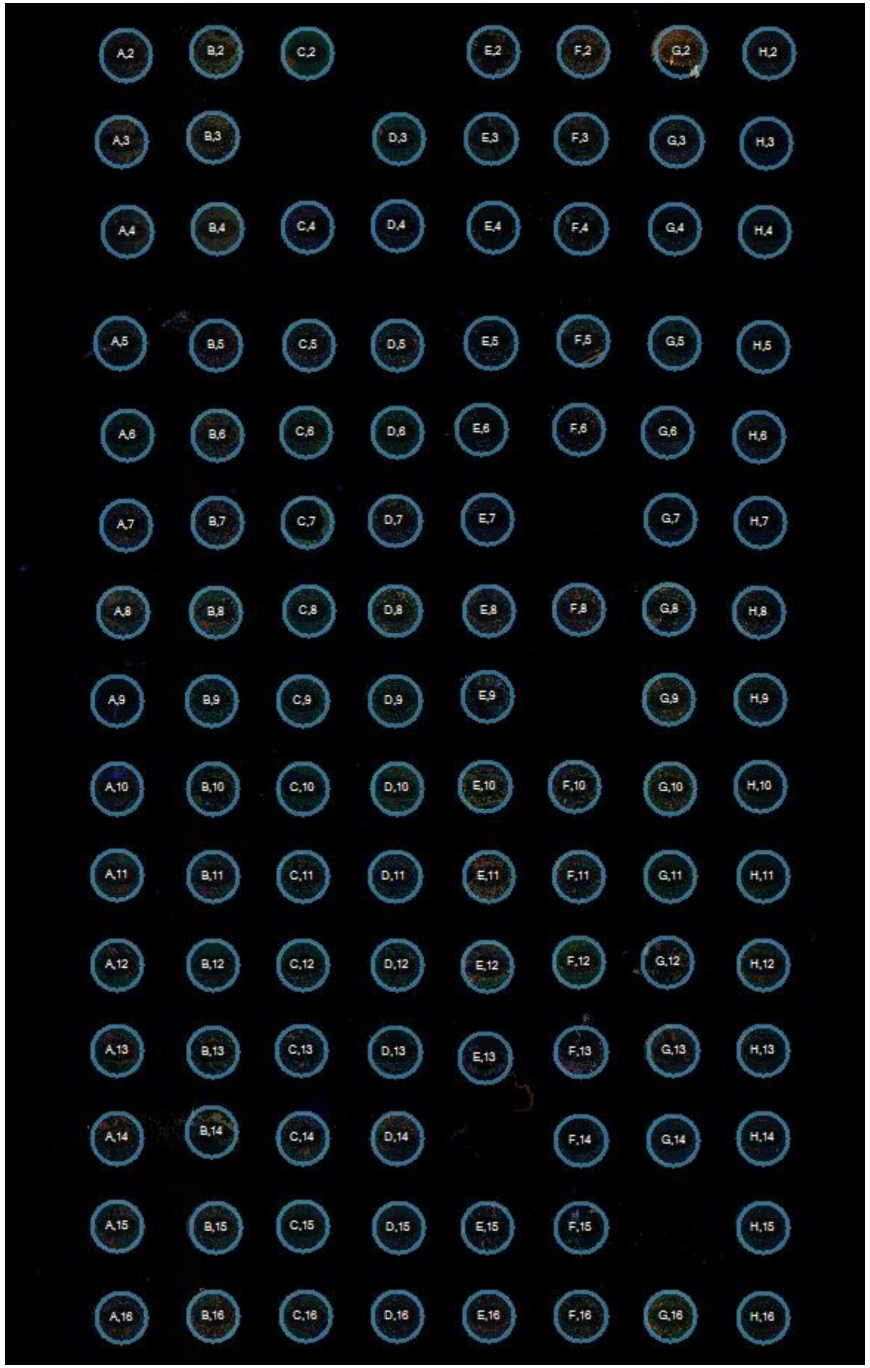

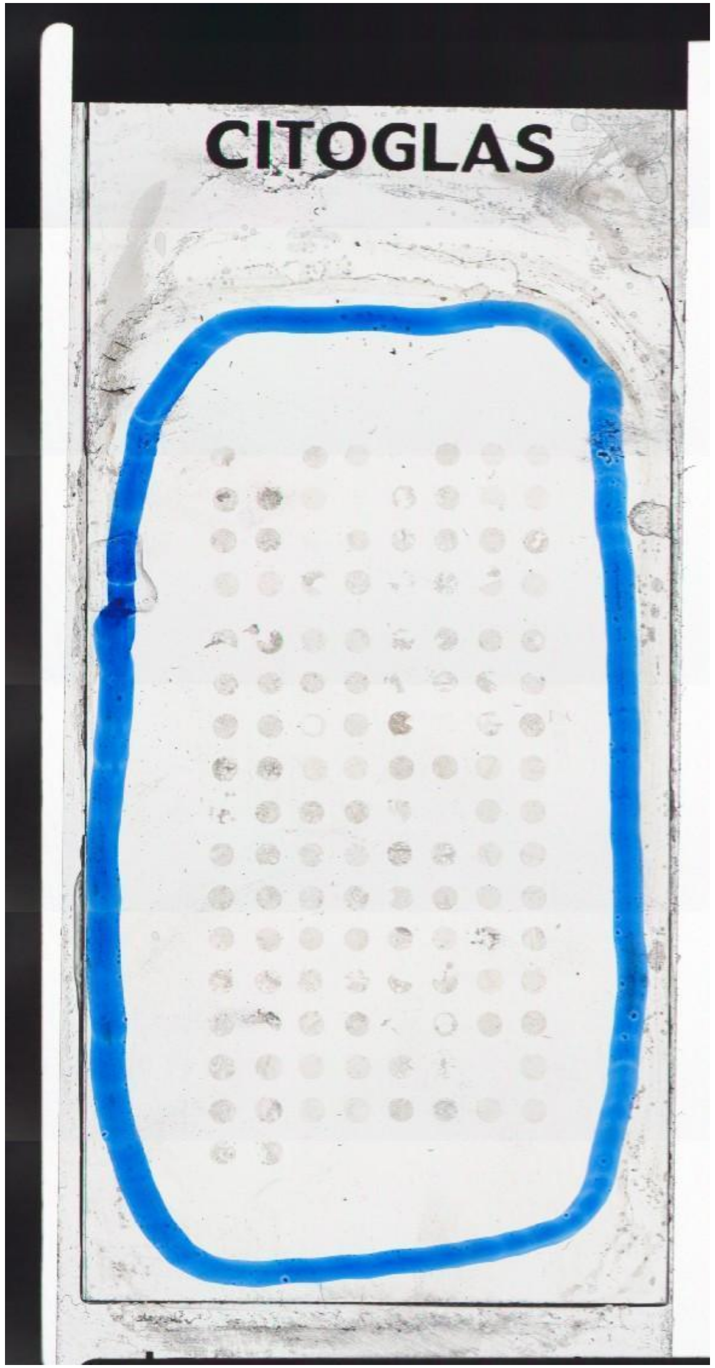

Panel 1

TMA 08

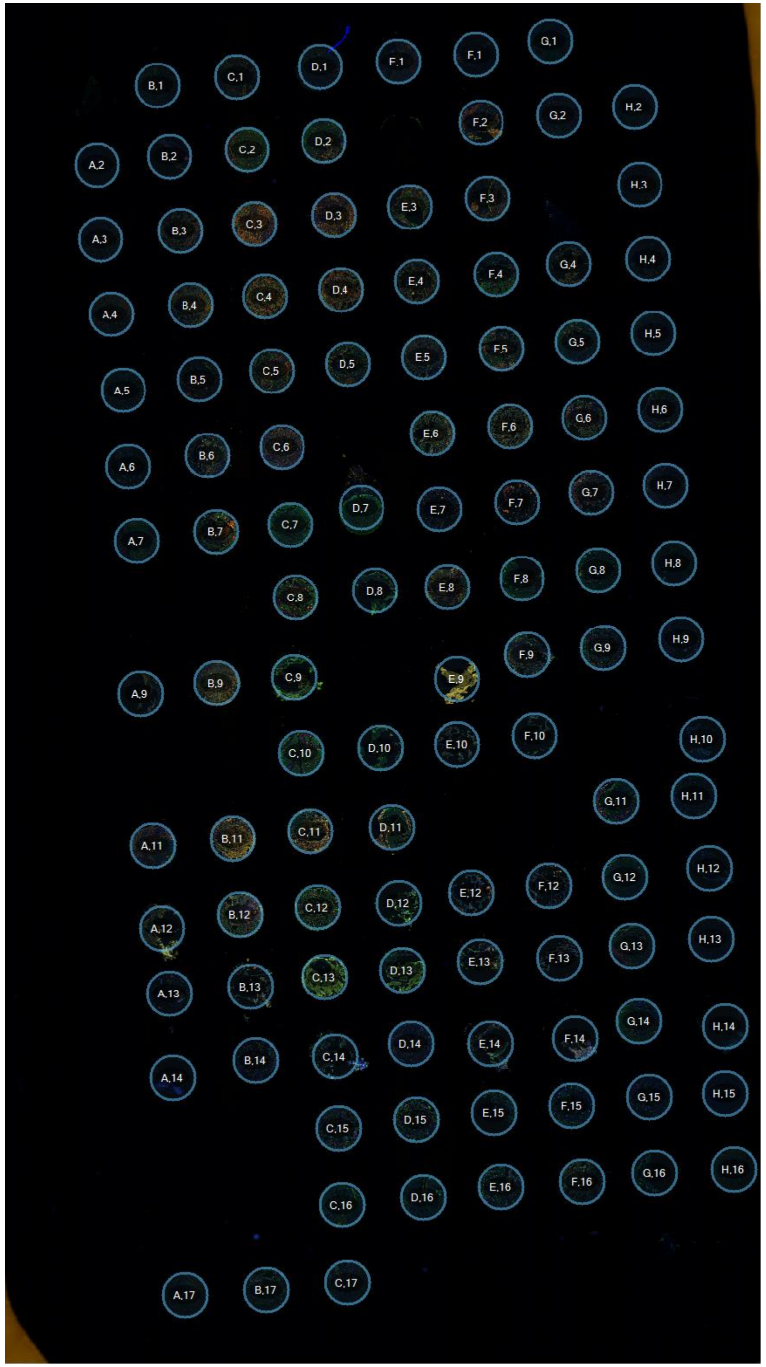

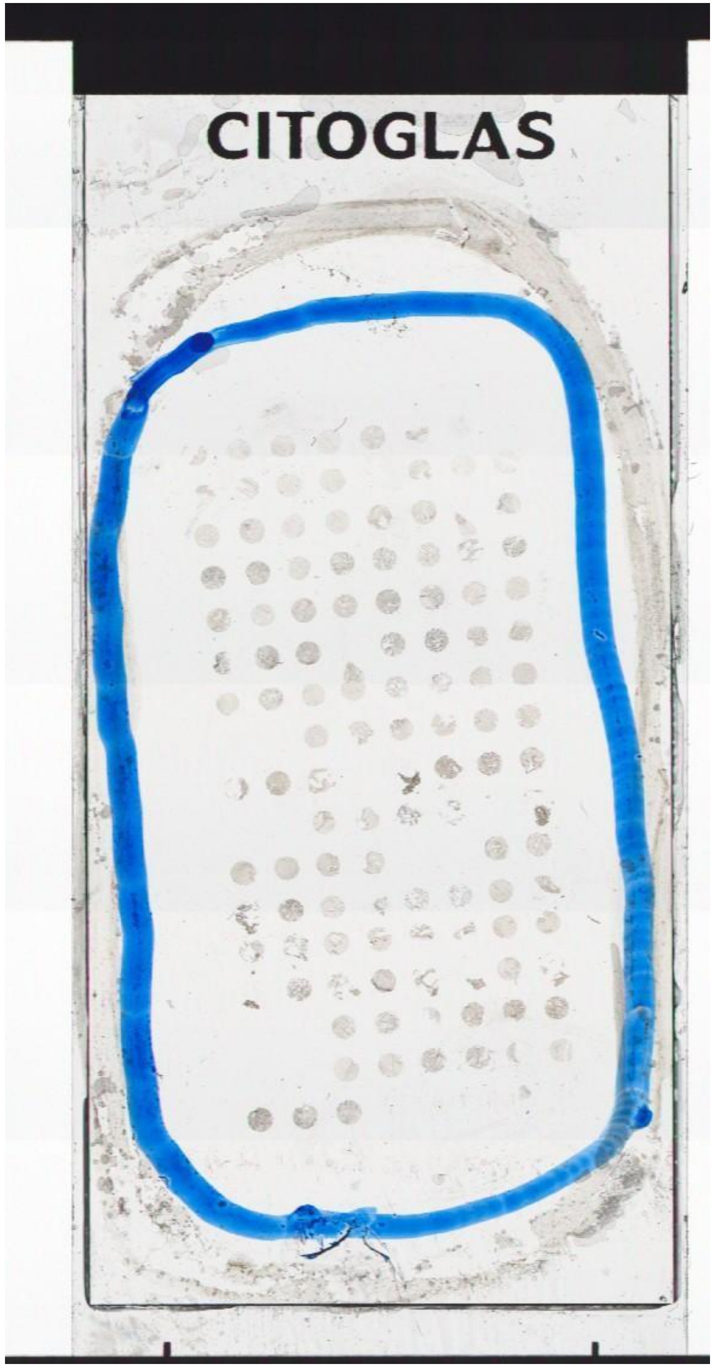

Panel 1

TMA 09

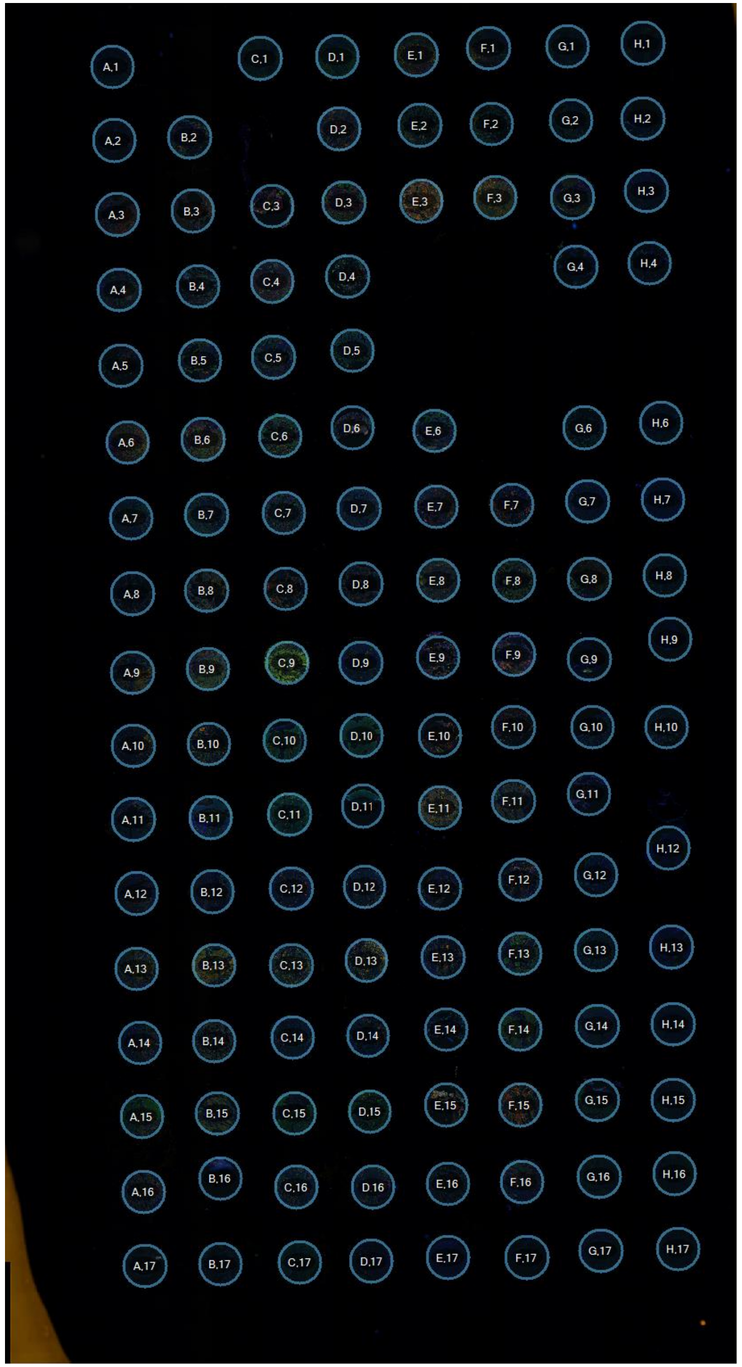

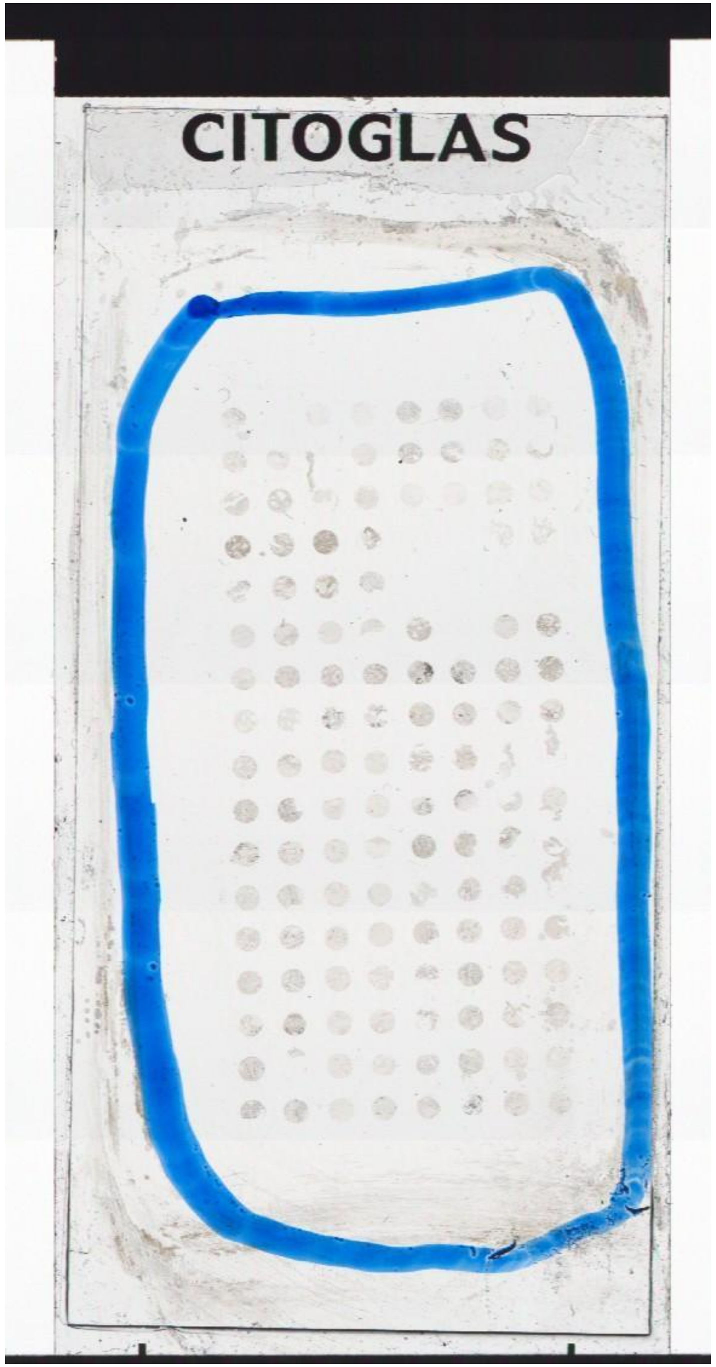

Panel 1

TMA 10

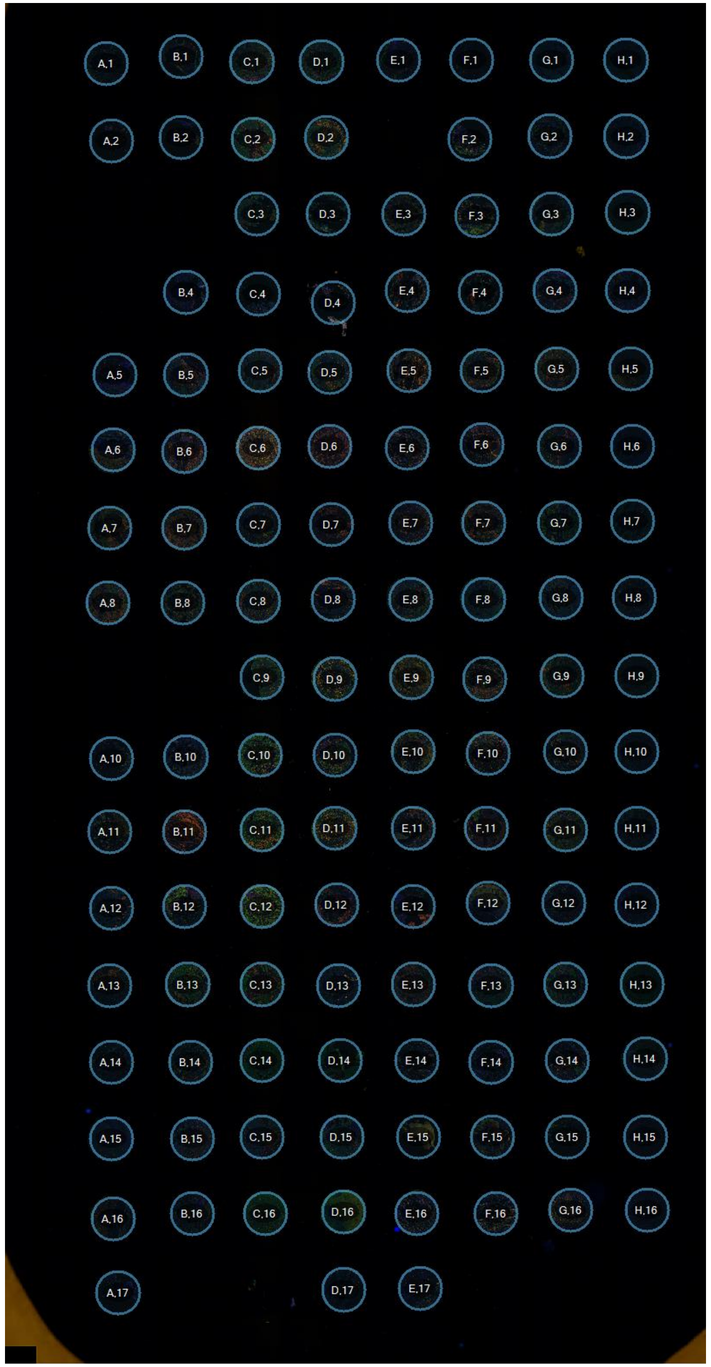

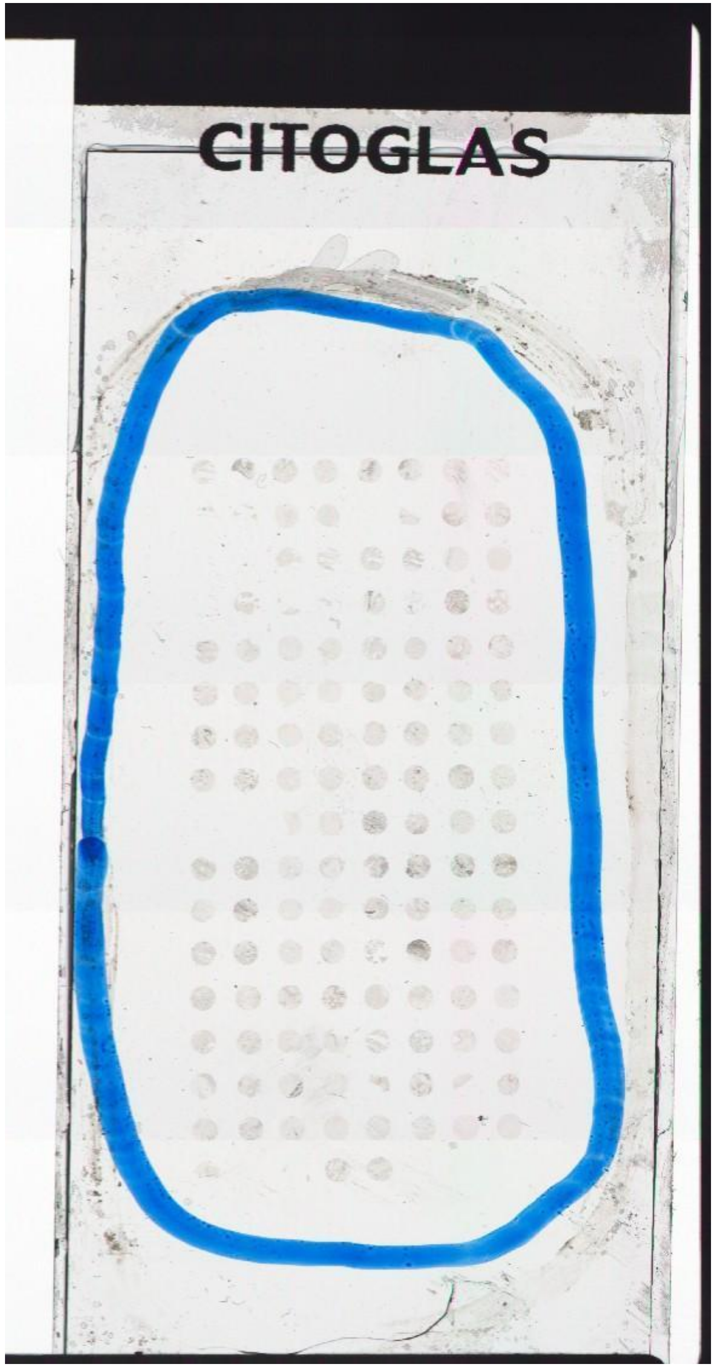

Panel 1

TMA 11

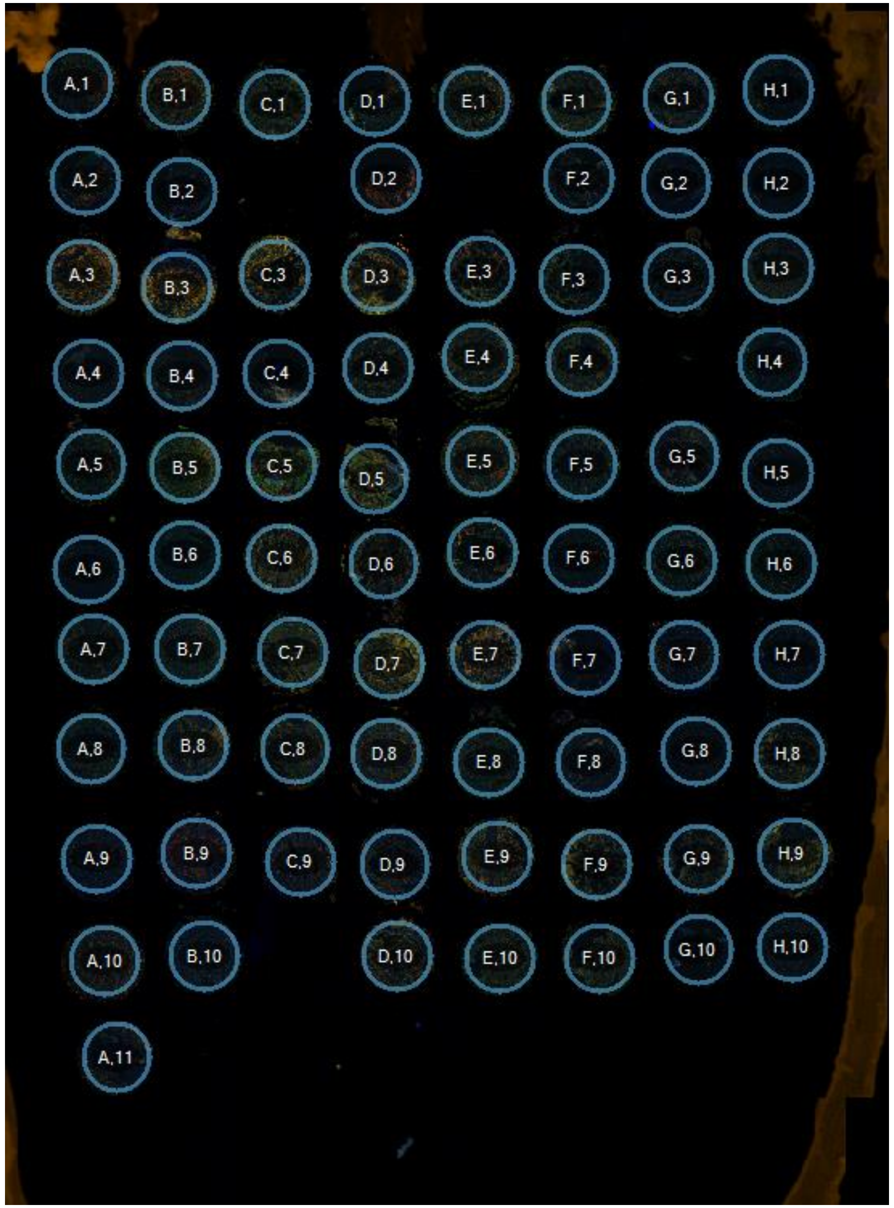

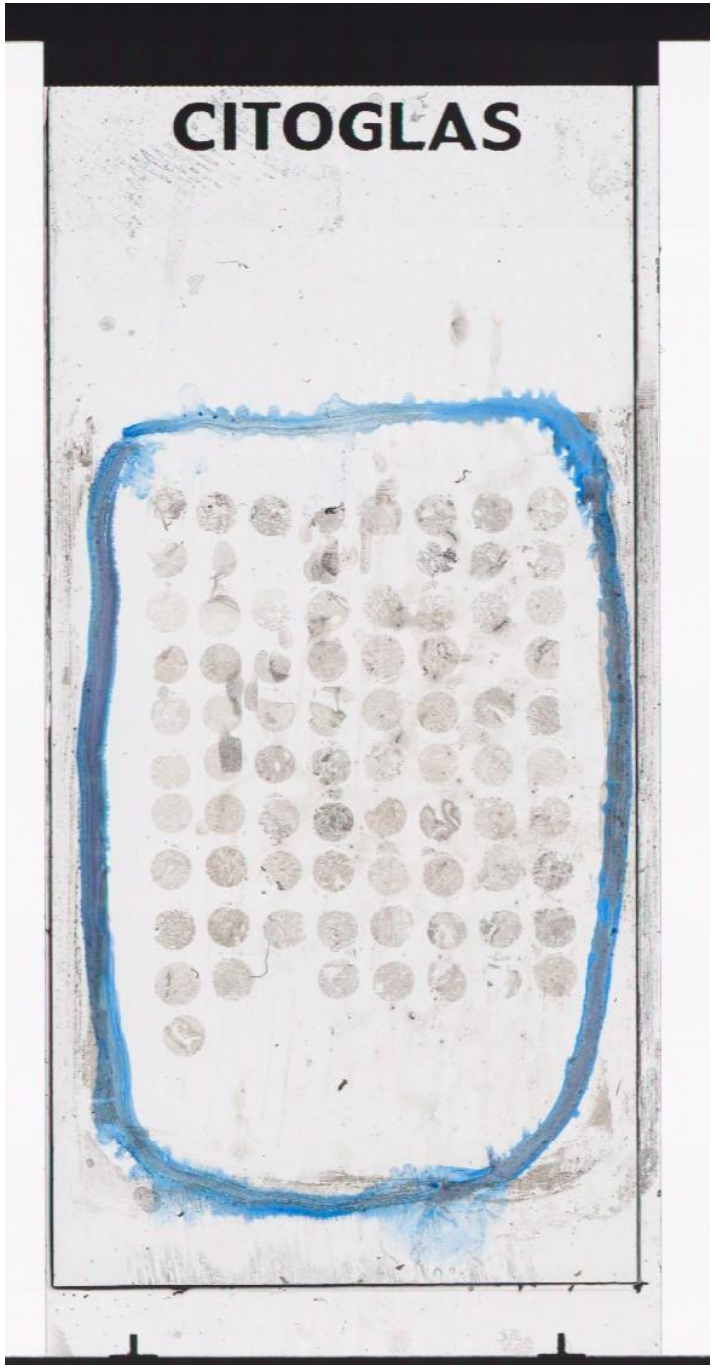

Panel 1

TMA 12

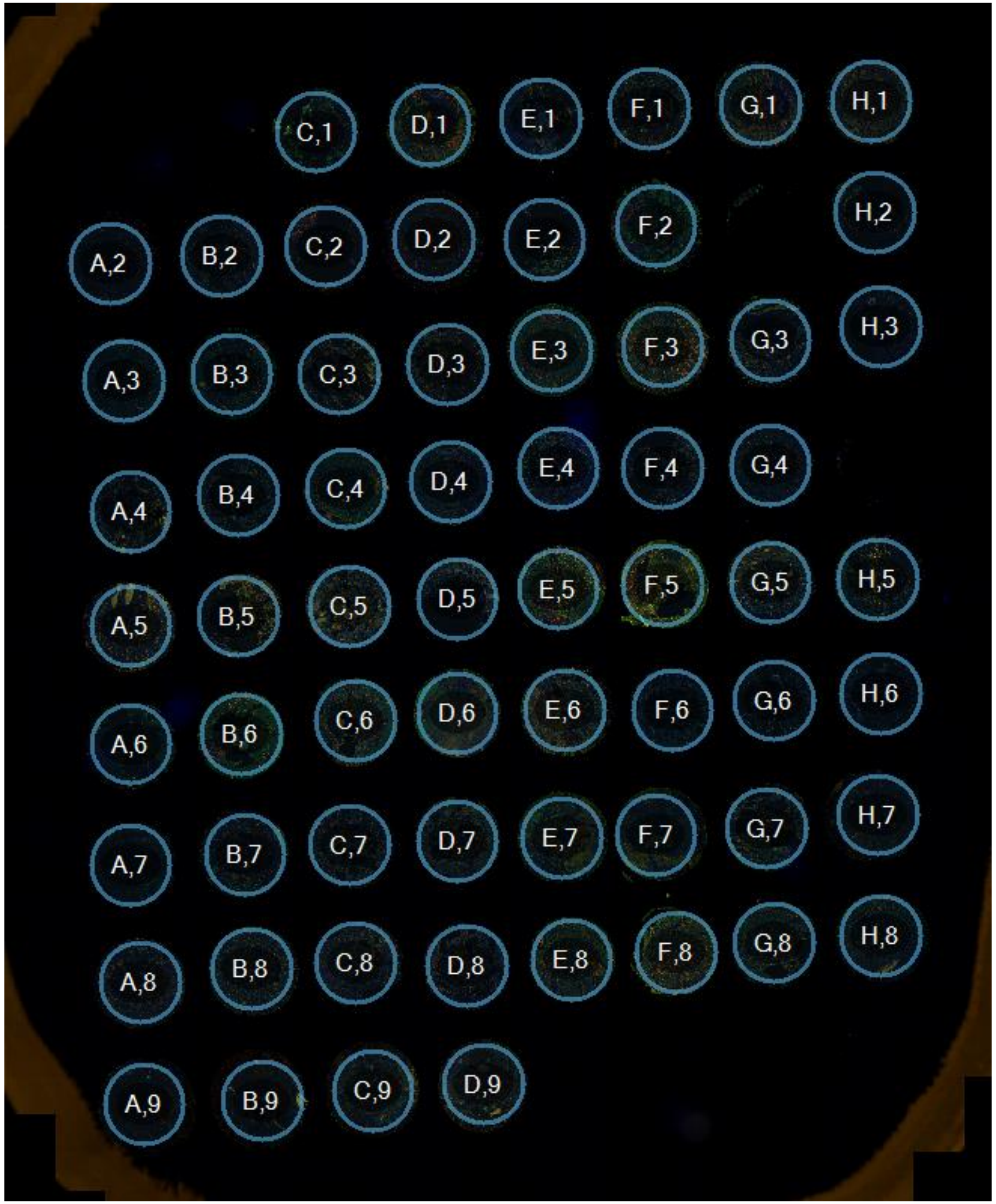

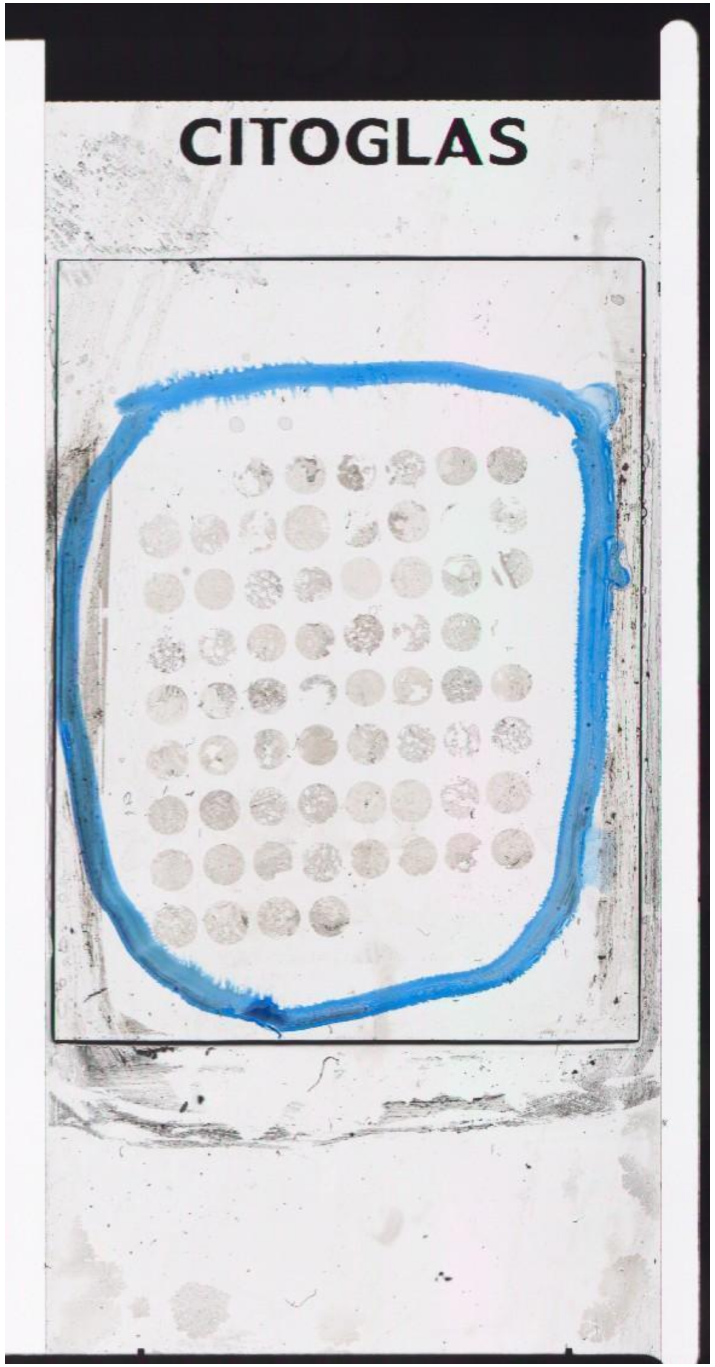

Panel 2

TMA 13

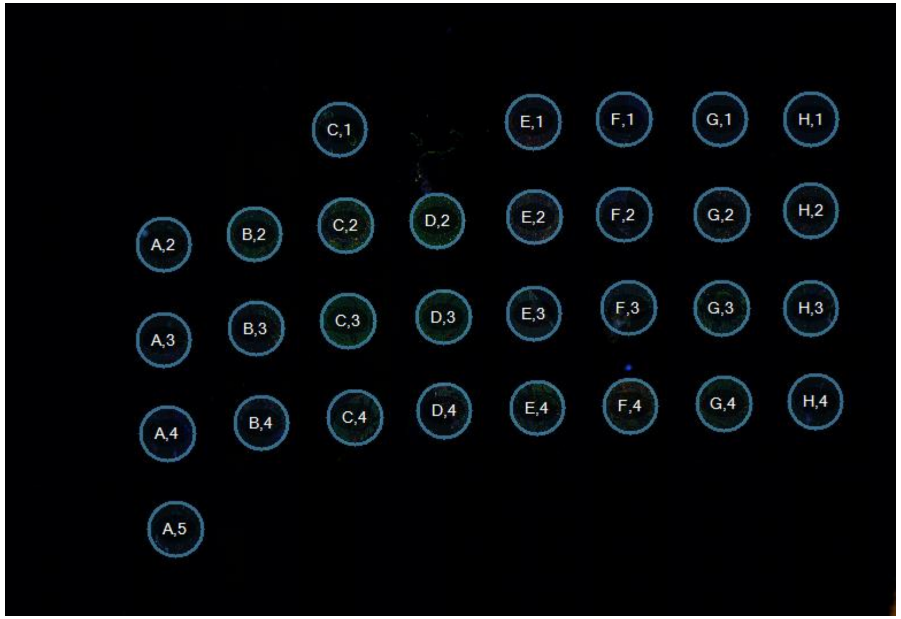

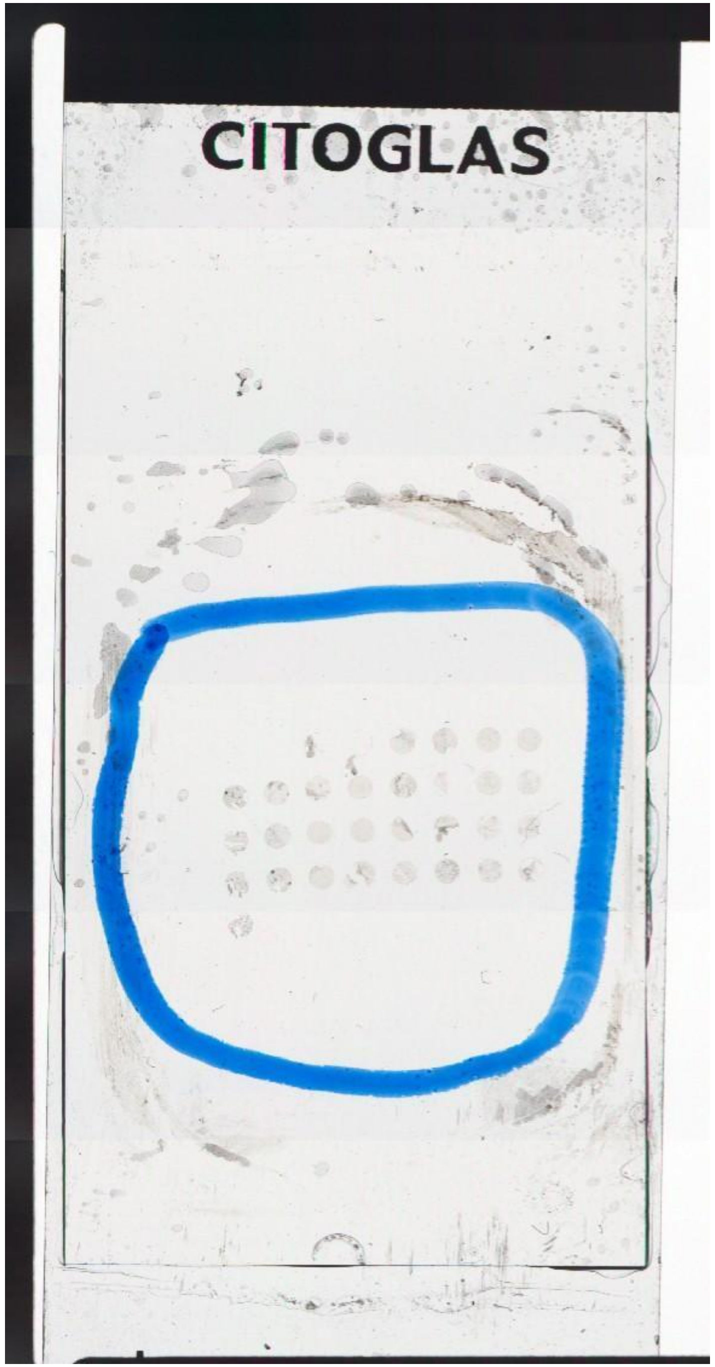

Panel 2

TMA 01

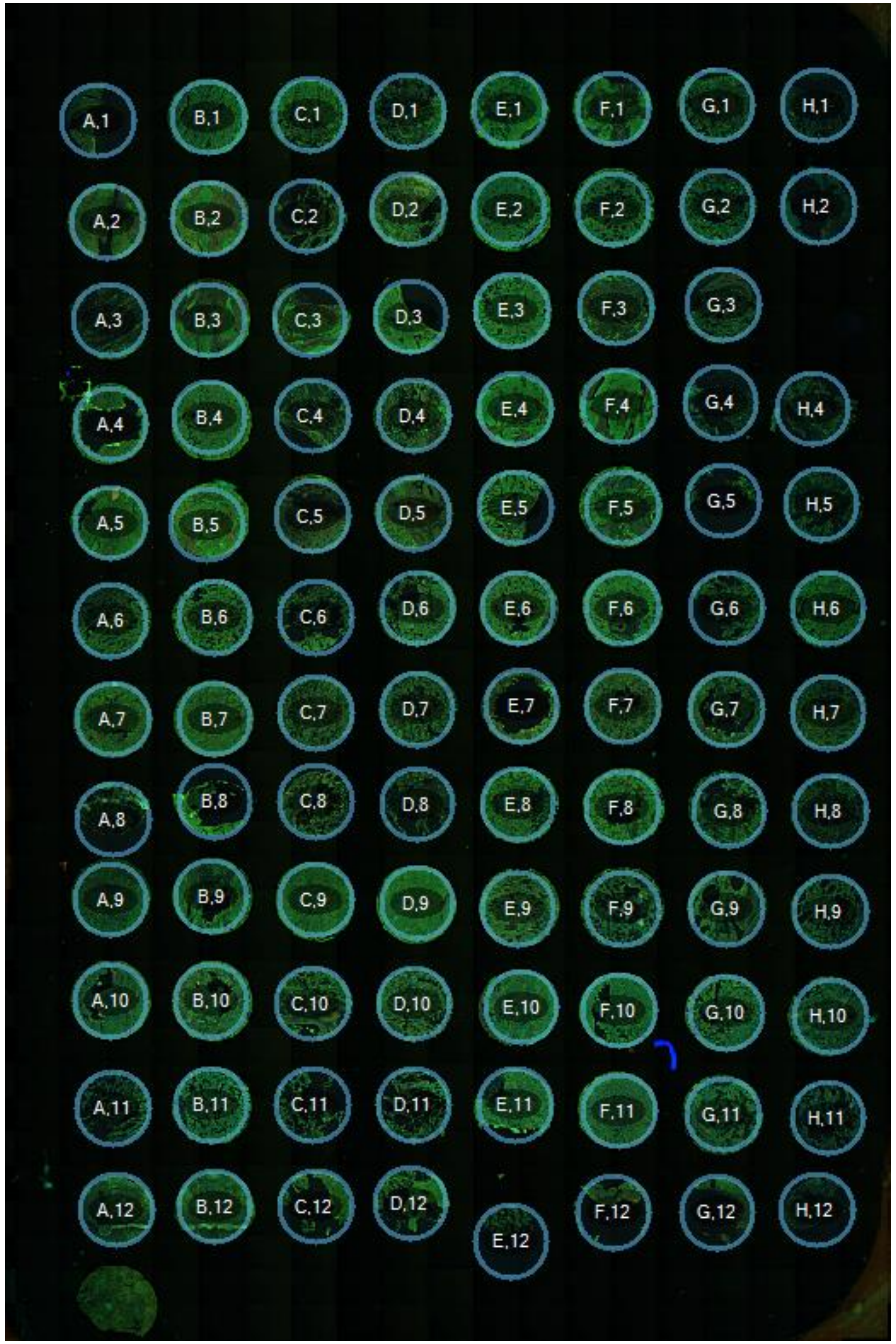

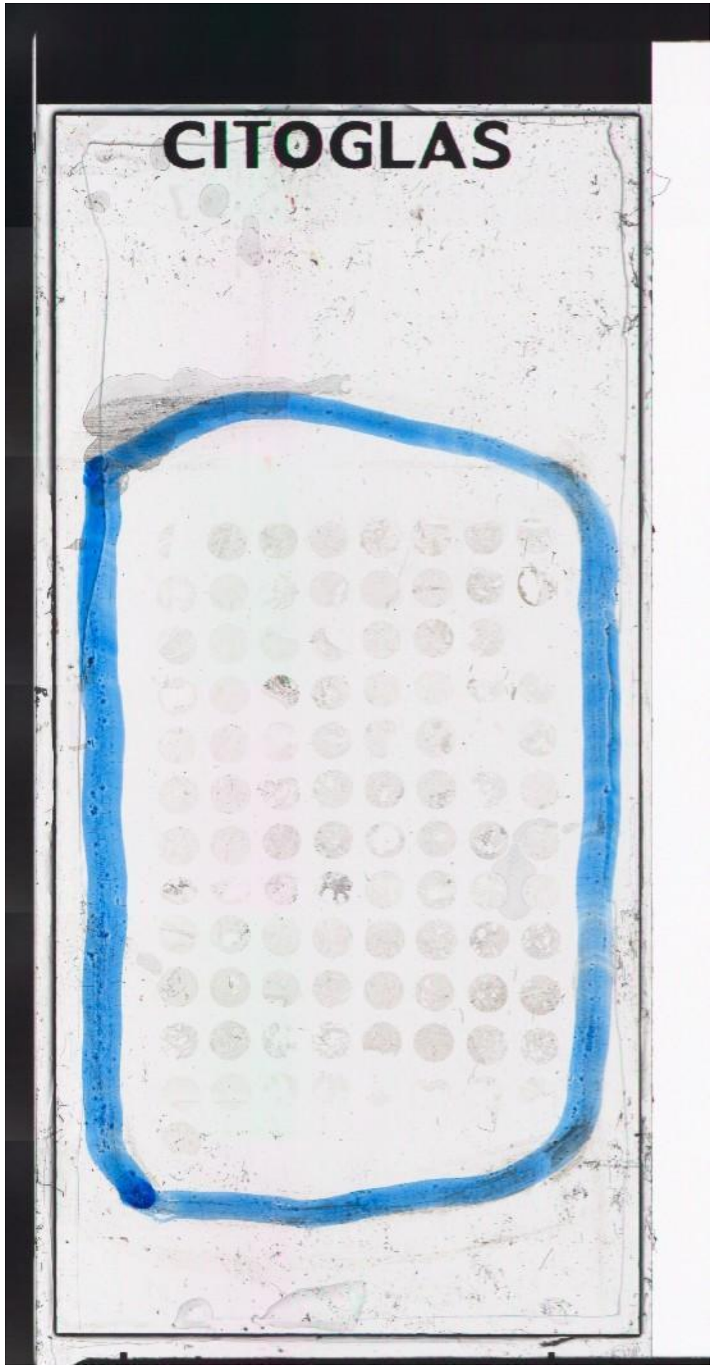

Panel 2

TMA 02

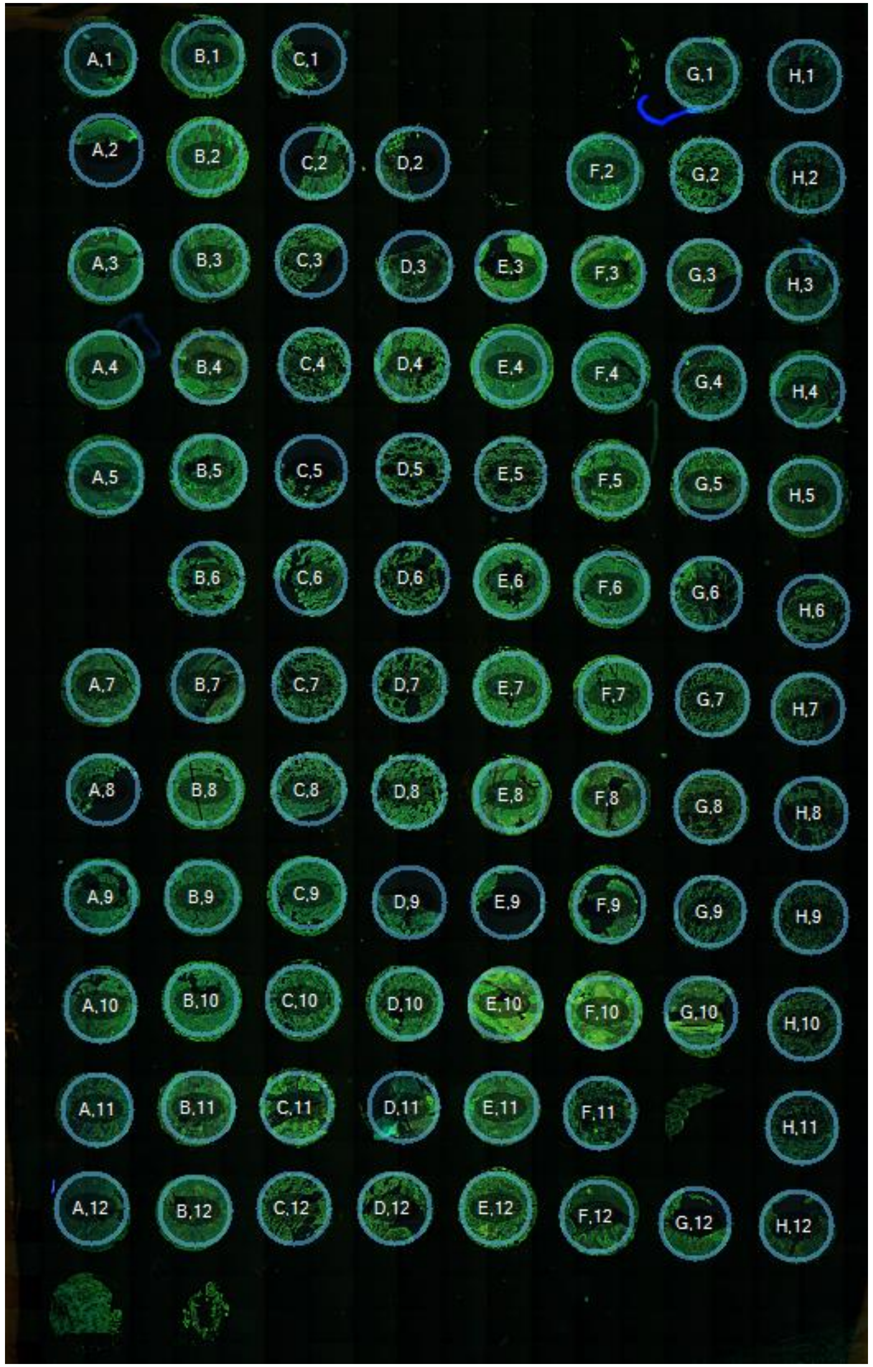

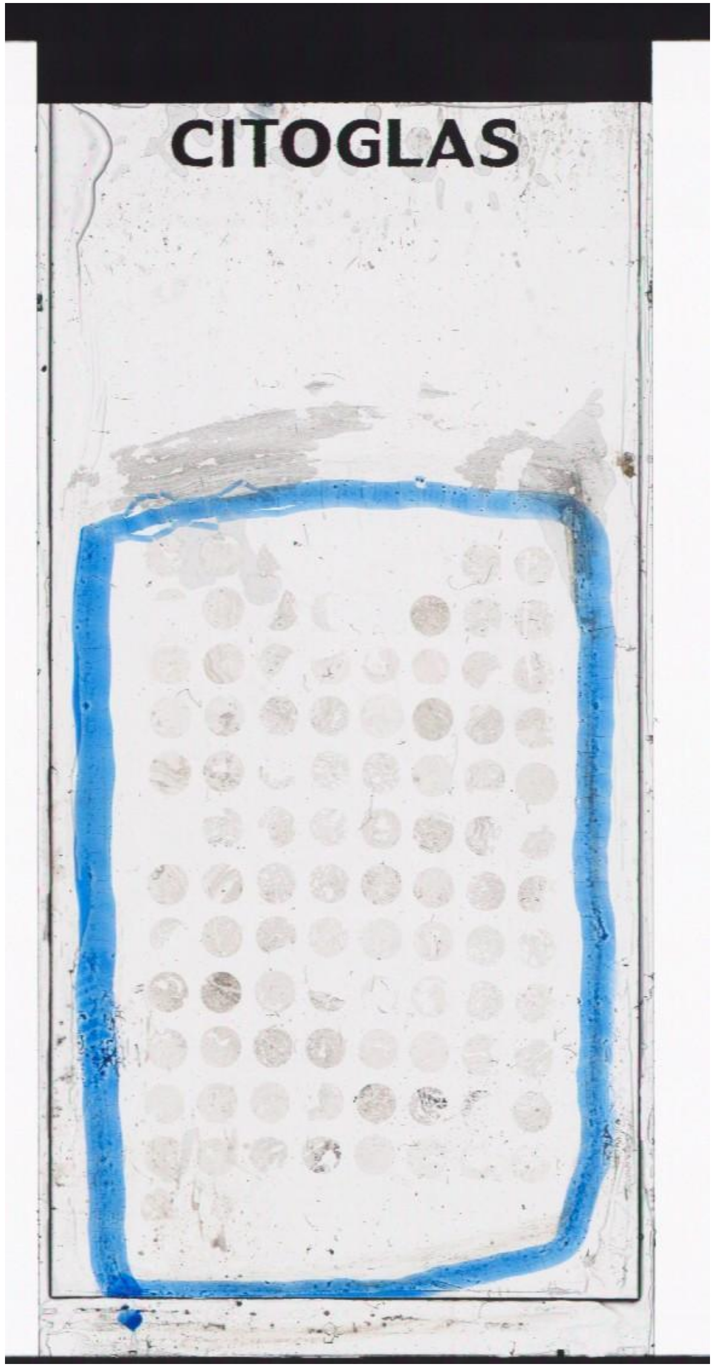

Panel 2

TMA 03

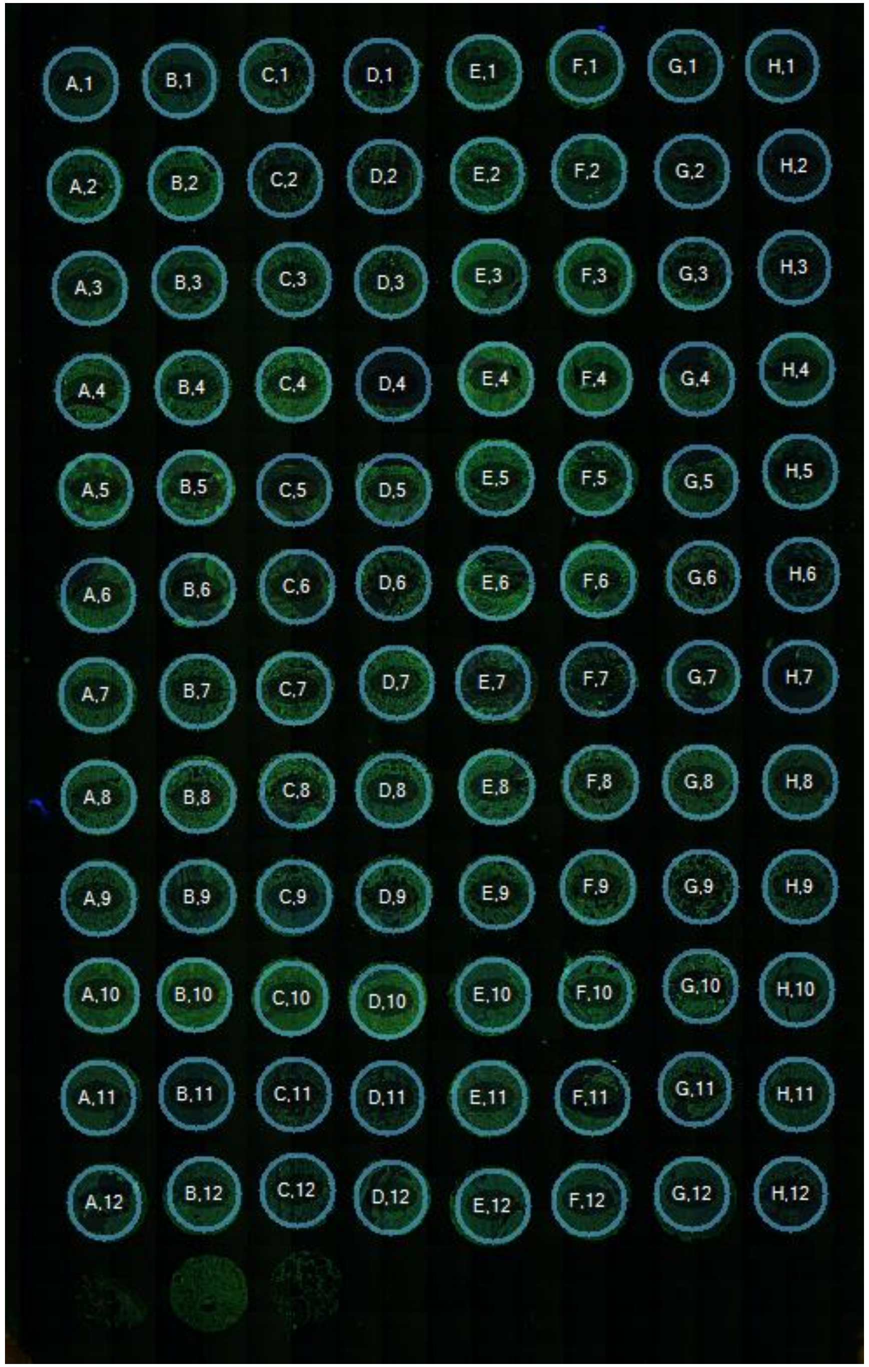

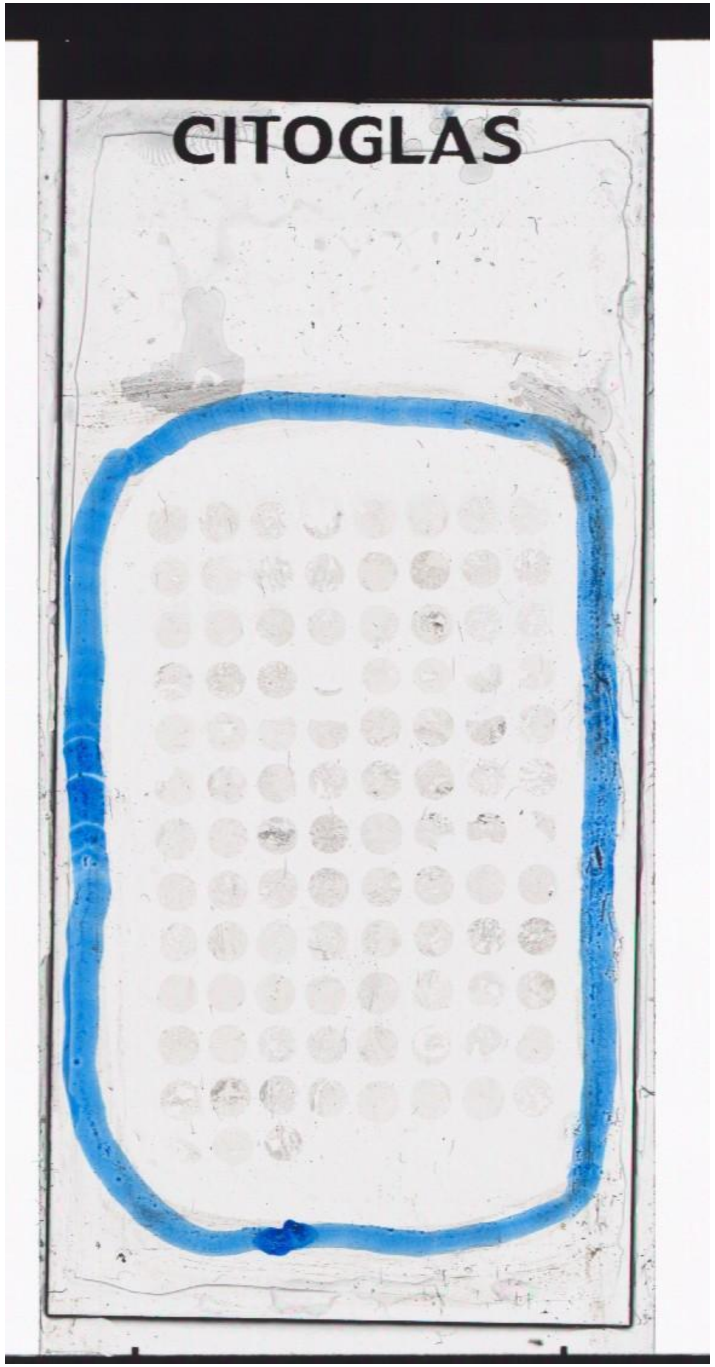

Panel 2

TMA 04

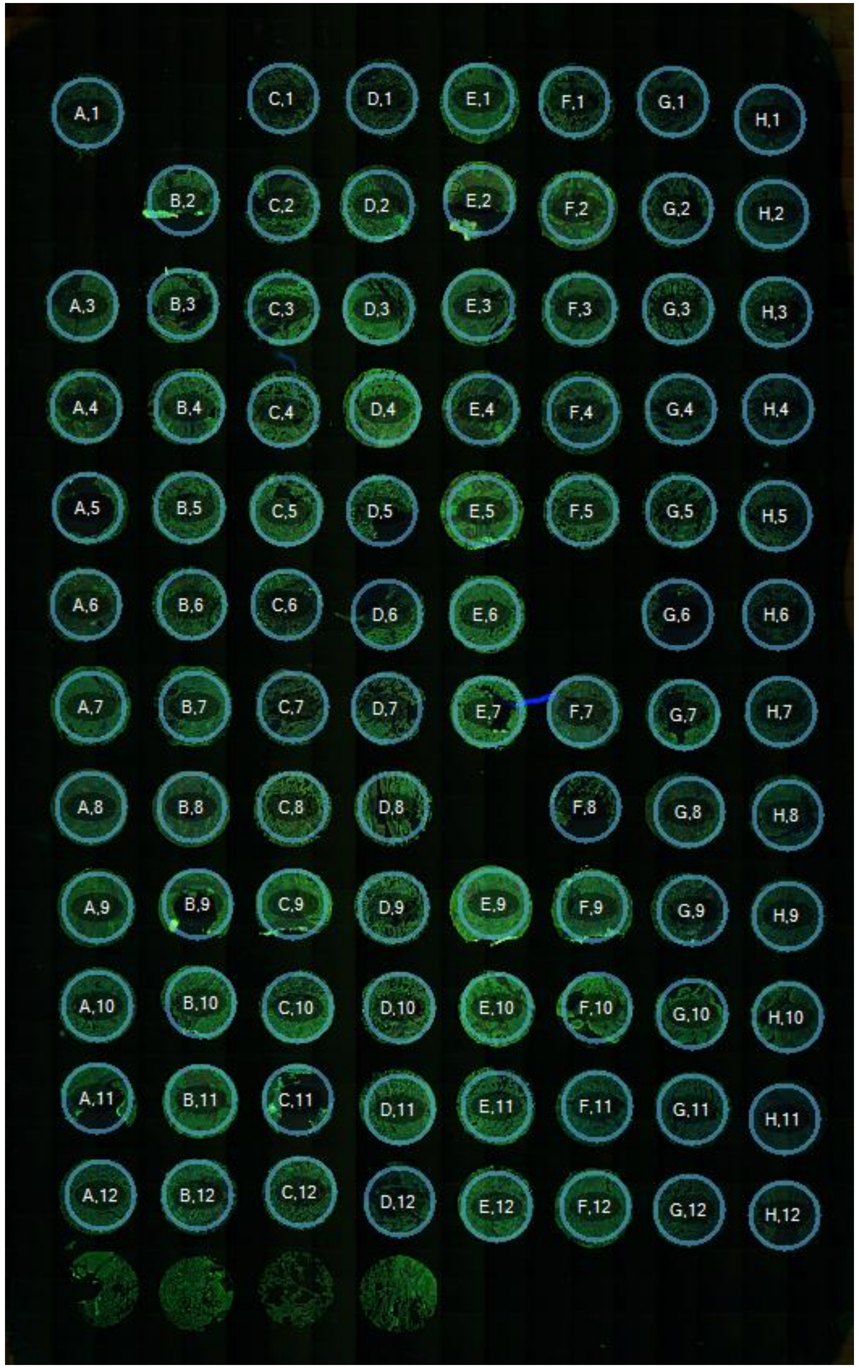

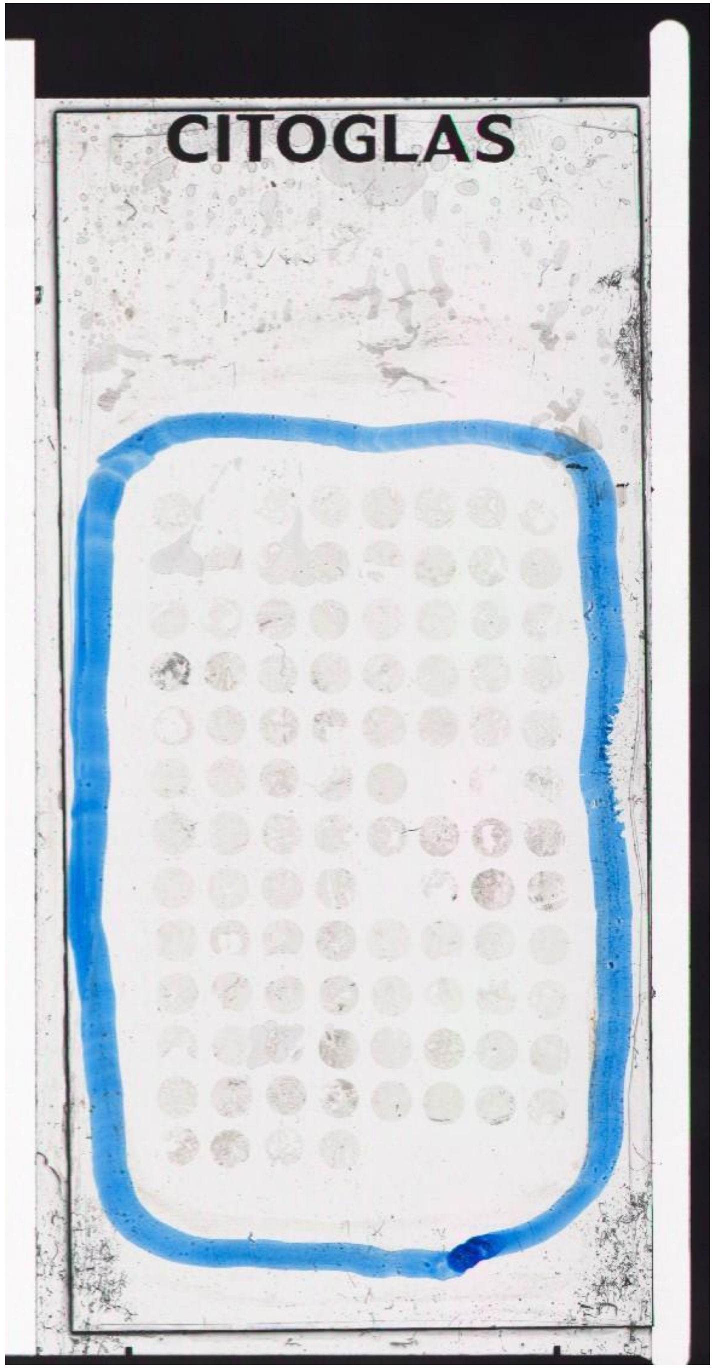

Panel 2

TMA 05

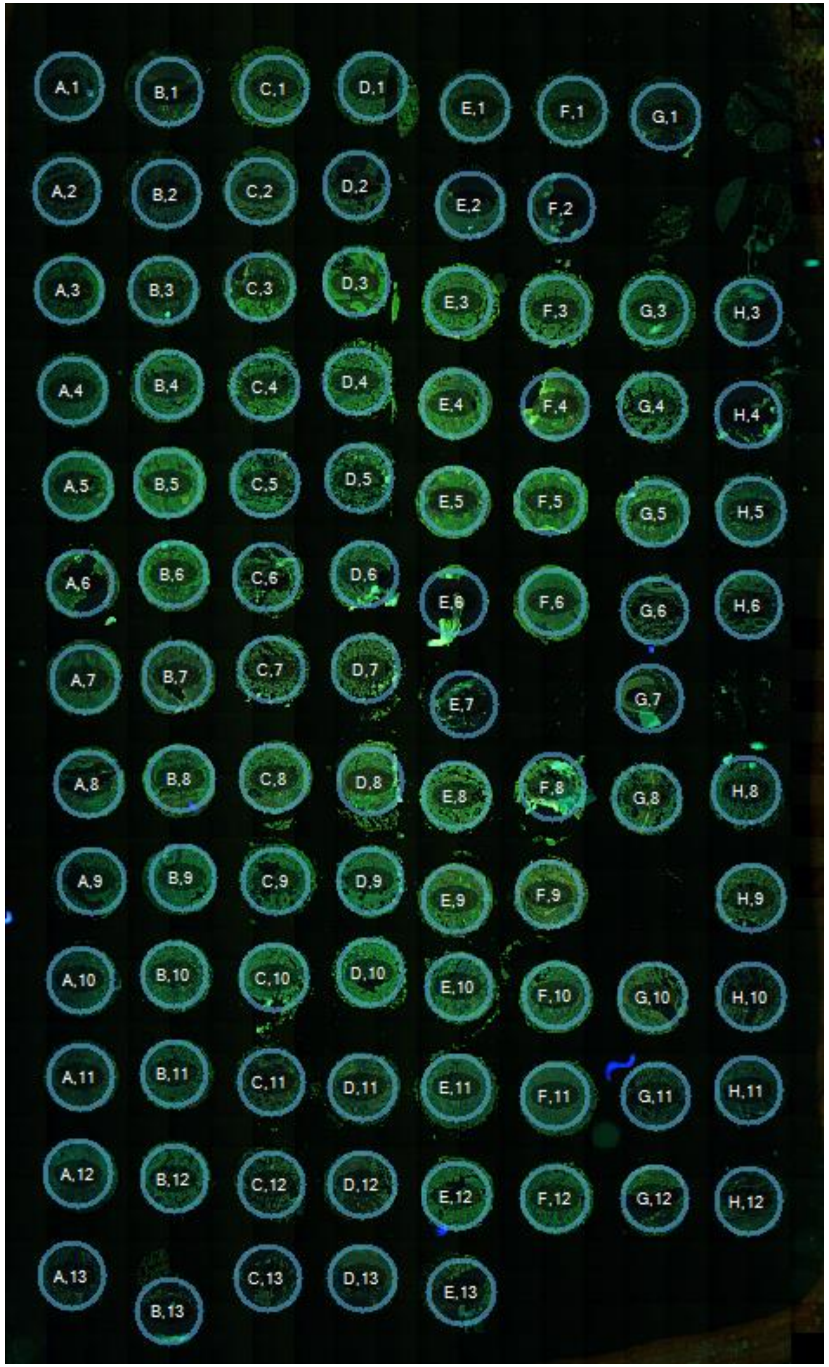

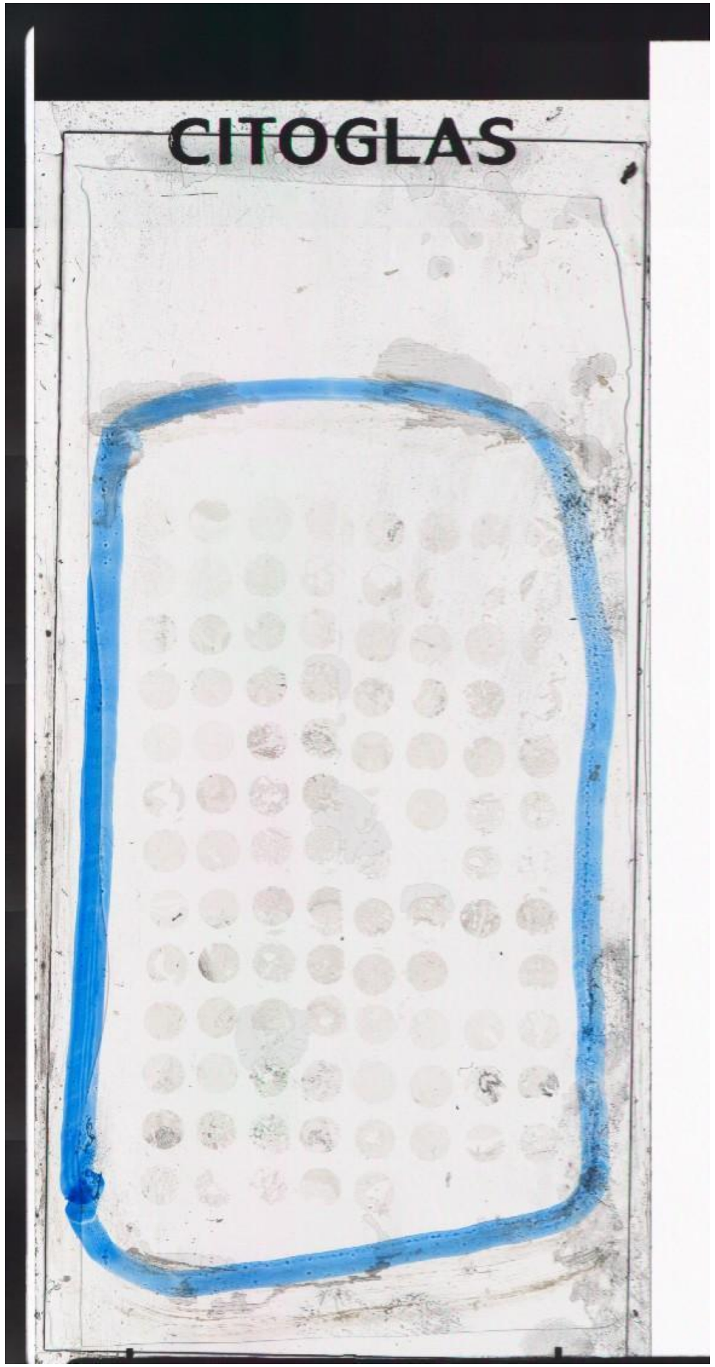

Panel 2

TMA 06

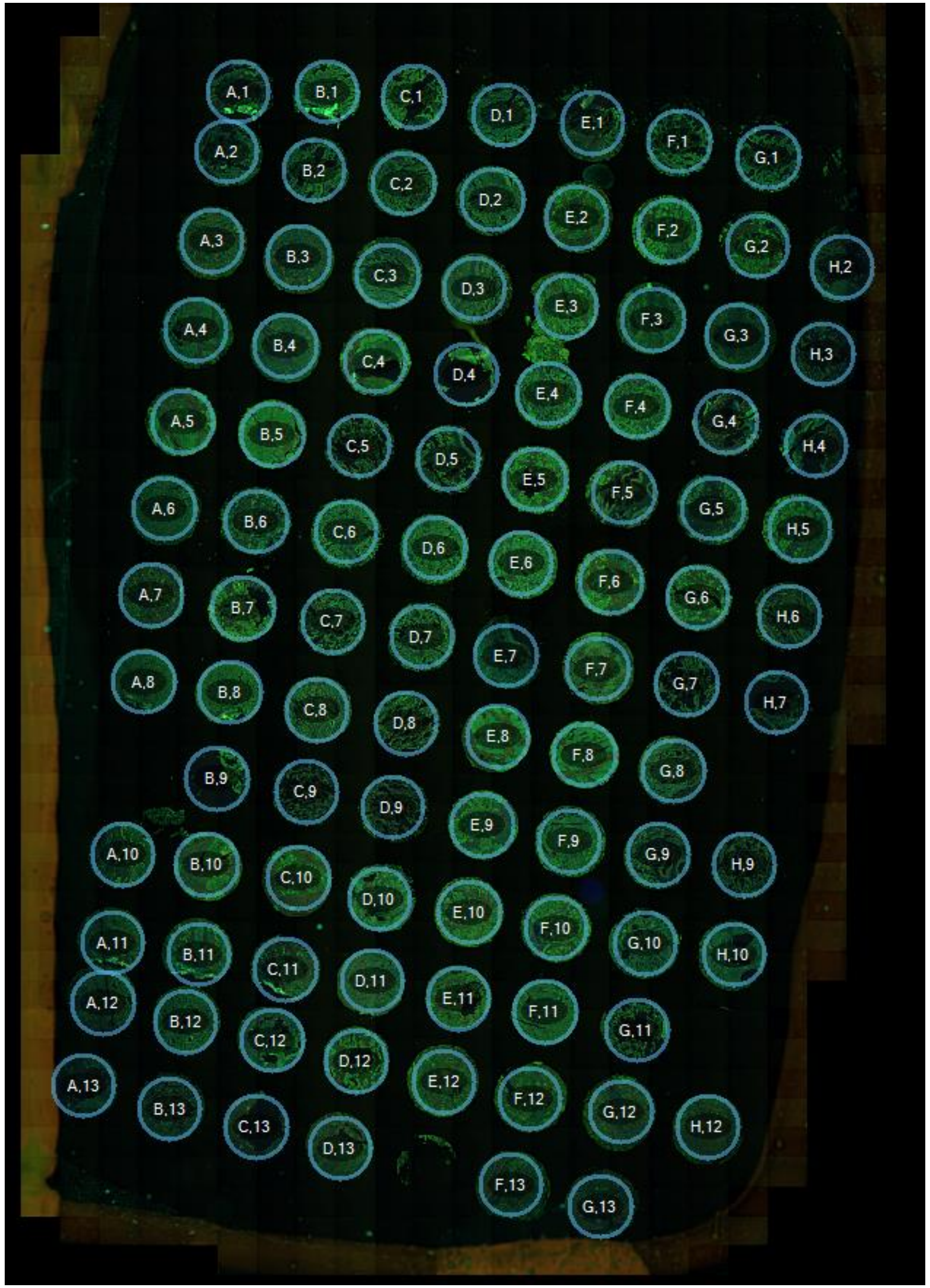

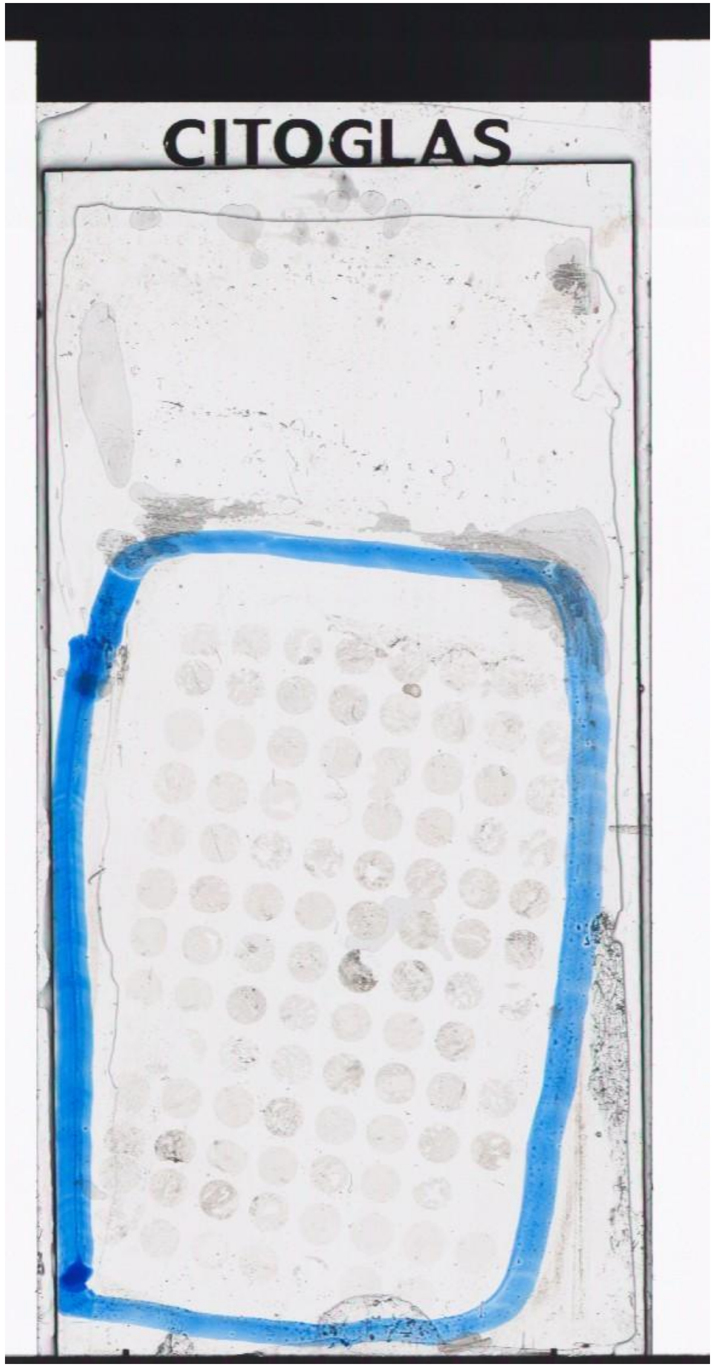

Panel 2

TMA 07

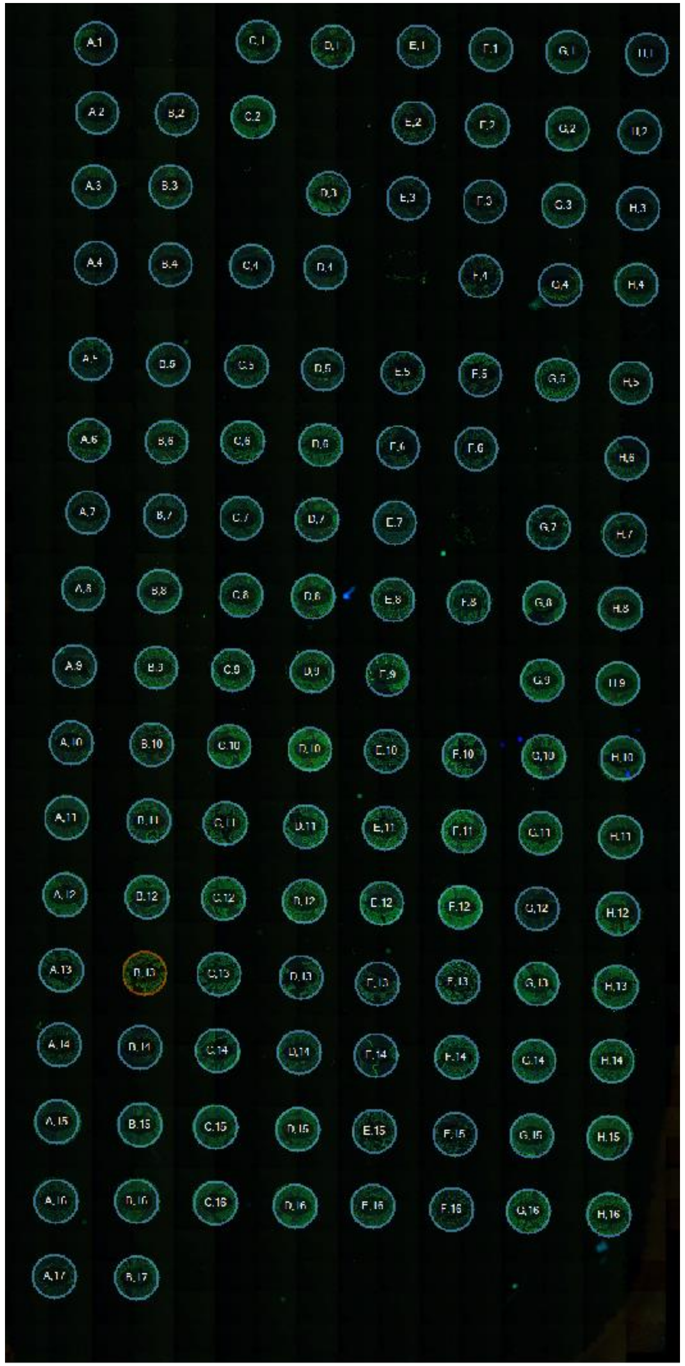

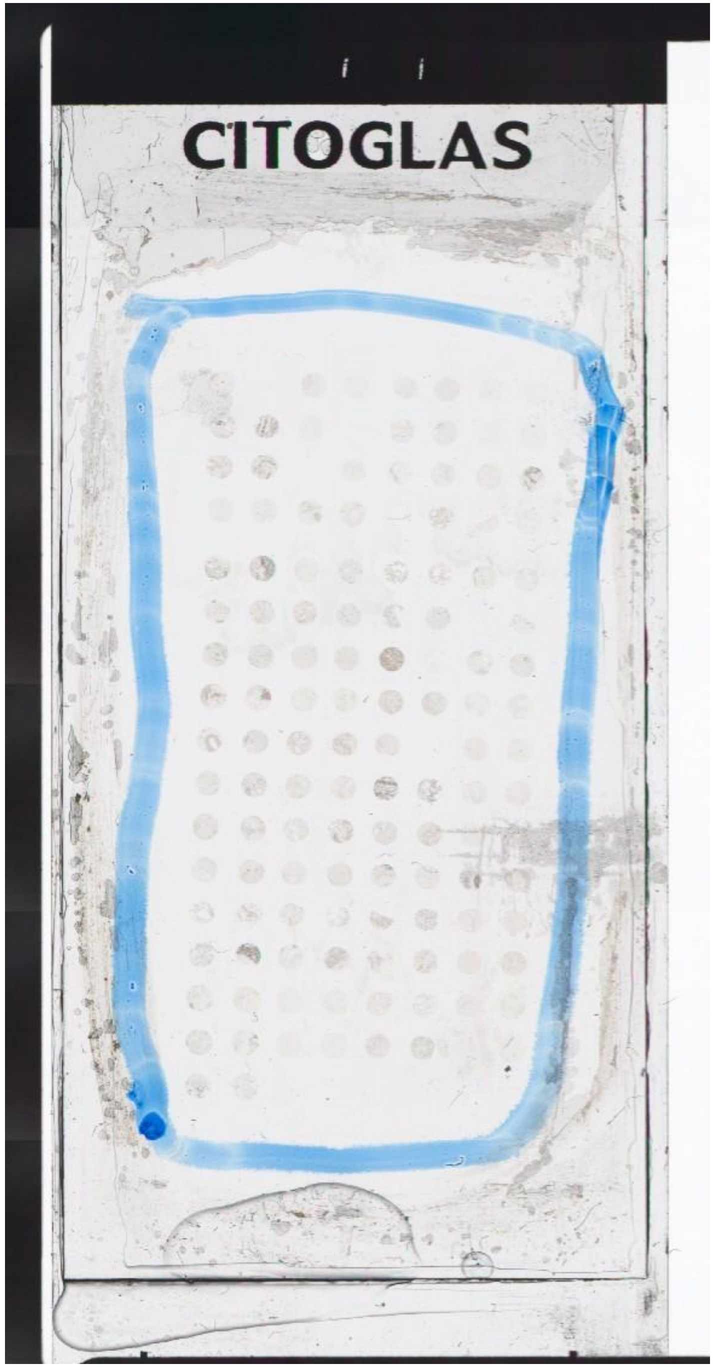

Panel 2

TMA 08

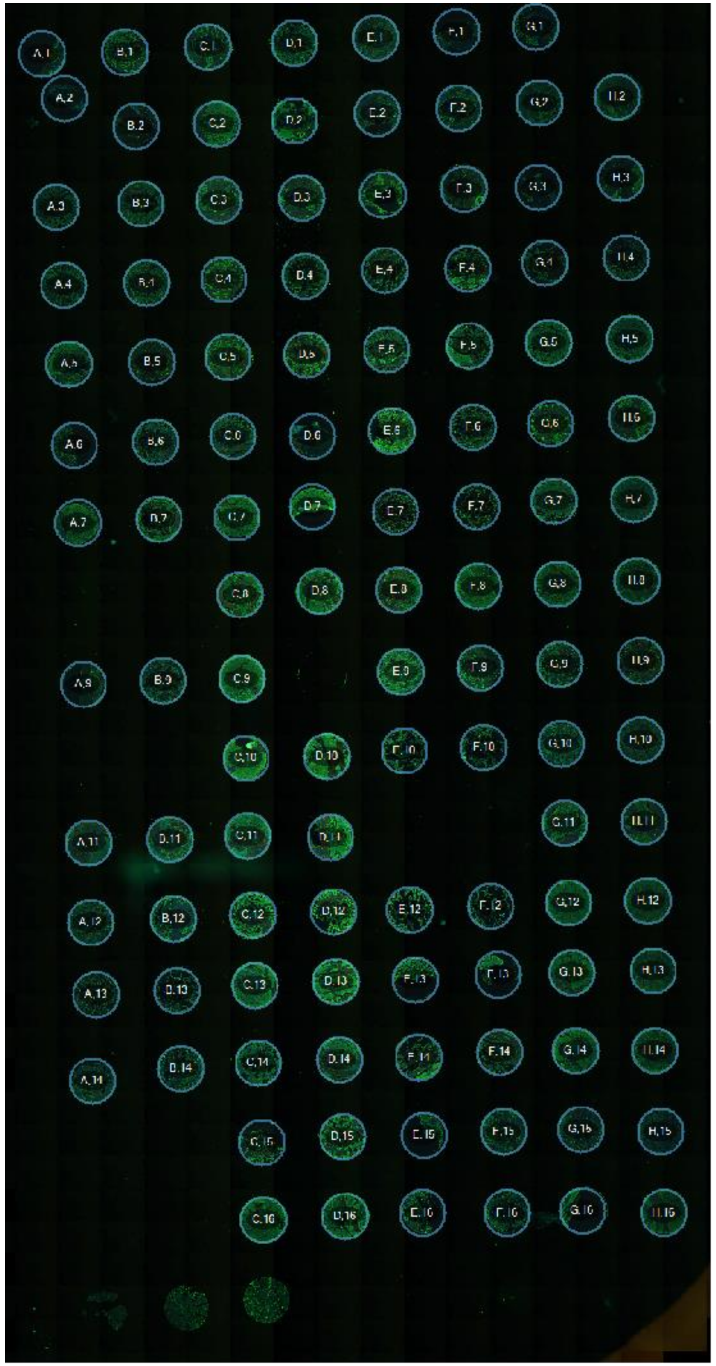

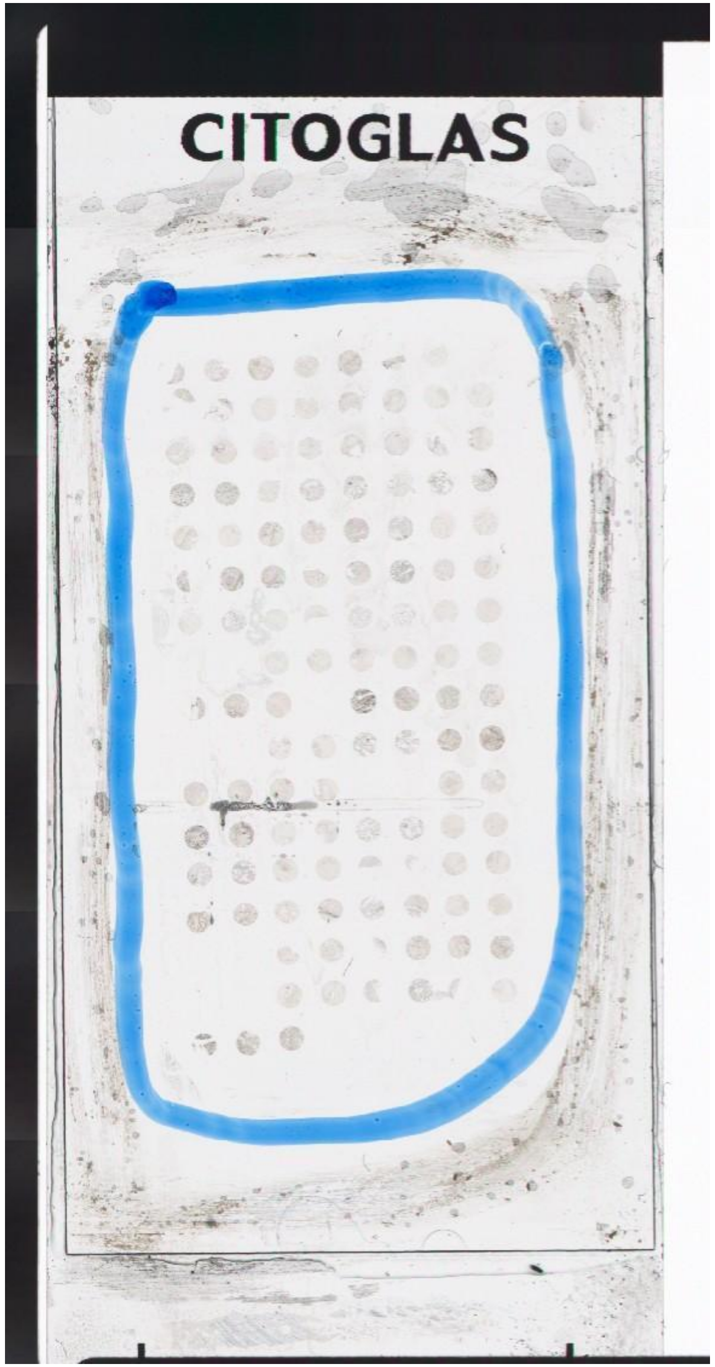

Panel 2

TMA 09

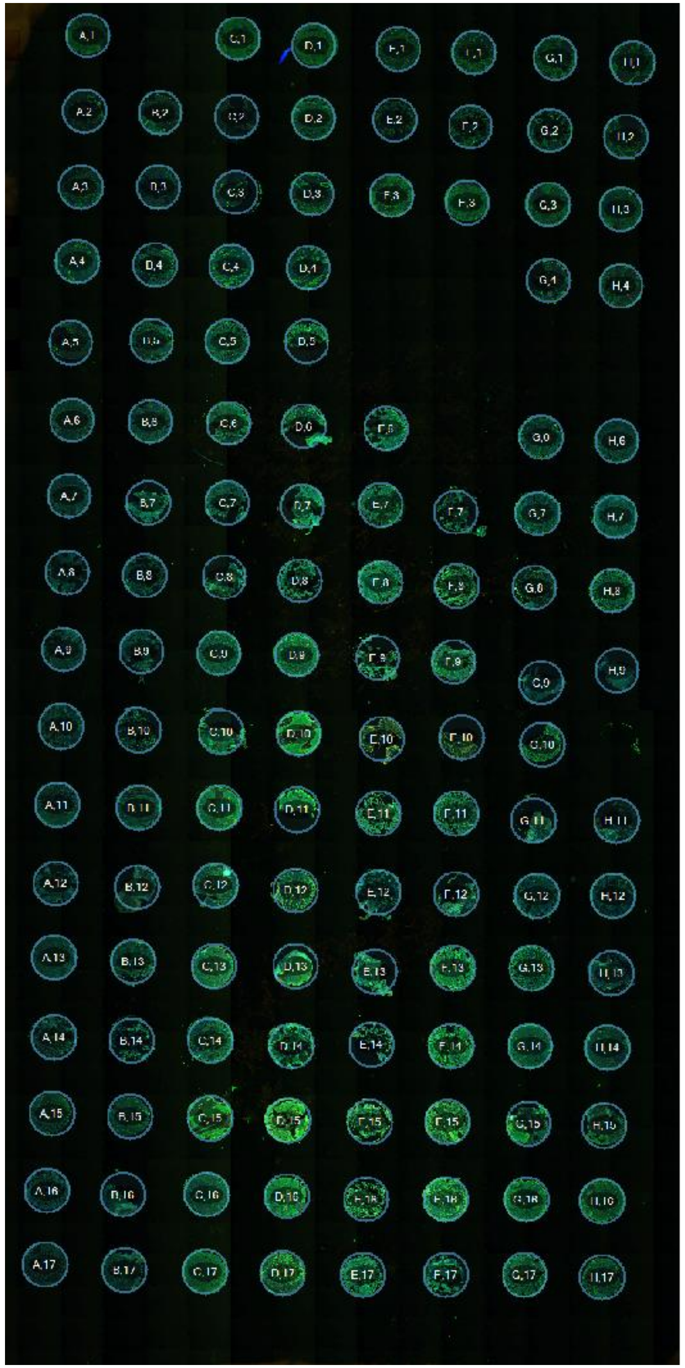

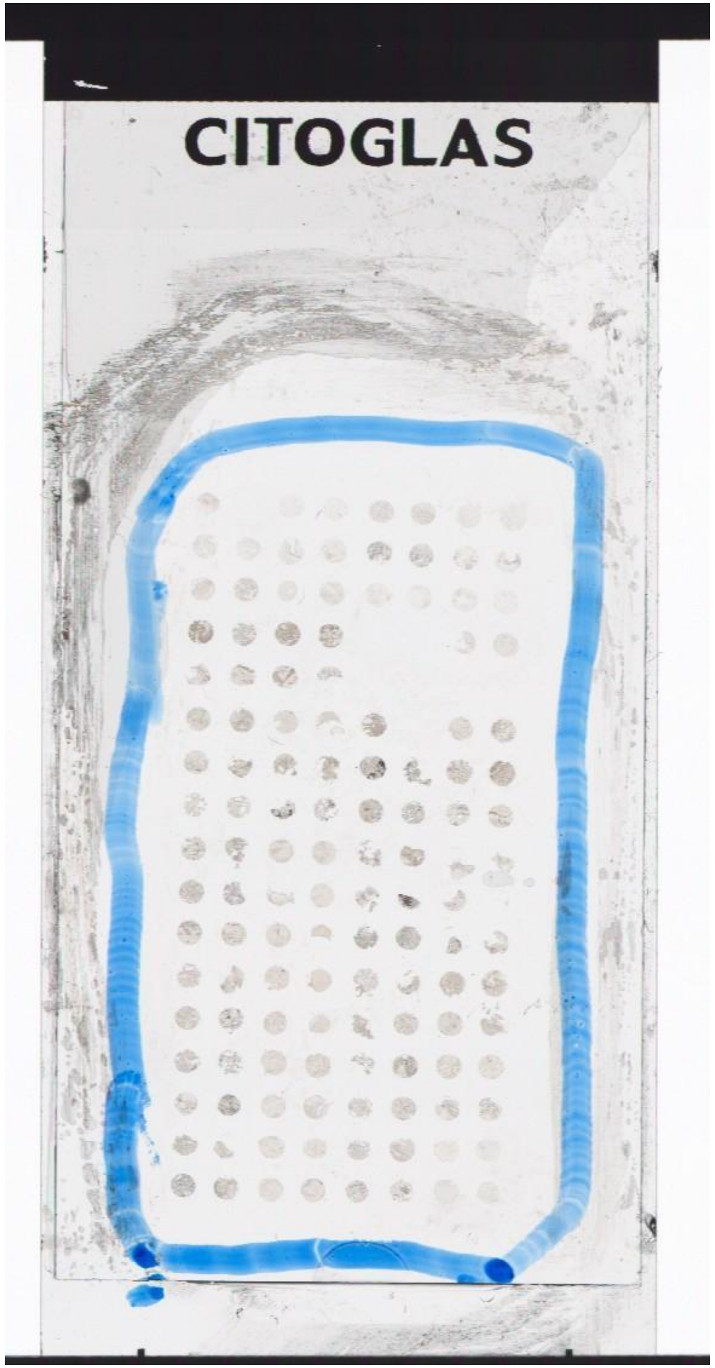

Panel 2

TMA 10

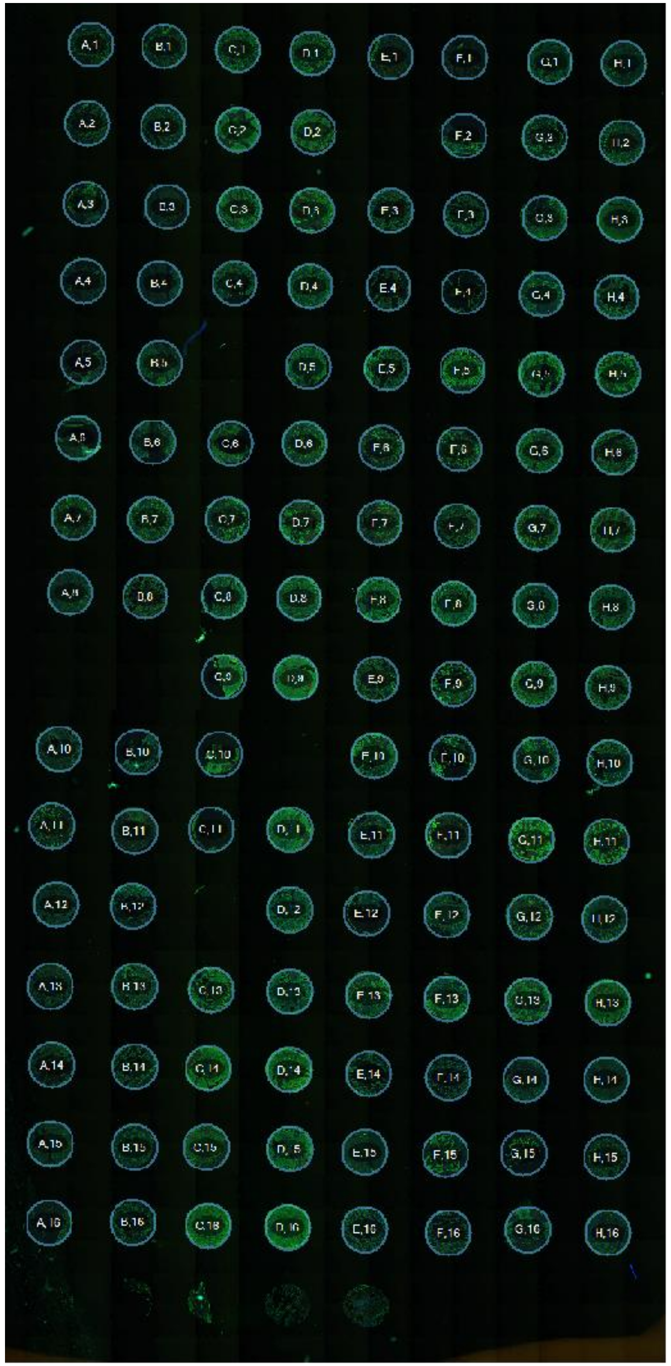

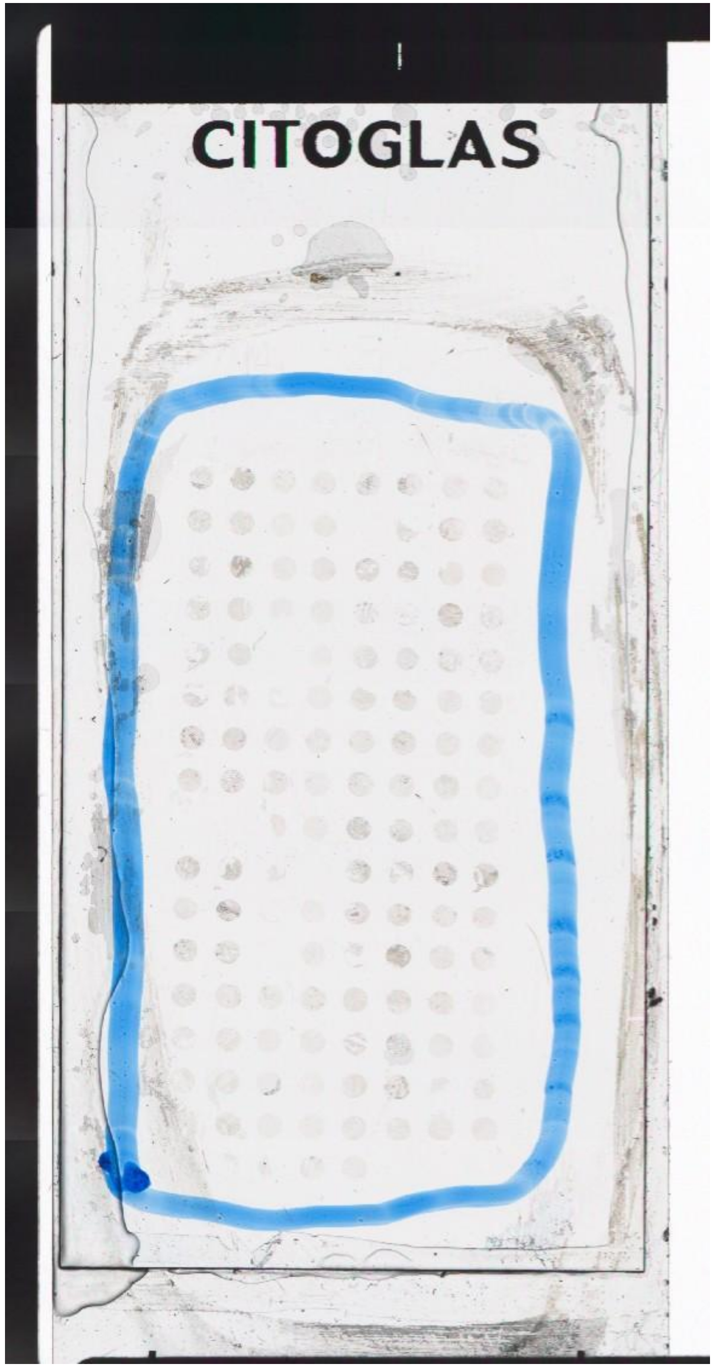

Panel 2

TMA 11

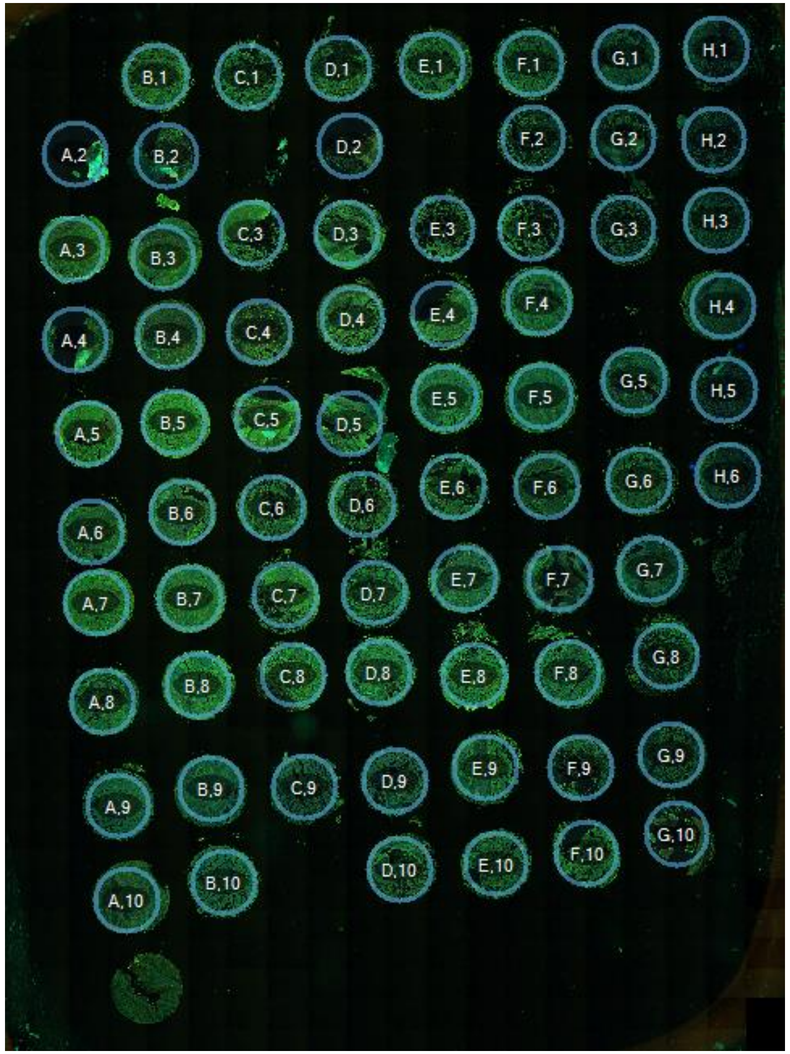

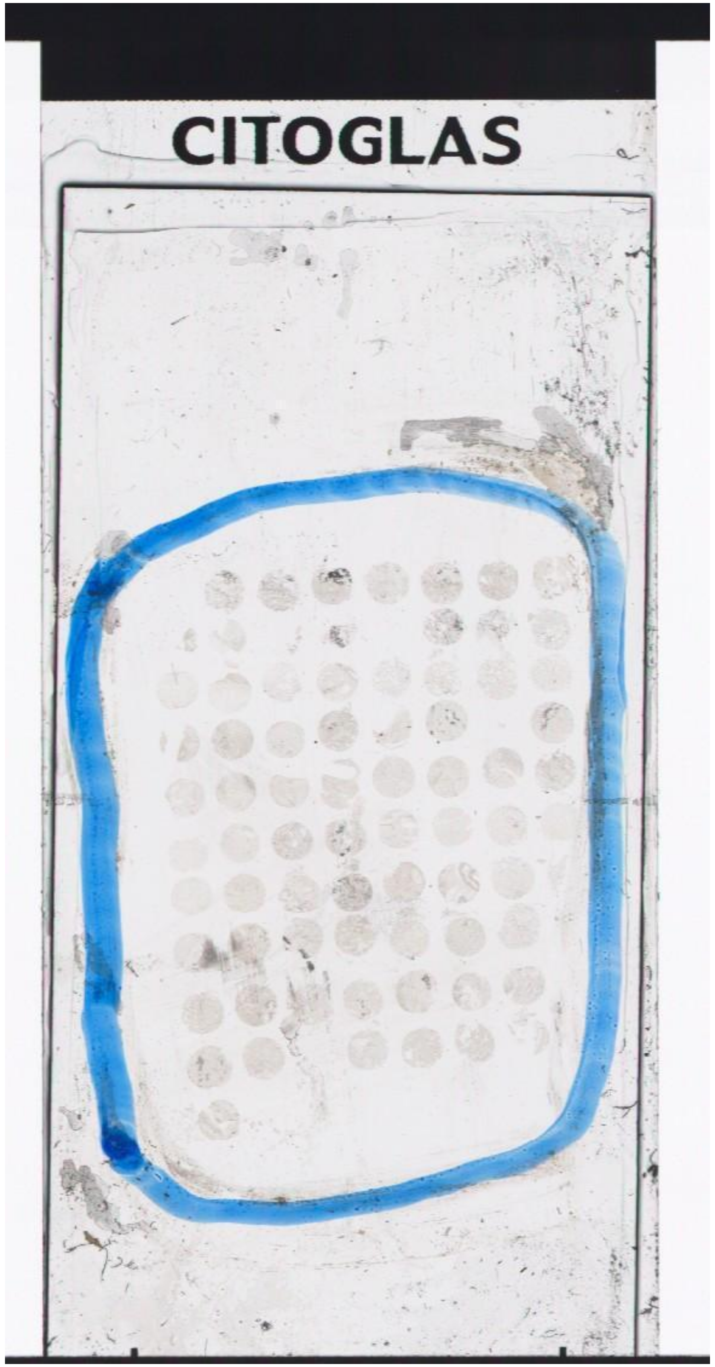

Panel 2

TMA 12

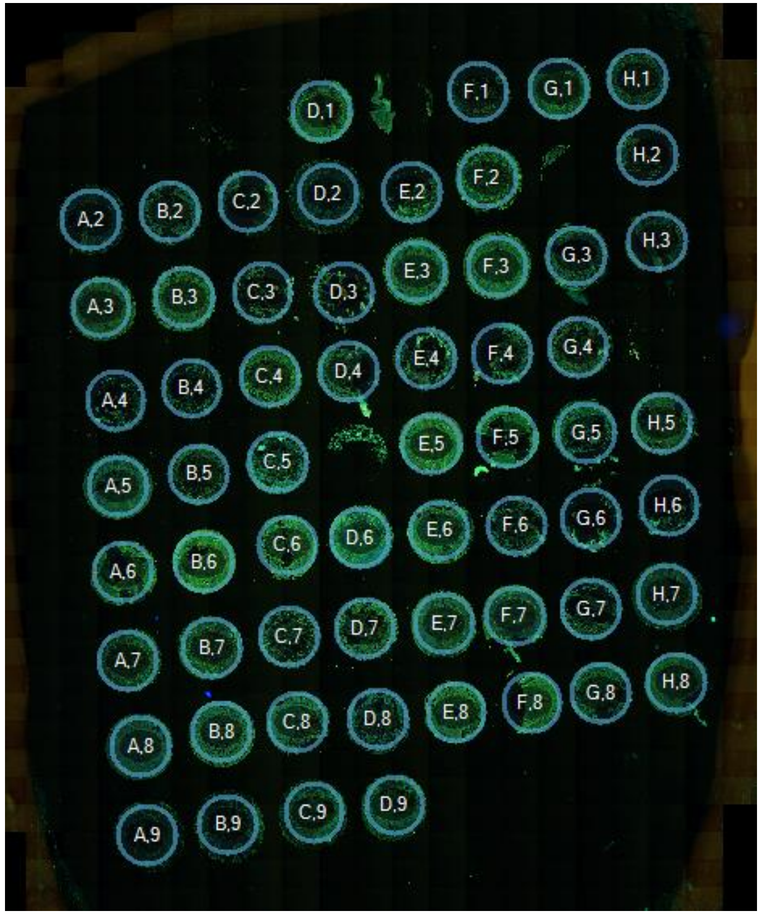

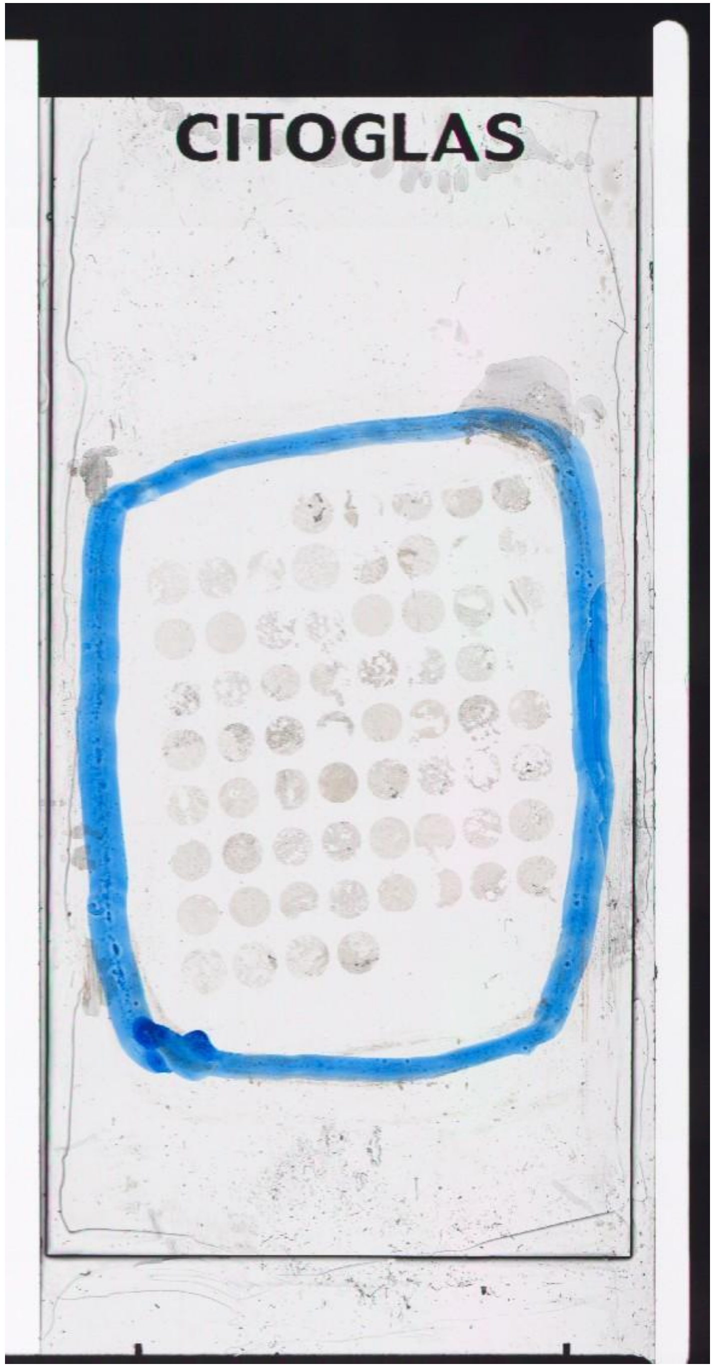

Panel 2

TMA 13

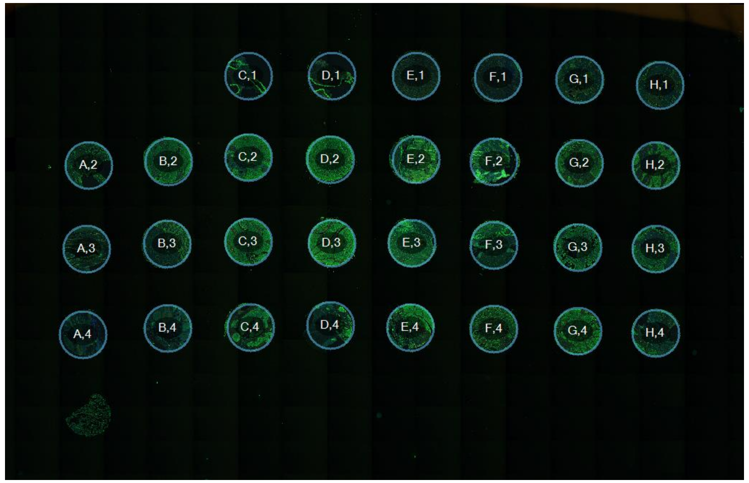

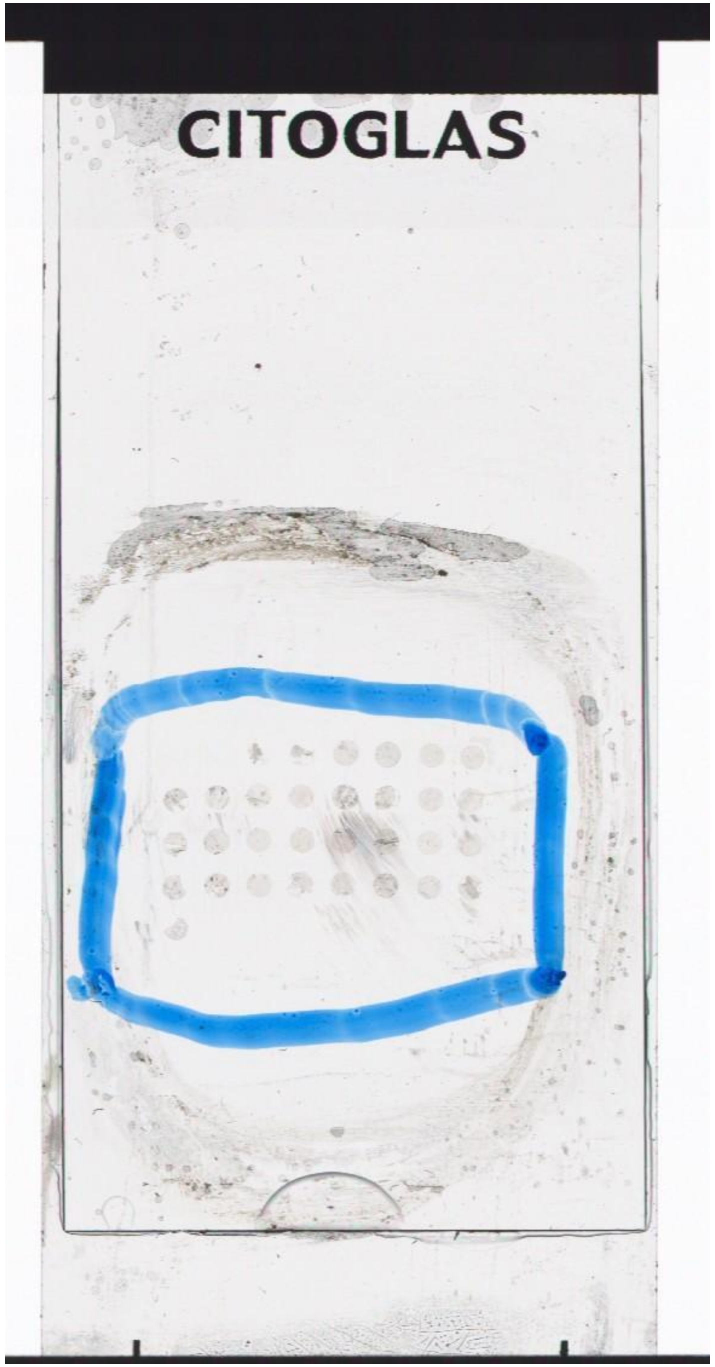

Supplement: Supplementary file 2 — Additional file 2. Overview of stained sections from all tissue microarray before and after fluorescence imaging. [file 12967_2023_4154_MOESM2_ESM.pdf]
